# Acute hepatitis of unknown aetiology in children: evidence for and against causal relationships with SARS-CoV-2, HAdv and AAV2

**DOI:** 10.1136/bmjpo-2023-002410

**Published:** 2024-12-09

**Authors:** Deepti Gurdasani, Mallory Trent, Hisham Ziauddeen, Emmanuel Mnatzaganian, Stuart Turville, Xin Chen, Mohana Priya Kunasekaran, Abrar Ahmad Chughtai, Aye Moa, Julie McEniery, Trisha Greenhalgh, Chandini Raina MacIntyre

**Affiliations:** 1 William Harvey Research Institute, Queen Mary University, London, UK; 2 Kirby Institute, University of New South Wales, Sydney, New South Wales, Australia; 3 University of Western Australia, Perth, Western Australia, Australia; 4 Department of Psychiatry, University of Cambridge, Cambridge, UK; 5 Department of Psychiatry, Fiona Stanley and Freemantle Hospitals, Perth, Perth, Australia; 6 University of New South Wales, Sydney, New South Wales, Australia; 7 School of Population Health, University of New South Wales, Sydney, New South Wales, Australia; 8 Faculty of Medicine, University of Queensland, Brisbane, Queensland, Australia; 9 Nuffield Department of Primary Care Health Sciences, University of Oxford, Oxford, UK

**Keywords:** COVID-19, Statistics, Epidemiology, hepatitis, paediatric

## Abstract

**Background:**

The cause of acute paediatric hepatitis of unknown aetiology (2022) has not been established despite extensive investigation.

**Objective:**

To summarise the evidence for and against a causal role for human adenovirus (HAdv), adeno-associated virus 2 (AAV-2) and SARS-CoV-2 in outbreaks of paediatric hepatitis in 2022.

**Methods:**

We appraised and summarised relevant evidence for each of the Bradford Hill criteria for causality using quantitative (statistical modelling) and qualitative (narrative coherence) approaches. Each team member scored the evidence base for each criterion separately for HAdv, AAV-2 and SARS-CoV-2; differences were resolved by discussion. We additionally examined criteria of strength and temporality by examining the lagged association between SARS-CoV-2 positivity, respiratory HAdv positivity, positive faecal HAdv specimens and excess A&E attendances in 1–4 years for liver conditions in England.

**Results:**

Assessing criteria using the published literature and our modelling: for HAdv three Bradford Hill criteria (strength, consistency and temporality) were partially met; and five criteria (consistency, coherence, experimental manipulation, analogy and temporality) were minimally met. For AAV-2, the strength of association criterion was fully met, five criteria (consistency, temporality, specificity, biological gradient and plausibility) were partially met and three (coherence, analogy and experimental manipulation) were minimally met. For SARS-CoV-2, five criteria (strength of association, plausibility, temporality, coherence and analogy) were fully met; one (consistency) was partially met and three (specificity, biological gradient and experimental manipulation) were minimally met.

**Conclusion:**

Based on the Bradford Hill criteria and modelling, HAdv alone is unlikely to be the cause of the recent increase in hepatitis in children. The causal link between SARS-CoV-2, and to a lesser degree AAV-2, appears substantially stronger but remains unproven. Hepatitis is a known complication of multisystem inflammatory syndrome in children following COVID-19, and SARS-CoV-2 has been linked to increased susceptibility to infection post-COVID-19, which may suggest complex causal pathways including a possible interaction with AAV-2 infection/reactivation in hosts that are genetically susceptible or sensitised to infection.

**WHAT IS ALREADY KNOWN ON THIS TOPIC**

**WHAT THIS STUDY ADDS**

- This study appraises and summarises the evidence on paediatric hepatitis of unknown cause to-date and assesses the extent to which Bradford Hill criteria for causality are met for associations of SARS-CoV-2, HAdv and AAV-2 infection with this. Additionally, we conducted new analyses, examining the lagged association between SARS-CoV-2 and human adenovirus (HAdv) positive samples and excess A&E attendances for liver conditions in children in England during 2022. Collectively, our study suggests stronger evidence for a causal link with SARS-CoV-2 followed by AAV-2, and that HAdv is unlikely to be the sole cause of the case cluster observed. However, the cause remains unproven and would require further research.

**HOW THIS STUDY MIGHT AFFECT RESEARCH, PRACTICE OR POLICY**

- Our work suggests that paediatric hepatitis of unknown cause is likely to have been related to postinfection inflammation. This may have involved complex causal pathways, involving SARS-CoV-2 infection and/or AAV-2 infection or reactivation in genetically susceptible hosts. However, further work is needed to establish causality. This work provides the first comprehensive assessment of all evidence on the 2022 cluster of paediatric hepatitis of unknown cause to date, with a view to informing clinical practice as well as further research in this area, should clusters of cases be observed again in the future. It also provides a framework for evaluation of evidence for other epidemiological clusters of unknown cause, to guide patient management and research.

## Introduction

 On 15th April 2022, the WHO issued a report on temporally clustered cases of severe acute hepatitis of unknown cause in children, predominantly under 5 years of age, in the UK.^1 2^ Clinicians were urged to identify and report probable cases, defined as anyone 16 years or younger presenting with acute hepatitis (with serum aspartate or alanine transaminase greater than 500 IU/L) since 1 October 2021.^3^ By 8th July, the WHO had identified 1,010 probable cases from 35 countries, at least 38 of whom required liver transplants.^4^ The epidemic curve for hepatitis peaked in late April 2022 and has declined since.^5^

Probable cases in this cluster were negative for hepatitis viruses A–E, and to date, no other previously known risk factors for hepatitis have been identified. However, several cases have tested positive for human adenovirus (HAdv), adeno-associated virus 2 (AAV-2) and SARS-CoV-2.^4 6 7^

The UK cluster of paediatric hepatitis was first identified soon after a period of rapid and unprecedented high exposure of young children to SARS-CoV-2 during the Omicron wave. The Omicron BA.1 and BA.2 waves peaked in January and March 2022 in the UK, and much of Europe.^8^ Between December 2021 and April 2022, it was estimated that 50% of children in England had been infected.^9^

The aim of this study was to examine the role of SARS-CoV-2, HAdv and AAV-2 in paediatric hepatitis, using two approaches; applying the Bradford Hill criteria to evaluate the evidence for a causal relationship^10^ and examining the temporal association between HAdv and SARS-CoV-2 positivity and paediatric hepatitis cases in the UK using distributed lag modelling (DLM). Our research question was ‘What is the strength of evidence for and against a causal role of SARS-CoV-2 and HAdv (±AAV-2) in acute severe hepatitis of unknown aetiology in children?’.

## Methods

### Literature search

The literature was searched for associations of hepatitis with SARS-COV-2, HAdV and AAV-2. As AAV-2 is a dependovirus, for which evidence supporting a possible association with paediatric hepatitis emerged during the course of our assessment, we also considered it as a potential cause, along with HAdv or other permissive viruses, as it is unable to replicate or cause illness in isolation.

To extend the sample of studies known to the authors, PubMed and Embase were searched using the following terms:

(((COVID-19[Mesh] OR SARS-CoV-2[Mesh]) AND (acute hepatitis OR liver injury)) OR (adenovirus AND (acute hepatitis OR liver injury)) OR (adenovirus hepatitis))

We included articles if they were a case report or case series of acute hepatitis associated with either virus in children, if they investigated an association between either virus and hepatitis, or if they investigated a biological link between liver manifestations and either virus. We reviewed the reference lists of selected articles for additional studies that met the above criteria. We undertook further searches to explore specific hypotheses which emerged during the study, notably an additional search on AAV-2 and multisystem inflammatory syndrome of childhood (MIS-C) using the relevant MeSH terms, and cross-referencing across article references. As this was a rapidly evolving situation, we regularly searched for new articles after May 2022 and included any that met the selection criteria.

### Bradford Hill criteria

Table 1 lists nine criteria used to assess the strength of evidence for a causal relationship between an exposure and a health outcome.^10^

**Table 1:** Definitions of Bradford Hill criteria used in this analysis

Indicator	Explanation
Strength of association	A strong association is more likely to be causal than a weak one
Consistency	Multiple observations made by different observers with different instruments mean the association is more likely to be causal
Specificity	If an outcome is best predicted by one primary factor, the causal claim is more credible
Temporality	A cause must precede an effect
Biological gradient	There should be a direct ‘dose–response’ relationship between the independent variable (eg, a risk factor) and the dependent one (eg, people’s status on the disease variable)
Plausibility	An association is more likely to be causal if there is a rational and theoretical basis for it
Coherence	An association is more likely to be causal if it coheres with other knowledge (ie, does not conflict with what is known about the variables under study and there are no plausible competing theories or rival hypotheses)
Experimental manipulation	Any related research that is based on experiments will make a causal inference more plausible
Analogy	Sometimes a commonly accepted phenomenon in one area can be applied to another area

### Application of Bradford Hill criteria

Using methodology described previously by Dyda *et al*,^11^ we summarised the key findings from each article in a spreadsheet and assessed their relevance to each of the criteria. For both agents, we considered the evidence for both acute infection (occurring close to the date of acute hepatitis) and a postinfection syndrome (where hepatitis may appear weeks or months later). Then, all study team members independently reviewed the evidence for each criterion and made a semiquantitative assessment as to whether it had been fully met (+++), partially met (++), minimally met (+) or not met (−). Where assessors disagreed, the evidence was discussed until a consensus was reached. Below, we set out how we assessed each criterion. We generated a semiquantitative score across all criteria to judge the evidence on causality for each exposure. Given each criterion could have a minimum score of 0 (not met), and maximum of 3, fully met (reaching a maximum of 27 across 9 criteria), we specified categories for evidence of causality as 0–6 (unlikely to be causal), 7–13 (weak evidence), 14–20 (moderate evidence) and 21 and above (strong evidence).

#### Additional analysis of temporal relationship (temporality) and strength of association

If HAdv, AAV-2 and/or SARS-CoV-2 were causal agents for unknown paediatric hepatitis we would expect to see an association between HAdv positivity, AAV-2 and/or SARS-CoV-2 positivity and the incidence of these hepatitis cases. Ascertainment of these cases of hepatitis is expected to be better from April 2022 onwards when this clinical presentation was first reported. To deal with potentially poorer retrospective ascertainment, one can look at excess cases that is, cases over those expected based on previous years’ data. The UK Health Security Agency (UKHSA) had reported a clear excess of A&E attendances for 1–4 years for liver conditions in 2022 compared with previous years.^12^ We, therefore, examined the association of SARS-CoV-2 and HAdv positivity with excess emergency department (ED) attendances with liver conditions in children aged 1–4 years in England, the group with the most number of unknown paediatric hepatitis and thus most likely to have the strongest signal and lowest signal-to-noise ratio, given the small sample size. AAV-2 surveillance is not routinely carried out so we could not assess this separately as an exposure in our modelling analysis. However, we did assess this through our review of literature and assignment of scores based on Bradford Hill criteria, as with HAdV and SARS-CoV-2.

### Outcome

**Figure 1:**
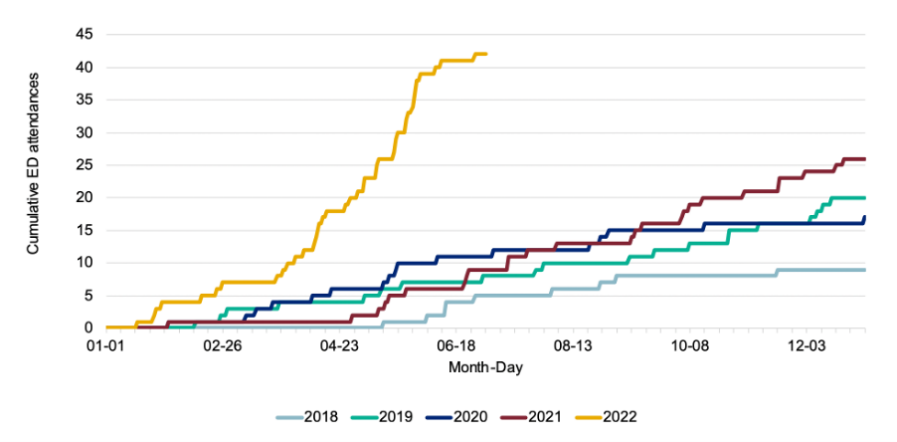
Cumulative daily number of ED attendances** for children aged 1–4 years, with a ‘liver condition’* primary diagnosis, 1 April 2018 to 3 July 2022 (reproduced from UKHSA Technical report 4). *‘Liver conditions’ primary diagnoses include inflammatory disease of the liver (46%), hepatic failure (32%), injury of liver (16%), acute infectious hepatitis (3%), viral hepatitis A (1%), viral hepatitis B (1%), acute hepatitis caused by infection (<1%). **ED attendances as identified by syndromic surveillance, including 104 EDs: • type 01 ED attendances only • limited to EDs which started reporting through this route during 2018 • ED syndromic surveillance reporting through the route reported here commenced April 2018 • limited to EDs which reported quickly and frequently in the most recent week (received data for 7 out of 7 of the days 27 June to 3 July 2022, and the data arrived with the UKHSA Realtime Syndromic Surveillance Team within 2 calendar days of the patient attendance) • EDs are excluded where historical issues with diagnosis coding have been identified. ED, emergency department; UKHSA, UK Health Security Agency.

The outcome of interest was excess ED attendances with liver conditions in 1–4 years during 2020, 2021 and 2022 compared with previous years. ED attendance data were collected by the UKHSA syndromic surveillance system which started in April 2018. This includes 104 EDs and is limited to attendances at type 01 EDs (most comprehensive level of ED service). Liver conditions included the following primary diagnoses: inflammatory disease of the liver (46%), hepatic failure (32%), injury of liver (16%), acute infectious hepatitis (3%), viral hepatitis A (1%), viral hepatitis B (1%) and acute hepatitis caused by infection (<1%). Data for the period 1 April 2018 to 3 July 2022 (figure 16A in technical briefing 4)^12^ (figure 1) were extracted using WebPlotDigitizer (https://automeris.io/WebPlotDigitizer/citation.html), a tool proven to be reliable and valid for extracting numerical data from plots.^13^ To calculate excess attendances to Accident and Emergency (A&E), the period from April 2018 to December 2019 was considered as baseline. The 2018 and 2019 data were averaged by month. Excess deaths for 2020, 2021 and 2022 were calculated with respect to this baseline.

### Exposures

We used case positivity, where available, rather than case numbers as our exposures as these are more robust to changes in testing behaviour and ascertainment bias (online supplemental figure 1a).

For SARS-CoV-2, we used data from the Office for National Statistics (ONS) COVID-19 infection survey,^14^ which is a random community household survey that samples large numbers of households in the UK weekly, reporting the percentage of the private-residential population that would have tested positive for COVID-19, otherwise known as the positivity rate. These estimates are more reliable than case numbers, as they are not influenced by testing behaviour. We used weighted estimates of SARS-CoV-2 positivity for non-overlapping 14-day periods published by the ONS for the 2-year-old to school year 6 age group. As these were only available until 13 May 2022, we averaged daily SARS-CoV-2 positivity data over non-overlapping 14-day periods to derive consistent estimates following this.

For HAdv, we used published respiratory adenovirus positivity estimates from the UKHSA for 1–4 years (online supplemental figure 1a). UKHSA data show that while identified cases of adenovirus through respiratory and gastroenteritic sampling have increased considerably in 2022, this is likely due to better ascertainment of cases. Positivity estimates would be less influenced by ascertainment but these were only available for respiratory samples (online supplemental figure 1a) and not faecal samples. So, in addition to respiratory sample positivity, for completion, we also considered the number of positive faecal specimens in 1–4 years as an exposure, acknowledging the potential ascertainment bias. We note that respiratory and gastroenteritic sampled adenovirus cases, as reported by UKHSA followed the same pattern, and were highly correlated (online supplemental figure 1b,c). Positivity data were extracted from the UKHSA Technical reports using WebPlotDigitizer.

### Statistical analysis

In order to examine the temporal correlation between the prespecified exposures and outcome, we used distributed lagged models (DLMs).^15^ DLMs allow regression of one time series (dependent variable) on another (independent variable), estimating the distribution of lags between them, as these are considered unknown. These lags can be constrained to fit a polynomial distribution, or allowed to vary, but with a finite cap in time (see online supplemental methods). We considered a number of predefined models (online supplemental table 1) distributed lag linear and non-linear models (DLMs and DLNMs), allowing for non-linear relationships between exposure and outcome, and lag and outcome. We considered 20 weeks (10 periods of 2 weeks each) as the maximum lag for effect (q), and weights beyond this lag were considered 0. A seasonal effect was included in all models as a natural cubic spline with three internal knots (13, 26 and 39 weeks) for each year.

We applied these to the period 26 April 2020 to 2 July 2022 to allow comparability as positivity data were available for both exposures during this period. Models were assessed by comparing their Akaike information criterion (AIC). We also calculated mean squared error (MSE) between observed and predicted values across all models to examine predictive accuracy. For linear exposure functions, we assessed significance of individual coefficients by the Wald test (a test of the null $\beta_{s}=0$) with a prespecified significance threshold of two-tailed p<0.01 given the multiple parameter estimations. For the DLNMs, the bidimensional set of coefficients (lag weights and effect size) for the exposure was reduced across the lag dimension into the overall cumulative exposure-response curve between 2 weekly average exposure and excess hepatitis, and these coefficients were examined. Analyses were conducted using the DLagM and DLNM packages in R.

### Patient and public involvement

No patients were involved.

## Results

### Description of dataset

Our search identified 793 papers, of which 162 underwent full-text review and 89 were examined as relevant and included in the final manuscript. Taken together, these data allowed us to evaluate the Bradford Hill criteria (table 1) for each exposure, considering both acute exposure and postviral illness. We describe key findings below.

#### Summary of findings against Bradford Hill criteria

The evidence assessed against each of the Bradford Hill criteria for each virus is described in detail in online supplemental tables 2-4 for both acute infection (direct causal) and as postinfectious phenomena (indirect causal). The scoring of each of the nine criteria for SARS-CoV-2 and adenovirus and detailed rationale are summarised in table 2. The summary scores based on the literature search were 18 for SARS-CoV-2, 11 for adenovirus and 17 for AAV-2 (table 2).

**Table 2:** Scoring of the evidence for causation between acute hepatitis and SARS-CoV-2 and human adenovirus (HAdv) in children

Criterion		Evidence search			Evidence search+analysis	
	SARS-CoV-2	HAdv	AAV-2	SARS-CoV-2	HAdv	AAV-2
Strength of association	++	++	+++	+++	++	+++
Consistency	++	++	++	++	++	++
Specificity	+	+	++	+	+	++
Temporality	++	++	++	+++	++	++
Biological gradient	+	+	++	+	+	++
Plausibility	+++	–	++	+++	–	++
Coherence	+++	+	+	+++	+	+
Experimental manipulation	+	+	+	+	+	+
Analogy	+++	+	+	+++	+	+
Total score	18	11	16	20	11	16

+++ criterion fully met.

++ criterion partially met.

+ criterion minimally met.

– criterion not met.

### Additional analysis, England

#### Examination of temporal relationship and strength of association in England

Examining cumulative excess A&E attendances in the age group 1–4 years for liver conditions (figure 1), we find 29.5 excess attendances between 2020 and 3 July 2022 (online supplemental figure 2). We see no excess in the year 2020, with attendances being below baseline (excess=−6.5). We see a small excess in 2021 (5.5), and a much larger excess in the year 2022 (30.5) (online supplemental figure 2).

### SARS-CoV-2 positivity and excess ED attendances with hepatitis in 1–4 years

**Figure 2:**
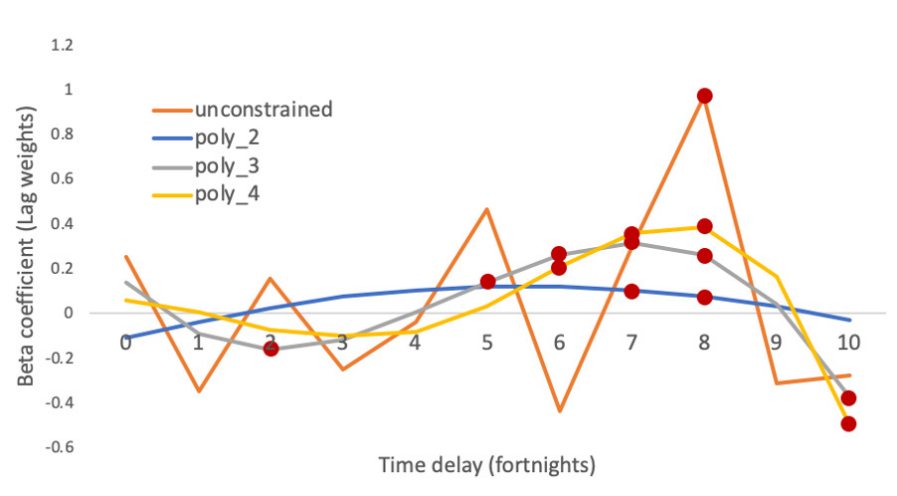
Lag weights using different models for SARS-CoV-2 positivity as exposure. Figure 2 shows the coefficients (lag weights) estimated for lags from the 1st to 10th fortnight considering a linear relationship between SARS-CoV-2 positivity and outcome, and unconstrained lags (orange line), polynomially distributed lag weights of degree 2 (blue line), 3 (grey line) and 4 (yellow line). Red dots indicate statistically significant lag weights with p<0.01.

**Figure 3:**
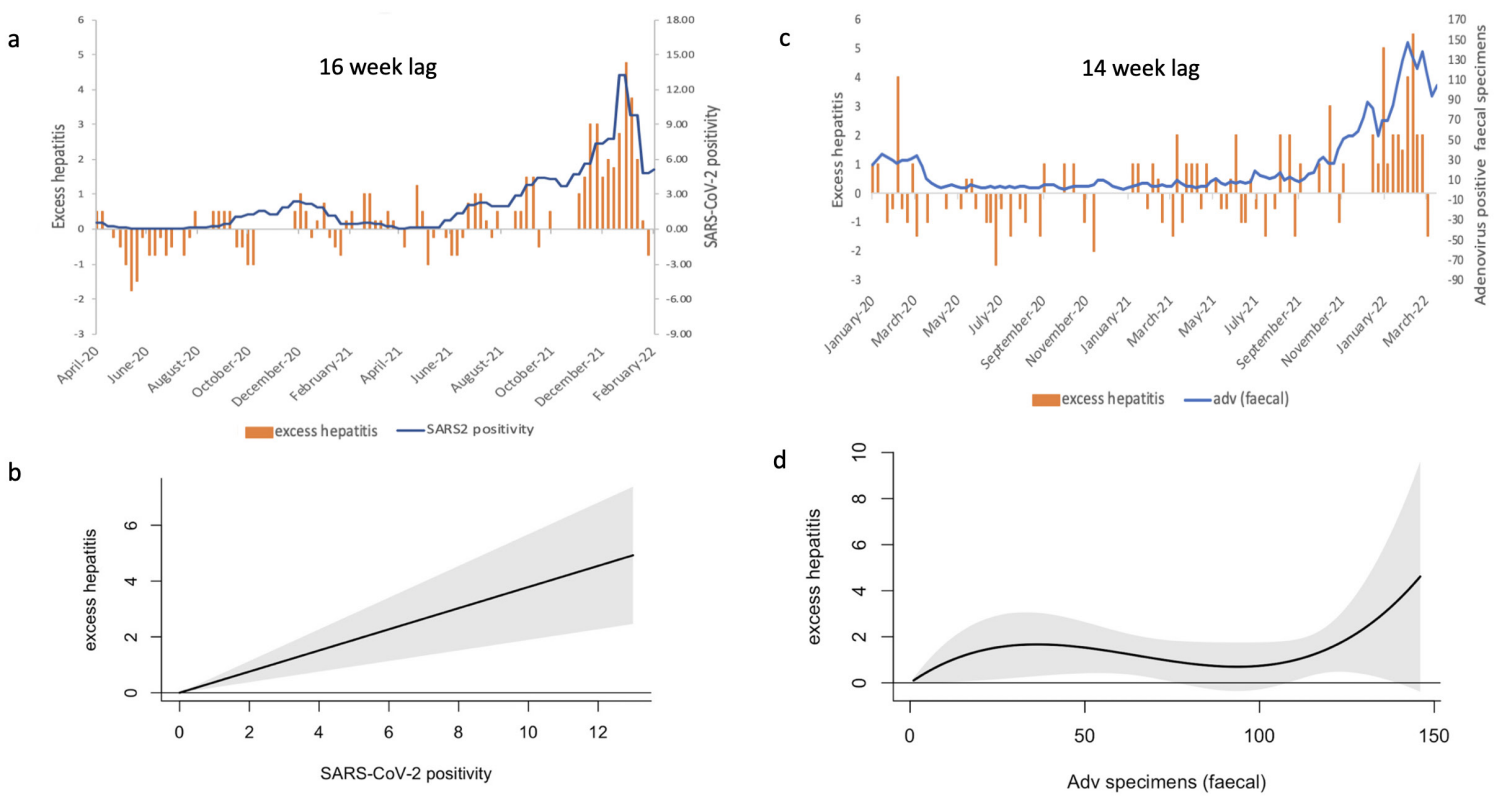
Estimated relationship between SARS-CoV-2 positivity, faecal HAdv cases and excess hepatitis. (a) SARS-CoV-2 positivity superimposed on excess attendances to Accident and Emergency (A&E) with liver conditions for 1–4 years, with a 16-week lag between SARS-CoV-2 positivity and excess attendances for liver condition, as this was the lag weight statistically significant in our most informative model. (**b**) The linear relationship modelled between SARS-CoV-2 positivity and excess hepatitis using unconstrained lag weights (the most informative model, as per AIC estimates). 95% CIs are shown in grey. (**c**) The 14-week lagged superimposed relationship between the number of positive faecal specimens for HAdv and excess hepatitis collapse across lag weights. (**d**) The non-linear relationship modelled between the number of faecal specimens positive for adenovirus and excess hepatitis consolidated across lag weights, using the most informative model as per AIC estimates. 95% CIs are shown in grey. AIC, Akaike information criterion.

Based on the AIC, the best model was model 1—a linearly modelled association between SARS-CoV-2 positivity and excess hepatitis (online supplemental table 5), with unconstrained lag weights up to a lag of 10 periods of 2 weeks each (up to 22 weeks). The overall exposure–outcome relationship was statistically significant, with the lag weight coefficients being statistically significant at 16–17 weeks (p=0.0004) and 20 weeks (p=0.01) (figure 2). All models consistently produced the most statistically significant lag weight at 14–17 (7–8 2-week lag) weeks (figures2  3a). Overall, the model estimated a 0.38 (95% CI 0.19 to 0.57) predicted overall increase in excess A&E admissions for a 1% increase in SARS-CoV-2 positivity (figure 3b). The correlation of excess hepatitis admissions against SARS-CoV-2 positivity with the statistically significant lag of 16 weeks as determined by the best fit for the model is represented in figure 3a.

### Adenovirus positivity and excess ED attendances with hepatitis in 1–4 years

The lowest AIC was for the most flexible model that used polynomials with 3 df to represent non-linear relationships between exposure and outcome, and a polynomial with 4 df to model lag weights (online supplemental table 5). The overall coefficients for the exposure–response relationship were not statistically different from zero along the range of respiratory adenovirus positivity (online supplemental figure 3). We note the high level of uncertainty around coefficients, likely due to sparse data and estimation of multiple coefficients. Therefore, for better interpretability, we also applied model 1 to adenovirus positivity, which included unconstrained lag effects, and considered a linear relationship between adenovirus positivity and excess hepatitis, even though this had a higher AIC (online supplemental table 2). Adenovirus positivity once again showed no statistically significant association with excess hepatitis. Lag weights were non-significant for all lags up to ten 2-week periods. Online supplemental figure 3 shows the adenovirus positivity rates superimposed on the excess hepatitis cases at lags of 2, 4, 8, 12, 16 and 20 weeks to illustrate the lack of association.

Given exactly the same number of time periods in our assessment of both exposures, these models did not differ in their power to detect a non-null effect. Therefore, we found no statistical evidence for an association between excess hepatitis and HAdv positivity in children aged 1–4 years. We note that the AIC and MSE scores for the most informative model with SARS-CoV-2 positivity were lower than those with adenovirus positivity (online supplemental table 2).

### Adenovirus faecal positive specimens and excess ED attendances with hepatitis in 1–4 years

**Figure 4:**
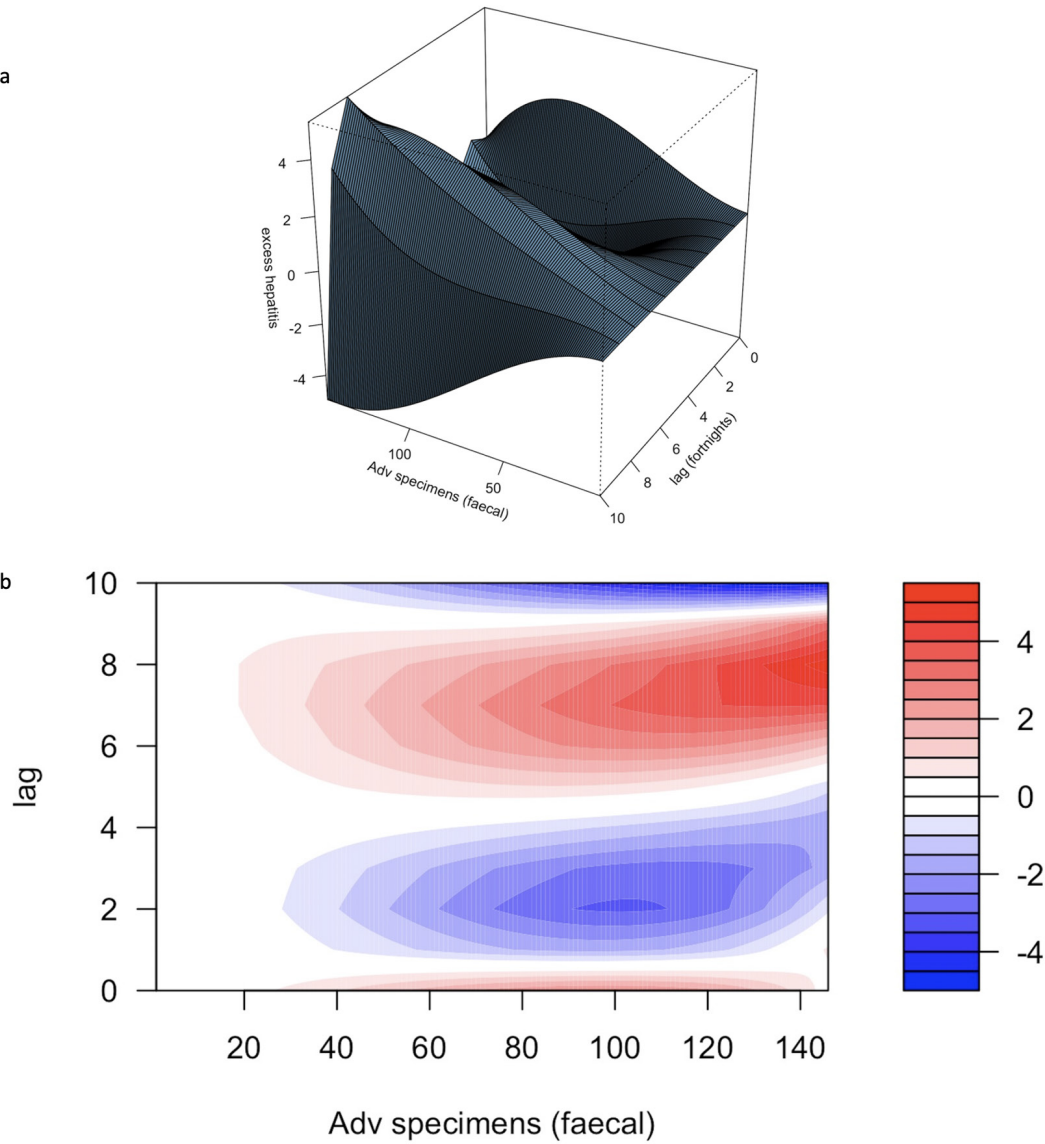
Association of excess hepatitis with faecal adenovirus positive specimens in 1–4 year olds in the most informative model. (a, b) The varying relationship between exposure (number of faecal specimens positive for Adv), and excess A&E attendances for liver conditions in 1–4 years at different time lags. (a) A three-dimensional plot and (b) a contour plot, where red represents the greatest positive excess, and blue the most negative excess. A positive relationship between number of positive faecal specimens and excess hepatitis appears to be seen at a lag of between 6 and 8 fortnights.

For HAdv faecal positive specimens (not corrected for testing or ascertainment) as an exposure, model 7 (modelling exposure outcome as a 3-degree polynomial and lags as a 4-degree polynomial) showed the lowest AIC (online supplemental table 5). The coefficients for first, second and third degree polynomial terms for the lags of the third-degree polynomial terms for the exposure were statistically significant (p<0.01). The overall association (reduced across the lag dimension) was non-monotonic (figure 3c,d and figure 4), showing a positive association between excess hepatitis and positive faecal specimens for adenovirus up to 35 positive samples, and a negative association up to 96 positive samples, followed by a positive association beyond this (figure 3d). Examining the association by lags, a positive association was observed at a lag of 14 weeks (figure 4 and online supplemental figure 4).

However, the best model for HAdv faecal samples had a higher AIC and MSE than SARS-CoV-2 positivity, indicating better fit (online supplemental table 2), and prediction of outcomes with SARS-CoV-2 positivity as an exposure compared with positive HAdv faecal specimen numbers over time. This can be observed in a visual representation of excess hepatitis plotted against positive adenovirus faecal samples with a lag of 7 periods of 2 weeks each (14–15 weeks) (figure 3c).

### Further evidence for strength of association and temporality criteria

These analyses suggest a statistically significant linear association between UK excess A&E attendances for children 1–4 years for liver conditions and SARS-CoV-2, with a lag of 16 weeks, as we outline subsequently. We found no association between respiratory HAdv positivity and excess A&E admissions in 1–4 years for liver conditions. When examining faecal positive specimens numbers (due to lack of availability of faecal positivity data), we find a potential association between positive specimens and excess A&E attendances for liver conditions, with a lag of 14 weeks. However, we note that the fit of the model and predictive value is much lower than for the model with SARS-CoV-2 positivity as the exposure (online supplemental table 5). The lag implies the association is only present with a delayed postviral illness rather than as a result of acute viral infection. The best-fitting model suggests a non-monotonic increase in excess hepatitis with an increase in faecal positive specimens.

Based on this additional analysis of data from England, the strength of association and temporality criteria for SARS-CoV-2 are fully met. For HAdv, there is little evidence of direct acute effect, and we assess that the strength and temporality criteria for HAdv are partially met. We find no temporal association with HAdv respiratory positivity, and the UKHSA does not report a consistent or clear increase in HAdv respiratory or faecal positivity over previous years. However, our exploratory analysis with faecal specimens positive for HAdv shows a lagged temporal association with excess hepatitis. The summary score for causality criteria were 11, 16 and 20 (of a total possible score of 27), respectively, for HAdv, AAV-2 and SARS-CoV-2, suggesting weak evidence for causality with HAdv alone, and moderate evidence for causality with SARS-CoV-2 and AAV-2 as exposures, especially SARS-CoV-2.

## Discussion

We examined the association between the 2022 epidemic of acute hepatitis of unknown aetiology in young children with SARS-CoV-2 and HAdv±AAV-2, which have been postulated as causative agents. Applying Bradford Hill criteria, our analysis suggests stronger evidence for SARS-CoV-2 than HAdv independently as a potential cause for acute hepatitis in children. We also find moderate evidence for AAV-2 as a potential causative agent (with HAdv, HHV-6 (Human Herpes Virus-6) and other viruses potentially being permissive viruses for infection). For SARS-CoV-2, there is evidence in the literature for temporality, strength of association, biological plausibility and analogy. Importantly, the plausible biological mechanisms include a postinfectious inflammatory pathology, possibly related to MIS-C or autoimmune hepatitis, as has been reported previously. Central to assessing SARS-CoV-2 as a possible cause of hepatitis is whether or not hepatitis (if related) is an acute or delayed complication. The temporality analysis is consistent with a delayed, postinfection hepatitis, consistent with higher seroprevalence in cases compared with controls reported in some studies (statistically significantly higher in one^16^ and not statistically significant in another).^17^ If this is a delayed presentation, it is likely an immunological mechanism rather than a direct viral mechanism, and so parameters that rely on detecting the virus or quantifying the patient’s viral load are not relevant. The temporal association is seen strongly on a population level when observing the pattern of the Omicron wave and the subsequent hepatitis wave, noting that the rate of Omicron infections was highest in children compared with other age groups,^14 18^ and substantially higher than previous waves. Therefore, a rare complication like hepatitis may have only become apparent during the first Omicron wave due to the much higher rate of infections in children.^19^ The peak in 5–17 years in England also occurred later than in adults during the Omicron wave, potentially reflecting school closure during December followed by accelerating spread after school opening in January.^18^ We note that there have not been continuing cases of hepatitis of unknown cause identified subsequent to the 2022 spring cluster. This may potentially be similar to the documented decline of MIS-C observed following the first omicron wave,^20^ given most children in several countries (the UK and the USA) were exposed by this point, and reinfections may be less likely to give rise to these postinfection inflammatory syndromes. We note that while the omicron wave was followed by identified paediatric hepatitis cases of unknown cause in many countries, this did not occur in all countries with large omicron waves (eg, Australia and New Zealand); we, therefore, consider the consistency criteria only partially met. Whether this represents lack of ascertainment due to gaps in surveillance, or statistical inability to pick up rare signals in smaller populations, or all causal conditions for hepatitis of unknown cause not being met in these regions is unclear.

For HAdv, the strength of association was specific to the UK and the USA but was not consistently observed in other countries—in fact, no association was observed in Canada, Japan or Europe once data from the UK was excluded. Where the association was observed, the viral loads of HAdV were consistently low in cases, and the virus was not recovered from hepatocytes in biopsies or explanted livers. This is inconsistent with invasive disease directly caused by the virus.^21^ There is also little comparative support for the other Bradford Hill criteria. Neither HAdv nor AAV-2 has been known to cause hepatitis in healthy children, and HAdv infection is not uncommon in children.^21^ This unusual manifestation would require one to posit either a novel strain of the virus or an additional novel mechanism (eg, altered host immunity) by which HAdv or AAV2 are able to cause hepatitis given that they have thus far not been known to cause this illness. Given no novel strain of the virus has been identified on sequencing, this may represent an incidental association due to reactivation of persistent viruses by immunosuppression or severe disease, or a permissive virus for AAV-2, or an interaction with SARS-CoV-2, with a SARS-CoV-2 superantigen response in a sensitised host.^22^ If faecal positive specimens are considered, a lag of 14–15 weeks provides the best fit, indicating if any causal effect exists, it would be a postviral effect, rather than a direct lytic effect, with HAdv observed in tissues is likely a result of persistence rather acute infection.

While we did not identify strong evidence of association between HAdv and hepatitis of unknown cause when examining Bradford Hill criteria across the studies reviewed, recent evidence suggests a possible causal association between AAV-2 and hepatitis of unknown cause.^16 23 24^ Small case–control studies within the UK have shown AAV-2 positivity among cases, including a presence in hepatocytes in liver tissue among those affected. We hypothesise that the association with HAdv may simply indicate a marker for AAV-2, given a permissive virus is required for AAV-2 infection. However, this still would not explain why AAV-2 would cause illness at this point when sequences do not suggest a novel strain, and this phenotype has not been observed before. We note that SARS-CoV-2 has been associated with immune dysregulation, including activation of latent viruses (eg, Epstein-Barr Virus^25^) and increased risk of infection several months post-COVID-19.^26 27^ It is plausible that past infection may activate latent viruses (like HAdv and HHV-6, and AAV-2), or that SARS-CoV-2 superantigens may sensitise the host to other viral infections (eg, AAV-2) and that hepatitis of unknown cause may reflect an interaction between the two agents.^28 29^ Another factor suggested that may have altered conditions are mitigation measures in 2020–2021 which may have led to concurrent surges of HAdv and AAV-2, leading to much higher levels of infection, with manifestation of a rare syndrome not identified before. However, we consider this unlikely given significant numbers of cases (12 for a population of 590 000 under 5s)^30^ were seen even in countries like Sweden, where mitigations were limited, and there was very little suppression of seasonal influenza, unlike in many other countries where this was considerably suppressed.^31^

The second part of our study provides some valuable additional information to support a likely temporal association of SARS-COV-2 infections with acute hepatitis in children. Using the UKHSA data, we find evidence of excess cases of paediatric hepatitis in 2021 and more strikingly in 2022 in England. Through the application of DLM, we find a significant temporal association between SARS-CoV-2 positivity rates and excess cases 16–17 weeks later between 26 April 2020 and 2 July 2022. We note that a previous study that reported no association, did not examine lags beyond 4 weeks, which would explain their null finding.^32^ The model estimated a 0.38 increase in excess cases at 16 weeks for a 1% increase in SARS-CoV-2 positivity in England. Over the same time period, no association was found between HAdv positivity and excess cases. We find a significant association between excess hepatitis and the number of HAdv positive faecal samples in England, consistent with a recent study,^32^ with a lag of 14 weeks; however, the fit of the model was poorer than with SARS-CoV-2 positivity. Furthermore, UKHSA data do not point to a clear increase in Adv faecal positivity across England, suggesting that case increases may potentially reflect an increase in testing. Our findings further support the strength of association and temporality of Bradford Hill criteria for SARS-CoV-2.

Further functional investigation, including examination of T-cell reactivity to pathogens within liver tissue and a clearer understanding of the underlying biology of this new syndrome, may help elucidate mechanisms and causality more clearly. It may be that the Omicron variants predispose more to a postinfectious hepatitis than previous variants in the presence of specific genetic predisposition, or alongside other infections (eg, AAV-2), or reinfections with omicron, or simply that the scale of infection was so much greater in 2022 that the epidemic of hepatitis was more noticeable. AAV-2 has not been known to cause disease before, but it is also possible that a unique combination predisposing factors (eg, coinfection, or genetic predisposition, or increased susceptibility post-COVID-19, or very high exposure) led to this rare manifestation in children. We also note that AAV-2 activation due to severe fulminant hepatitis cannot be ruled out, as there was a dearth of studies that included age-matched contemporaneous immunosuppressed controls, which would be needed to assess the role of immunosuppression in fulminant hepatitis in AAV-2 reactivation. In the only study that did include immunosuppressed controls, the age of controls was lower than cases, with the vast majority below 1 year,^16^ a group known to have the lowest seroprevalence of AAV-2, and thus likely to bias associations away from the null.

The lack of serological testing for SARS-CoV-2 in most available reports of hepatitis hindered the understanding of aetiology. Epidemiological surveillance, including serosurveillance for SARS-CoV-2 in children with hepatitis, remains key to outbreak investigation and early response in the future. Nonetheless, it is essential to acknowledge that relying on SARS-CoV-2 serosurveillance among children might not be entirely reliable or highly informative. This is because children, despite having similar viral loads and experiencing mild COVID-19, may be less prone to seroconversion compared with adults.^22^ Observational data on response of cases to antivirals and anti-inflammatory agents may also hold clues as to the causal factors for illness. Neutralising autoantibodies targeting the endogenous interleukin-1 receptor antagonist (IL-1RA) have been discovered in children with MIS-C and have recently been linked to the pathology of vaccine-induced myocarditis in adolescents.^33^ More research is required to understand if these or other autoantibodies may have a role in paediatric hepatitis of unknown cases, and what the antigenic trigger for these may be. Genetic association of class II HLA-DRB1*04:01 with hepatitis cases is consistent with the association of this allele with COVID-19 severity,^34^ indicating that a dysregulated immune response to the virus may be responsible for hepatitis observed in these cases. The HLA-DRB1 gene has also been implicated in an epigenetic study of MIS-C.^35^

There were limitations to this study. We reviewed current observational evidence and scored criteria for causality and the evidence supporting each to assess this within a causal framework. Given our reliance on limited observational data, we are unable to prove causality. Limitations of studies include biases in hepatitis case ascertainment, small number of studies, small sample sizes and inadequate matching where case–control analysis was done. In addition, lack of routine COVID-19 testing and the absence of complete serological data across countries was a limitation. Furthermore, as we did not have the data on individual EDs included in the study, we were unable to assess the representativeness of UKHSA data across the England population. We were also unable to examine the evidence for an interaction between the two exposures, that is, SARS-CoV-2 leading to an increase in susceptibility to infection by HAdv and/or AAV-2, as studies assessing both of these are currently very limited, and assessment of this criteria would likely require functional studies that currently do not exist. Additionally, our statistical modelling was limited by small sample sizes, the noisiness of routine A&E surveillance data, and the availability of aggregate rather than patient individually collected data. Given that we used excess A&E attendances and positivity rates, the findings are relatively robust to ascertainment of both outcomes and exposures. We have used data from England, which has reported the highest numbers of these cases for the statistical analysis. It is essential that cases of paediatric hepatitis of unknown aetiology have comprehensive investigation, including SARS-CoV-2 serology, which has not been reported or done systematically.

In summary, we demonstrate the use of the Bradford Hill criteria to evaluate the potential causal association of both SARS-CoV-2 and adenovirus with acute paediatric hepatitis of unknown aetiology. To support this qualitative analysis, we then employed statistical methods to assess the temporal association of SARS-CoV-2 and adenovirus separately with this syndrome. We find SARS-CoV-2 exposure to be more likely temporally associated with hepatitis as a potential post-COVID-19 autoinflammatory complication. More research is required to understand and prevent this potential complication of COVID-19. Vaccination is an available preventive measure which has been underused in some countries for children. Our study adds further support to the benefit of COVID-19 vaccination in children.

## supplementary material

10.1136/bmjpo-2023-002410 online supplemental file 1

10.1136/bmjpo-2023-002410 online supplemental table 1

## Data Availability

Data are available in a public, open access repository.
