# Supplementary material for: Acute hepatitis of unknown aetiology in children: evidence for and against causal relationships with SARS-CoV-2, HAdv and AAV2
Source: BMJ Paediatr Open. 2024 Dec 9;8(1):e002410. doi: 10.1136/bmjpo-2023-002410 (PMC11628968; doi:10.1136/bmjpo-2023-002410)
Supplement: online supplemental file 1 [file bmjpo-8-1-s001.pdf]

Supplementary Methods:

Statistical analysis

In order to examine the temporal correlation between the pre-specified exposures and outcome, we used distributed lag modelling.[31] This was applied to the period 26th April 2020 to 2nd July 2022 for all exposures assessed, as SARS-CoV-2 positivity data was only available from 26th April 2020. Although adenovirus positivity and adenovirus faecal specimen positive data was available prior to this date, we limited our analysis to this time period to ensure models were comparable in terms of data points, and estimated AICs, and had the same power to detect an effect.

Distributed lag models (DLMs) allow regression of one time series (dependent variable) on another (independent variable), estimating the distribution of lags between them, as these are considered unknown. These lags can be constrained to fit a polynomial distribution, or allowed to vary, but with a finite cap in time.

The DLM models the current and delayed effects of an independent [32] series on a dependent [33] series. The infinite linear DLM is written as follows[31]:

$$Y_t = \alpha + \sum_{s=0}^{\infty} \beta_s X_{t-s} + \epsilon_t$$

Where α is the intercept (representing the mean effect) and the β_s coefficients represent the lag weights for each time point going backwards from the current time. The parameter ϵ_t is the error term for the time point *t* with zero mean and constant variance. So for every time point *t*, Y_t depends on a weighted sum of X at previous time points up to the current time, with weights being estimated for time points s=0,1,2…∞. In practice, however Y_t is unlikely to depend on X beyond a certain time point i.e. any effect of X on Y_t is likely to be minimal beyond this time point, say *q*. Given this, we can cap the model at time point q, so that a finite DLM linear model is written as:

$$Y_t = \alpha + \sum_{s=0}^{q} \beta_s X_{t-s} + \epsilon_t \qquad (1)$$

This assumes that beyond time point q the lag weights would be zero, or near zero, and limits the number of parameters to be estimated by the model.

Given that a large number of parameters would need to be estimated with n-q $\{X_t, Y_t\}$ pairs available for estimation, we included polynomial models, where the lag weight β_s follows a distribution represented by a polynomial function of time. For example, a quadratic lag distribution model can be written as:

$$\beta_s = \xi_0 + \xi_1 s + \xi_2 s^2, s = 0, 1, \dots, q, \qquad (2)$$

Where ξ_0, ξ_1 and ξ_2 are the parameters of the quadratic function describing the lag weights.

Substituting into equation 1, we get:

$$Y_t = \alpha + \sum_{s=0}^{q} (\xi_0 + \xi_1 s + \xi_2 s^2)\, X_{t-s} + \epsilon_t$$
$$= \alpha + \xi_0 \sum_{s=0}^{q} X_{t-s} + \xi_1 \sum_{s=0}^{q} s X_{t-s} + \xi_2 \sum_{s=0}^{q} s^2 X_{t-s} + \epsilon_t$$

This can be rewritten as:

$$Y_t = \alpha + \xi_0 z_t^0 + \xi_1 z_t^1 + \xi_2 z_t^2 + \epsilon_t, \quad (3)$$

Where $z_t^0 = \sum_{s=0}^{q} X_{t-s}$, $z_t^1 = \sum_{s=0}^{q} s X_{t-s}$, $z_t^2 = \sum_{s=0}^{q} s^2 X_{t-s}$,

The z variables can be constructed by simple transformations of X using a chosen value of q, which allows estimation of the equation by standard linear methods. Once the parameters are estimated, the lag weights β_s can be derived from these based on equation 2. While this is the quadratic formulation, this model can be generalized to cubic or higher-order polynomials as well. The higher the order of the polynomial, the less “smooth” the lag distribution is allowed to be, and the more parameters need estimation.

For our analysis, given the best distribution is unknown, for both exposures, we considered a number of pre-defined models (**Supplementary Table 1**): a finite distribution lag model, polynomial models with orders 2, 3, and 4, as well as more flexible distributed lag non-linear models (DLNMs), which allows for non-linear relationships between both the exposure and outcome, and between lag and outcome. These DLNMs bidimensional set of coefficients for the exposure were reduced across the lag dimension into the overall cumulative exposure-response curve between fortnightly average exposure and excess hepatitis.

For all models, we included a seasonal effect by including weeks modelled with a natural cubic spline with 3 internal knots (13, 26 and 39 weeks) for each year. We chose the best model by comparing the Akaike information criterion (AIC). We also examined the mean squared error between observed and predicted values across all models for comparison. For our cap on time effects, we considered 10 fortnight periods (20 weeks) as the maximum lag for effect (q), and weights beyond this lag were implicitly considered zero.

To assess whether the exposures contributed statistically significantly to the model, for linear exposure functions, we assessed significance of individual coefficients by the Wald test (a test of the null $\beta_s = 0$). Given estimation of multiple parameters in these models (multiple testing), we considered a pre-specified value of 2-tailed p<0.01 as statistically significant. For DLNMs we considered the coefficients along the overall exposure-response

curve reduced along the lag dimension. Analyses were conducted in the DLagM and DLNM packages in R.
