# Supplementary material for: Acute hepatitis of unknown aetiology in children: evidence for and against causal relationships with SARS-CoV-2, HAdv and AAV2
Source: BMJ Paediatr Open. 2024 Dec 9;8(1):e002410. doi: 10.1136/bmjpo-2023-002410 (PMC11628968; doi:10.1136/bmjpo-2023-002410)
Supplement: online supplemental table 1 [file bmjpo-8-1-s002.pdf]

Supplementary Table 1: DLNMs and DLM models used to assess the relationship between exposure and outcome and lag and outcome

DLNM/DLM model	Exposure-outcome	Lag-outcome
Model 1	linear	unconstrained
Model 2	Polynomial degree=2	unconstrained
Model 3	linear	Polynomial degree=2
Model 4	linear	Polynomial degree=3
Model 5	linear	Polynomial degree=4
Model 6	Polynomial degree=2	Polynomial degree=4
Model 7	Polynomial degree=3	Polynomial degree=4
Model 8	Polynomial degree=4	Polynomial degree=4
Model 9	linear	Natural cubic spline df=4
Model 10	Polynomial degree=2	Natural cubic spline df=4
Model 11	Polynomial degree=3	Natural cubic spline df=4
Model 12	Polynomial degree=4	Natural cubic spline df=4
Model 13	Natural cubic spline df=4	Natural cubic spline df=4

Df: degrees of freedom

Supplementary Table 2: Bradford criteria for SARS-CoV-2 as a causal agent for paediatric hepatitis of unknown cause

Criteria	Summary of evidence	Limitations	Inference
Strength	Acute COVID-19 and hepatitis<br>• Odds of developing acute hepatitis 3.1x greater in patients with COVID-19 compared with influenza in a retrospective cohort study[2]<br>• Patients hospitalised with COVID were more likely to have elevated liver enzymes compared to controls admitted for other respiratory infections in case-control studies[3 4]<br>• Case-control study of hepatitis cases admitted through A&E and matched controls showed no statistical differences in SARS-CoV-2 positivity at admission or 2 weeks prior to admission in cases and controls[1]<br>• SARS-CoV-2 PCR positivity among paediatric hepatitis cases ranged from 9-20% across different studies, so the vast majority of cases were not positive for SARS-CoV-2 at presentation[1]<br>• Evidence of viral inclusions in some but not all studies of patients with COVID-19 associated hepatitis [5-7]<br>• Several case series of acute hepatitis in children infected with SARS-CoV-2[8-13]	Limited research on the association between acute infection and hepatitis, as well as between post-acute complications of COVID-19 manifesting as hepatitis.	Although there is an association between COVID-19 and acute hepatitis, the majority of cases of paediatric fulminant hepatitis of unknown cause were not SARS-CoV-2 positive at the point of admission. Criterion of strength minimally met for association between acute COVID-19 and hepatitis of unknown cause.
	Hepatitis as a Post-COVID complication<br>• Acute hepatic failure and liver involvement has been described as a post-COVID complication and is recognized in MIS-C [14-16]<br>• Elevated alanine aminotransferase (ALT) was 2.3x more common in children and young people who develop MIS-C after COVID-19 than in patients with acute COVID-19 infection, with the majority having no underlying medical conditions.[15] Elevated liver enzymes were 2.3x more commonly seen in children compared to other respiratory infections 1,3, and 6 months following COVID-19 in an electronic health record study of children in the US under 10.[17]	Although almost all cases of hepatitis from countries such as Israel which had good ascertainment of COVID infection in children had previous SARS-CoV-2 infection, the time delay between positivity and hepatitis was not reported for	The criterion of strength is partially met for SARS-CoV-2 with hepatitis as a post-infectious syndrome. Additionally considering modelling of UK SARS-CoV-2

	• Four out of five children with acute hepatitis of unknown cause from Netherlands had past exposure to SARS-CoV-2 or active infection at the time of admission[18] Previous infection, when date was known varied from 4-10 weeks prior to presentation.<br>• Children under 10 with COVID-19 had 2.5 fold increased risk of elevated liver enzymes post-COVID compared with children with other respiratory infections.[17]<br>• Hepatitis followed the peak of the delta wave in India occurring 2-6 weeks after asymptomatic or mild symptomatic infection, or following exposure to a lab-confirmed case of COVID-19.[19]<br>• In England, 50% of children were exposed to SARS-CoV-2 infection over a few months during the omicron wave,[20] the peak of which in children (late January) preceded the cluster of hepatitis cases identified by 2-4 months<br>• Eleven of 12 hepatitis cases of unknown cause reported from Israel (where case ascertainment in young children has been relatively good) were found to have previously recovered from SARS-CoV-2.<br>• There is a case report of paediatric acute fulminant hepatitis post-COVID from the USA (occurring 4 months following infection) with liver biopsies suggestive of auto-inflammatory necrosis.[21]<br>• Multiple studies of paediatric hepatitis cases have reported high seropositivity for SARS-CoV-2 to date (~60-80%),[1 22-25] suggesting very high exposure in this group given known low seroconversion and high seroreversion rates in children.[26 27]<br>• The a UKHSA study comparing SARS-CoV-2 seropositivity in 1-4 year olds cases with hepatitis attending A&E compared with age-matched controls showed that the difference was not statistically significant between these (60.5% and 46.3% in cases and controls, respectively).[1]	all cases, and appears to have been variable where reported[30] Although seropositivity was high in hepatitis cases, background exposure was also high in the population, making the significance of this in cases unclear. The UKHSA study[1] comparing seropositivity among cases and controls showed non-significant higher seropositivity in cases, but was underpowered to detect a difference. Another small study showed higher seropositivity in cases than controls, with small sample sizes.[28]	data against excess hepatitis presentation to A&E, we assess that strength criteria is fully met.

	• In another study comparing seropositivity of cases and controls, seropositivity was higher in cases (15 of 20) than in controls (3 of 10)[28]<br>• A study from Scotland 12 out of 23 (52%) of the children with were seropositive for SARS-CoV-2 compared to an estimated background seroprevalence of between 59-72% between March-June '22.[29]		
Consistency	<u>Acute COVID-19 and hepatitis</u><br>• Most cases in the current outbreak did not test positive for SARS-CoV-2, with only ~10% of probable cases positive for SARS-CoV-2 by PCR or rapid antigen test across multiple studies [31 32]<br><u>Hepatitis as a Post-COVID complication</u><br>• Although serological testing was not carried out in most cases of hepatitis, [33] reports from many different countries among samples tested show SARS-CoV-2 seropositivity consistently between 60-80%[1 22-25]<br>• These seropositivity levels are consistent with those seen in in PIMS-TS, a known post-COVID complication that is also associated with hepatitis.<br>• The clusters of cases of unknown hepatitis peaked in the US, UK, Spain, Italy, the Netherlands and Poland in April-May 2022, ~2-3 months following the first omicron wave in these areas. [31 34 35]<br>• However, we also note that significant clusters of hepatitis cases were not observed in some regions despite high levels of omicron infection (e.g. Australia, New Zealand, and South Korea). This could be because cases did not consistently occur post-COVID (SARS-CoV-2 not being a sufficient condition to be causal), or poor background surveillance, genetic predisposition or rarity of the outcomes (hence a signal was not seen in countries with small populations).	If SARS-CoV-2 exposure were incidental and not causal, one may expect seropositivity among cases to differ across countries, given exposure of children to infection is likely to have been different given different levels of mitigations and exposure over time in different countries. However, this was not available in many cases. Clusters are difficult to compare across countries due to differential surveillance and case detection.	Criterion of consistency is partially met for SARS-CoV-2 and post-infectious hepatitis.
Specificity	<u>Acute SARS-CoV-2 and post-COVID associated hepatitis</u>		

	• At the peak of the Omicron wave in children, SARS-CoV-2 prevalence was >10% of children 5-17 years in England, being >2x higher than in adults, and higher than any other point previously during the pandemic.[36] This period saw unprecedented exposure of children in England; 50% of children were infected rapidly during the first 4 months of the omicron wave.[37]<br>• 12 out of 23 (52%) of the children with hepatitis were seropositive for SARS-CoV-2; this was not higher seroprevalence in children aged 5–11 years in Scotland between 14 March and 27 June 2022, reported as being between 59-72%.[29]<br>• The a UKHSA study comparing SARS-CoV-2 seropositivity in 1-4 year olds cases with hepatitis attending A&E compared with age-matched controls showed that the difference was not statistically significant between these (60.5% and 46.3% in cases and controls, respectively).[1]<br>• In another study comparing seropositivity of cases and controls, seropositivity was higher in cases (15 of 20) than in controls (3 of 10)[28]<br>• SARS-CoV-2 was not the only exposure identified in cases. HAdv, AAV-2, HHV-6 and EBV were also over-represented in some case series (see Tables 4 and 5)<br>• Three out of five cases in a case series from Netherlands were positive for hAdv (at very low levels), with another positive post-admission. 4 of 5 children in the same series had current or past infection with SARS-CoV-2, one was positive for enterovirus, 1 for VZV, and 1 for EBV.[18]	The UKHSA study[1] comparing seropositivity among cases and controls showed non-significant higher seropositivity in cases, but was underpowered to detect a difference. Other viruses such as AAV-2, hAdV, HHV-6 were also identified in case series.	Criterion of specificity is only minimally met for SARS-CoV-2 and hepatitis as a post-COVID complication
Temporality	• The peak of the omicron wave in children in England (late January/early February) preceded the peak of hepatitis cases (May) by 3-4 months, consistent with a post-COVID auto-inflammatory complication similar to MIS-C [38]	Apart from limited reports from Israel,[16] where data on SARS-CoV-2 positive tests in children was available, in most	Temporality criterion is partially met

	• A similar increases in hepatitis was also reported in India following the delta wave.[19]<br>• The clusters of cases of unknown hepatitis peaked in the US, UK, Spain, Italy, the Netherlands and Poland in April-May 2022, ~2-3 months following the first omicron wave in these areas. [31 34 35]<br>• There is a case report of paediatric acute fulminant hepatitis post-COVID from the USA (occurring 4 months following infection) with liver biopsies suggestive of auto-inflammatory necrosis.[21]<br>• Cases in Indonesia were identified in reports from May-September in the period following the omicron wave that peaked in late February.<br>• Hepatitis cases and MIS-C have both shown reduction over time in multiple countries. This may reflect an increase in re-infections observed in children (which may provide a level of protection from severe disease), or a specific relationship to the first Omicron variant.[39]<br>• High seropositivity among cases establishes temporal precedence of SARS-CoV-2 among cases, although children do not sero-convert as reliably as adults.<br>• A case series of 5 children in Israel showed SARS-CoV-2 infection preceding hepatitis by between 3 weeks-4 months in cases, where infection had been previously diagnosed.[16]<br>• We note that clusters of hepatitis cases were not observed in some regions despite high levels of omicron infection (e.g. Australia, New Zealand, and South Korea) 2-3 months following infection – however, a rare post-COVID signal would likely be apparent only in large populations, and regular surveillance of hepatitis of unknown cause, given these constitute ~40% of cases of all acute liver failure in children.	countries, previous infection in children was not ascertained. Although seropositivity suggests very high previous exposure, it does not identify when this exposure happened with respect to hepatitis.	

Biological gradient	<u>Acute COVID-19 and hepatitis</u><br>• A case series of children hospitalized for COVID-19 found that elevated liver enzymes were more prevalent among those admitted to ICU, suggesting that increased disease severity may be related to liver involvement. [15]<br>• Studies in adults have noted that increased severity of COVID-19 disease is associated with increased risk of liver abnormalities. [40-48]<br>• A dose-response study in ferrets showed that portal inflammation of the liver was more severe in ferrets that were inoculated with a high or medium dose of SARS-CoV-2 compared to those inoculated with low or control dose. [49]	The relationship between viral load, and severity of acute illness and hepatitis is unknown among cases as infection was diagnosed retrospectively through serological testing in most cases	Criterion of dose-response for SARS-CoV-2 exposure and hepatitis only minimally met
Plausibility	<u>Acute COVID-19 and hepatitis</u><br>• There are numerous possible biological explanations for the association between COVID-19 infection and hepatitis [3 44 50-57]<br>• There have been multiple case reports of children developing severe acute hepatitis following COVID-19 infection [8 16 58-62], including after mild COVID-19 symptoms [8 62], or after recovering from COVID-19 [8 16]<br>• Liver abnormalities appear to be a common complication of COVID-19 infection in adults[43]<br>• There is some evidence of direct infection of the liver by SARS-CoV-2 which uses the angiotensin-converting enzyme 2 (ACE2) cell receptor expressed in liver tissue to invade human cells.[63] Some studies, but not all, found evidence of viral inclusions in patients with COVID-19 associated hepatitis [5-7]<br><u>Hepatitis as a Post-COVID complication</u><br>• Severe hepatitis is a frequent manifestation of MIS-C, a post-viral syndrome following infection with SARS-CoV-2 [15 61 64-67]		The criterion for plausibility of association between SARS-CoV-2 and hepatitis is fully met

	• SARS-CoV-2 positivity by PCR and serology in the hepatitis cluster are consistent with studies on children with MIS-C,[64] thus the current cases of hepatitis could be occurring via a similar mechanism.<br>• Acute paediatric auto-immune hepatitis has been observed with SARS-CoV-2.[14]<br>• One study found hepatitis cases had 12-fold greater odds of carrying the HLA-DRB1*04:01 allele [68]. This allele is associated with autoimmune conditions[69] and consistently associated with milder or asymptomatic infection with SARS-CoV-2 compared to controls [70-72] This allele has recently been shown to enhance the effect of HLA-B*15:01, which is associated with asymptomaticity following COVID infection, and a cross-reactive T cell response to SARS-CoV-2, providing a potential mechanism for this association.[73] It is plausible that this allele or the haplotype it is present on is involved in a potentiated immune response to SARS-CoV-2, which may also explain its enrichment in case series of hepatitis, if SARS-CoV-2 infection is on the causal pathway<br>• SARS-CoV-2 has been associated with immune dysregulation, including activation of latent viruses (e.g. EBV[74 75]). It is plausible that past infection may activate latent viruses (like HAdv and HHV-6, and AAV-2). It has also been associated with increased risk of infection several months post-COVID, including streptococcal tonsillitis[76] and RSV infection.[77] It is plausible that it may have increased the risk of infection with other permissive viruses leading to AAV-2 infection/activation.<br>• Recent analysis of clonally expanded T cell receptors among patients following COVID-19 infection showing targeting of self-antigens that are abundantly expressed within liver cells		

	suggesting a potential auto-immune basis to hepatitis of unknown cause.[78]<br>• CD8-T cell dominant auto-immune hepatitis has been observed 2-3 weeks post mRNA vaccination for COVID-19.[79 80]		
Coherence	• See also biological plausibility<br>• The occurrence of hepatitis are coherent with existing knowledge of post-COVID syndromes such as MIS-C. [24 68]<br>• Re-activation of persistent/latent viruses, as observed in cases of hepatitis would also be coherent with the known pathophysiology of COVID-19<br>• Hepatitis is a common manifestation of MIS-C, occurring in approximately 50% of cases [15 61 65-67]. MIS-C cases often test negative to COVID-19 via PCR but are positive by serology [64] although this can be influenced by low rates of seroconversion in children[81]<br>• Acute paediatric auto-immune hepatitis has been observed with SARS-CoV-2.[14]		Coherence criterion for COVID-19 and hepatitis as a post-COVID complication is fully met
Experimental Manipulation	• Allocation of alleles can be considered as random, as in mendelian randomisation studies. Class II HLA-DRB1*04:01 identified as strongly associated with hepatitis of unknown cause in a small sample has also been associated with COVID-19 severity in other studies, suggesting a potential link between mechanisms that are associated with COVID-19 severity and acute hepatitis of unknown aetiology.<br>• A study in Chinese tree shrews found that SARS-CoV-2 inoculation was associated with marked increases in aspartate transaminase [82]<br>• Another study in ferrets found that inoculation with high doses of SARS-CoV-2 was associated with signs of hepatic lobular inflammation [49].	There are no trials of experimental treatments for hepatitis. Limited case series suggest response to steroids,[16] which suggests this may be an auto-inflammatory phenomenon, but in the absence of trials, inferences cannot be made.	This criterion is only minimally met

Analogy	• The current hepatitis presentation is analogous to MIS-C, a pediatric multisystem inflammation syndrome that is suspected to be linked to COVID-19 [64]<br>• Hepatitis is a known complication of MIS-C, occurring in nearly half of cases [15 61 64-67].<br>• Liver biopsies and explanted liver histology consistently showed evidence suggestive of auto-inflammation consistent with what has been observed in MIS-C. No evidence of acute viral infection was observed.<br>• CD8+-dominant auto-immune hepatitis has been observed in response to COVID-19 mRNA vaccination[79]<br>• The majority of MIS-C cases do not test positive for COVID-19 via PCR, but diagnosis is often confirmed via serology, suggesting it is a post-infectious syndrome [64]. This could explain why a majority of cases in the current outbreak have not tested positive for SARS-CoV-2 [24 33]<br>• there are some individual reports of hepatitis associated with infection with the original SARS and MERS-CoV [83 84], but no larger studies to examine this association<br>• Acute paediatric auto-immune hepatitis has been observed with SARS-CoV-2.[14]		We conclude that this criterion is fully met

Supplementary Table 3: Bradford criteria for Adenovirus as a causal agent for paediatric hepatitis of unknown cause

Criteria	Evidence	Limitations	Inference
Strength	• A recent study examining retrospective liver samples from a cohort of paediatric acute liver failure of unknown cause from 2007-2014 in the US did not find hAdv in any of the 44 liver biopsy samples examined or 19 serum samples examined, showing this has not been historically associated with acute liver failure in children. [85] Three of 35 were positive for CMV, 2 for EBV, 2 for HSV and 1 for HHV-6.<br>• Adenovirus positivity data during 2017 to 2022 from Manchester Foundation Trust/UKHSA did not show any clear trend for blood, faecal or respiratory samples, although positive specimens showed an increase, likely due to better ascertainment of cases.[23]<br>• A study in Ireland showed an increase in wastewater detection of AAV-2 and hAdV-F concurrent with the period of detection of cases of hepatitis of unknown cause[86] No correlation was observed with SARS-CoV-2 levels in wastewater at the same time; however, lagged correlation to assess hepatitis as a post-COVID complication was not conducted in this study.<br>• A study of cases showed very low levels of HAdV DNA, no proteins, inclusions or viral particles, including in explanted liver tissue from affected cases[29]<br>• A study in Scotland showed detection of hAdV in 3 out of 9 cases of hepatitis and 0 out of 13 healthy controls (non-significant). HHV-6 was detected in 3 out of 4 cases of hepatitis compared with 0 out of 13 healthy controls[29]	• Case-control studies for HAdv have been limited by controls being sampled during periods of lower HAdv circulation,[29] potentially significantly limiting conclusions around causality, given confounding by changes in circulation over time, as described previously.[29]<br>• HAdv and HHV-6 positivity has been inconsistently higher among cases than controls across different studies with strength of association varying widely.<br>• Viral loads have been consistently low for HAdv among cases in all studies to date<br>• The strong Europe-wide association of HAdv with severe hepatitis is likely to be at least partly driven by confounding by country, as the UK had both the highest proportion of HAdv infection, and the highest proportion of cases admitted to ICU, as outlined.[32]	The strength criteria for an association of persistent or activated HAdv/HHV-6 and with hepatitis is partially met, due to the strength of the association varying widely from the null to strongly associated across studies, and consistently low viral load among cases, across all studies where this was examined.

	• HAdV and HHV6B read counts were very low, and Ct levels were not different in cases and controls in plasma or liver samples in 28 cases and 74 controls[29]<br>• Contemperaneous hospitalized samples taken during the same period of hepatitis cases showed hAdv was circulating at low levels in children during March and April 2022[29]<br>• A case-control study in the United Kingdom found that suspected cases had 35-fold higher odds of detectable hAdV compared to age, time and region matched controls admitted to hospital for acute illness (non-hepatitis). [23 87]<br>• In a case series, seven of eight patients in England who required liver transplantation tested HAdV positive in blood samples, with F41 found in five of five genotyped.[88]<br>• Across the WHO European region, cases with a positive hAdV result were statistically significantly more likely to be admitted to ICU/HDU (OR = 2.11; 95% CI: 1.18–3.74) and to receive a transplant (OR = 3.36; 95% CI: 1.19–9.55) than cases without infection.[32]<br>• A US study showed hAdV detected in all 14 cases, of which 11 (79%) were genotyped as HAdV-41, compared to 8% (9 of 113) detection of HAdV-41 among controls.[8930]<br>• There was no association between adenovirus and hepatitis in Japan, with only 9% of cases positive.[33]<br>• Only 6 of 26 patients with paediatric unknown hepatitis in a case-series from Italy were positive for hAdv.[90]<br>• To date no acute or post-viral illness causing acute fulminant hepatitis with HAdv and/or AAV-2 has been described in immunocompetent children.<br>• In children, the tonsils, adenoids, and gut mucosa are common reservoirs for persistent HAdv[91] which can persist in	• Detection of HAdv in blood, stool, or respiratory samples through PCR is not necessarily indicative of a disease *caused by* HAdv and may be due to persistence and/or activation. This has also been observed in cases of Kawasaki disease where testing for HAdv led to a seemingly strong association, when it was a bystander.[94]<br>• The lack of appropriate disease, time and severity matched controls[29 93] means that viral re-activation as an incidental association cannot be ruled out in this case for hAdv. Children admitted to ICU with elevated liver enzymes (but without fulminant hepatitis) are not adequately matched, as fulminant hepatitis is a state of immunodeficiency, in a way that hepatitis alone is not.	

	immunocompetent children after initial infection for months to year, and disseminate into the blood in states of immunosuppression, such as acute liver failure.[91]<br>• Plasma levels of HAdv, which have previously been shown to be high in invasive disease[92] have been very low in cases (to the point that full typing and sequencing of the virus has been challenging)[29 93]<br>• Detection has been more reliable in blood than in plasma in cases of hepatitis, as per UKHSA reports,[1] more consistent with HAdv persistence in lymphocytes (in blood) rather than acute invasive disease.<br>• One study required enrichment sequencing for detection in the majority of samples, as PCR showed much lower sensitivity, consistent with very low virus levels in plasma.<br>• Three out of five cases in a case series from Netherlands were positive for hAdv (at very low levels), with another positive post-admission. 4 of 5 children in the same series had current or past infection with SARS-CoV-2, one was positive for enterovirus, 1 for VZV, and 1 for EBV.[18]		
Consistency	• The frequency of adenovirus detection in cases has varied markedly across regions, type (where available) and tissues sampled.[33]<br>• Studies of cases of hepatitis compared to contemporaneous healthy controls, or non-age matched immunocompromised controls in the UK and US have shown inconsistent associations between hAdv and cases.<br>• In the UK, 172 of 252 cases were positive in at least one sample for hAdv (68%)[87]	There is inconsistency of HAdv positivity across different case series. European data suggests that adenovirus may be incidentally implicated due to increase in cases in the UK during the time period when hepatitis cases emerged, which may explain why frequency in cases was much lower in other countries.[32] Another possibility is	This criterion is partially met for the association between hAdv and hepatitis. The association seems to be primarily driven by studies from the UK, and frequency observed in cases appears to be lower in several

	• Of 144 suspected cases in other European countries, only 35% of 106 cases with adenovirus testing data available were positive.[32]<br>• In the US, of 252 cases tested for HAdv and 45% were positive [31]<br>• in Japan, only 9% of cases tested (58 out of 67) were positive. [33]<br>• Only 6 of 26 patients with paediatric unknown hepatitis in a case-series from Italy were positive for hAdv.[90]<br>• As per the ECDC and WHO European surveillance, 218 out of 404 (54%) cases tested positive for HAdv on any sample.[35] When adjusting by age, cases in the UK were still significantly more likely to be HAdv positive than cases reported from other European countries (OR = 3.10; 95% CI: 1.90–5.07).[32]<br>• Data from Europe suggests that the association between HAdv and unknown aetiology hepatitis is driven largely by data from the UK, with positivity being relatively low when these data are removed.[32]<br>• Among HAdv subtypes, HAdv 41F has been the most frequently identified active infection (92%) among suspected cases in the UK hepatitis cluster.[1]<br>• The majority of samples in other countries were not subtyped but amongst the European samples that were, 50% (4 out of 8) were HAdv 41, with the remaining being other subtypes.<br>• Metagenomics analysis in the UK suggested that HAdv41F did not belong to a single clade and was representative of background diversity in the UK.[1]<br>• All studies to date have found very low viral load of hAdv in cases, when hAdv was present, with hAdv not observed in hepatocytes in explanted livers or biopsies.	that the cause of hepatitis is different in different countries (given the excess in A&E attendances for liver conditions in children observed in the UK, which is not as clearly seen in some other countries[31]); however, we consider this less likely.	other countries, including in Europe and Japan.
Specificity	• Between 20-40% of cases also tested positive for known latent viruses like EBV, HHV-6 and HHV-7,[1] likely to indicate reactivation	Multiple viruses were recovered both in liver tissue and other samples among cases, and controls,	There is a lack of specificity of permissive viruses such as hAdV, EBV

	• Of five explanted livers from the UK, metagenomics identified high levels of AAV-2 and low levels of HAdv, and HHV-6B[1 28] (all persistent viruses) in all livers, indicating possible re-activation of virus due to severe fulminant hepatitis, or AAV2 as a causal agent alongside a permissive virus.<br>• AAV-2 PCR was positive in 9/9 formalin-fixed paraffin-embedded (FFPE) liver samples, HHV-6B PCR in 6/9 samples and HAdV in 4/9 samples.[28]<br>• In the UK, 172 of 252 cases were positive in at least one sample for hAdv (68%). 31% were positive for HHV-6, 64% seropositive for SARS-CoV-2 [87]<br>• 27 of 28 cases tested were AAV2 PCR positive (high levels), 23 of 31 were HAdV positive and 16 of 23 were HHV-6B positive (low levels). [28] AAV-2 was also detected in younger immunosuppressed controls but at lower frequency (11/32) as was HHV-6B (5/15).<br>• HHV-6 was also overrepresented in cases in a small study[93] which may point to re-activation of persistent viruses in the relative immuondeficient state of severe fulminant hepatitis, or presence of a virus permissive to AAV2 replication.[29]<br>• Co-infections by EBV, HHV-6 and/or enterovirus A71 were also detected in 12 (85.7%) of 14 cases, with higher herpesvirus detection in cases versus controls ($P < 0.001$). The association of HAdv was not tested as cases were selected based on HAdv positivity<br>• While HAdv41F has been the dominant subtype of HAdv identified, other subtypes have also been identified in the UK, and in other countries.<br>• Cases with hepatitis of unknown aetiology contained AAV2 RNA in nuclei and/or cytoplasm of ballooned hepatocytes as well as in	including HHV-6, HAdV and AAV-2. This suggests the possibility that at least some of these may represent activation of latent viruses in a critically ill host. AAV-2 was most frequent in samples and enriched in cases compared to controls, where examined. However, studies that examined this were limited, and sample sizes were small. hAdV was not specific to cases.	and HHV-6 overrepresented among cases (at low levels), as well as AAV-2 (at high levels). We therefore deem this criteria minimally met for hAdV.

	arterial endothelial cells. The levels of AAV2-positive cells ranged from 1.2% to 4.7% in these biopsies.[29]<br>• Three out of five cases in a case series from Netherlands were positive for hAdv (at very low levels), with another positive post-admission. 4 of 5 children in the same series had current or past infection with SARS-CoV-2, one was positive for enterovirus, 1 for VZV, and 1 for EBV.[18]<br>• A case series from Italy identified multiple viruses across specimens including cytomegalovirus, Epstein-Barr virus, Leishmania, E. Coli, HHV6 and 7, Norovirus, Rotavirus, SARS-CoV-2, Rhinovirus, Parvovirus, Metapneumovirus, Paraechovirus, Enterovirus, Influenza A, Coronavirus OC43, Salmonella paratyphi, C. Difficile, and S. Pneumoniae. HAdv was identified in only 6 of 26 (23%) samples.[90]		
Temporality	• The respiratory adenovirus positivity rate does not appear to have increased in the UK in under 5 year olds, other than an increase in children under five observed in Wales between January and May 2022 that subsequently decreased to pre-pandemic levels [23].<br>• While data on gastroenteritic adenovirus positivity is not directly reported by UKHSA, we note that the UKHSA did not find a consistent increase in this over previous years in sampling from different regions<br>• Increases in HAdv positive specimens may have been due to increased testing in England rather than increases in infection level beyond that of previous years, as there is no clear increase in positivity as per UKHSA reporting	We were unable to identify any evidence directly in support of a temporal association between adenovirus and acute hepatitis. The presence of AAV-2 on admission may be indicative of a recent adenovirus infection or reactivation which may have preceded hepatitis, or followed it due to fulminant hepatitis being an immunosuppressed state.	We consider this criterion partially met for hAdV

	• Similarly, a study found that since October 2021, the percentage of tests positive for adenovirus types 40/41 among children in the US has not increased relative to pre-pandemic levels [95]<br>• A study in Ireland showed an increase in wastewater detection of AAV-2 and hAdV-F concurrent with the period of detection of cases of hepatitis of unknown cause[86]<br>• Given most cases were tested at the point of admission for hepatitis, a positive test for HAdv or AAV-2 can be considered as evidence of being infected at the time of presentation.[96]<br>• It is important to delineate acute infection from re-activation of virus and the temporal association of these with hepatitis. As fulminant hepatitis in itself is considered a state of immunosuppression, and most cases were in this state at the point of testing, it is difficult to determine whether HAdv preceded hepatitis, or followed it.<br>• A case-control study in Scotland found that 100% of nine suspected cases were positive for AAV2, which first requires infection with a 'helper' virus such as hAdv. Many of these cases had a history of gastrointestinal illness several weeks before the onset of hepatitis symptoms. This suggests that hepatitis may have been preceded by hAdv infection or reactivation[68].<br>• The 12 transplanted cases reported gastrointestinal symptoms (nausea, vomiting and diarrhoea) preceding transplant by a median of 20 days (range 8–42 days)[28]		
Biological gradient	• HAdv levels recovered from cases have been consistently low, with sequencing being challenging. However, levels have been higher in cases than in non-age-matched immunocompromised controls in one study of 14 cases and 14 controls, suggesting a possible gradient.[28]	Due to potentially inadequate matching for age in immunosuppressed controls these findings must be interpreted with caution, and could represent viral reactivation.	This criterion is minimally met for HAdv

Plausibility	• There has been no reported association between hAdv or adenovirus 41F, or AAV-2 and fulminant hepatitis in healthy children. A recent study examining retrospective liver samples from a cohort of paediatric acute liver failure of unknown cause from 2007-2014 in the US did not find hAdv in any of the 44 liver biopsy samples examined or 19 serum samples examined, showing this has not been historically associated with acute liver failure in children. [85]<br>• There is no evidence for hepatitis post-infection complication of HAdv or AAV-2 thus far in the literature.<br>• Even if it were a post-infection complication, it is unclear why this would happen now, given that adenovirus positivity rates (respiratory and faecal) have been fairly stable between 2017 and 2022.[1]<br>• Some possible explanations include the following:<br>○ Novel mutations in adenovirus may have resulted in a strain that causes hepatitis in immunocompetent children. Thus far, there is no evidence for this. In the 9 suspected cases in Alabama, USA, phylogenetic analysis indicated that three distinct, known viral strains were involved [96]. In the cluster of the nine AAV2 positive cases in Scotland, there were seven distinct but previously identified lineages [68]. Thus, the outbreak is unlikely to be driven by a single, novel strain of adenovirus or AAV2 [97]<br>○ Another explanation proposed is altered immune function, either related to previous COVID-19 infectionf[98] or lack of previous adenovirus exposure because of lockdowns [99], but there is no clear evidence to support either of these. Cases were also seen in regions with lax mitigations or with no lockdowns (like Sweden).	Given positivity for respiratory and faecal adenovirus has not shown an increase over previous years, and the lack of novel strains of adenovirus, and evidence for HAdv and AAV-2 causing these presentations before, it seems more plausible that activation of these is incidental in the immunodeficient state of fulminant hepatitis, than these being causal as of themselves.	We conclude that this criterion is partially met, since recent evidence has found AAV2 RNA in hepatocytes nuclei and/or cytoplasms, supporting a possible hepatitis immunopathological response caused by exposure to AAV2 infection with potential genetic susceptibility. However, it is unclear why this would cause disease now, when this has not caused disease before (except in a context of gene therapy).

	○ The presence of HAdv this cluster may relate to them being persistent viruses that reactivate during severe illness and weakened immunity, or the role of hAdv in being permissive for AAV-2 infection [100 101].<br>○ A bioinformatics meta-analysis found similarities between peptide sequences of the human enzyme AHCY and a viral protein of HAdV-41[94], potentially leading to the production of autoantibodies following HAdV-41 infection. Such autoantibodies may be part of an autoimmune acute hepatitis pathway. However, it is unclear why this would occur now when it has not occurred before, without the virus strain being novel, unless due to unprecedented levels of infection and a rare complication being observed when it was not observed before.		
Coherence	• We were only able to identify a single case report of hAdv causing hepatitis in an immunocompetent child prior to the current cluster. [96 102].<br>• However, there have been multiple reports of adenovirus hepatitis in immunocompromised children. [103-113]	hAdv is not a causative agent for severe hepatitis in healthy children is not coherent with the existing knowledge of either virus	This criterion is only minimally met
Experimental manipulation	• One study found that inoculation with adenovirus in animal models, for example Syrian hamsters, can lead to liver damage and elevated liver enzymes. However this has not been demonstrated on adenovirus type F40 or F41, which have been the predominant strains in the current cluster. [114]		This criterion is only minimally met
Analogy	• While there is little prior evidence of adenovirus hepatitis in immunocompetent children, there are many reports of it in severely immunocompromised children [103-113]. It has been reported in liver transplant recipients, [103 106 111 113] children with acute lymphoblastic leukemia. [104 107 111 112]		This criterion is minimally met for hAdV

Supplementary Table 4: Bradford criteria for AAV-2 as a causal agent for paediatric hepatitis of unknown cause

Criteria	Evidence	Limitations	Inference
Strength	• 26 out of 32 cases of hepatitis were positive for AAV2 with higher estimated copy number relative to all controls (including contemporaneous hospitalized controls).[29]<br>• The median viral load of AAV-2 in five liver biopsies of children affected was significantly higher than in controls (liver biopsies of 19 children under 18 yrs).[29]<br>• Anti-AAV2 IgM was detected in 15 out of 23 (65.2%) samples from cases of hepatitis, but only 1 out of 13 (7.7%) samples from healthy controls and 2 out of 16 (12.5%) samples from the contemporaneous hospitalised controls from Scotland, suggesting recent AAV-2 infection was more likely among cases[29]<br>• In a small study from the Scotland, AAV2 was found in 9/9 (100%) of cases but in none of 13 healthy controls (sampled during an earlier period), or in 12 children with HAdv with normal liver function.[29]<br>• A UK study showed AAV-2 in 5/5 explanted livers among cases and 1/4 among controls (low-level positive).[28]<br>• A study conducted in the UK examined liver biopsy samples from cases and found the presence of AAV2 RNA in the nuclei and/or cytoplasm of ballooned hepatocytes as well as in arterial	• While the AAV-2 association with hepatitis has been identified in small samples, the strength of the association has been consistently strong.<br>• Positivity and median viral load has been higher in cases relative to controls in several studies<br>• Several studies had limitations with respect to controls:<br>Controls were either not contemporaneous, or when contemporaneous, not immunocompromised in several studies, making the groups hard to compare, as AAV-2 can re-activate with immunosuppression, and exposure has varied considerably over time. Only one immunocompetent individual and 17 immunocompromised controls were sampled contemporaneously during the outbreak in one study.[28] However, in this study controls were not age matched to cases, with a median age of 1 compared to 3 in cases. Age has been shown to be strongly associated	Strength criteria for the association between AAV-2 are fully met, as a strong association has been observed with AAV-2 positivity and viral load in studies where this was measured in cases. However, these studies have been limited to the UK and US to date. Furthermore, fulminant hepatitis is a state of immunosuppression, incidental activation cannot be distinguished from causal role for AAV-2, as there are no controls that were immunosuppressed, age-matched and contemporaneous in any study. However, higher IgM levels for AAV-2 in cases compared with controls is more in favour of recent infection than re-activation for AAV-2.

	endothelial cells. This indicates the presence of a replicating virus. AAV2 was not detectable in liver sections from samples of healthy individuals in either the endothelial cells or hepatocytes.[29]<br>• A metagenomics study has found high levels of AAV2 DNA in the liver, blood, plasma, or stool samples from 27 out of 28 cases, with controls having infrequent detection and low levels of AAV2 in their blood or liver samples.[28]<br>• In a US study, AAV-2 was detected in 93% (13 of 14) of cases of acute severe hepatitis of unknown aetiology, compared to 3.5% (4 of 113) of controls.[89]	with AAV-2 seroprevalence, with seroprevalence being lowest in under 1 year olds[115] (as 12 out of 17 controls were), which could potentially lead to a false positive association here.	
Consistency	• <u>See strength of association for studies</u><br>• Studies examining the association between AAV-2 and hepatitis of unknown cause in children have consistently shown strong associations, although sample sizes have been small, studies are limited to primarily the UK and US, and controls in no study so far have not been simultaneously matched for immunosuppression, age, and period of sampling.	There appears to be consistency in the association between AAV-2 and cases of hepatitis in studies to date. However, our assessment is limited given the limited number of studies that have examined this, and lack of appropriate controls.	This criterion is partially met for the association between AAV-2 and hepatitis
Specificity	• Between 20-40% of cases also tested positive for known latent viruses like EBV, HHV-6 and HHV-7,[1] likely to indicate reactivation<br>• Of five explanted livers from the UK, metagenomics identified high levels of AAV-2, and low levels of HAdv, and HHV-6B[1 28] (all persistent	Multiple viruses were recovered both in liver tissue and other samples among cases, and controls, including HHV-6, HAdV and AAV-2. This suggests the possibility that at least some of these may represent activation of latent	AAV2 was not specific to cases as hAdv, EBV, HHV-6 were all identified to be enriched in some case series. However, it seems more specific to cases as it is present at higher levels, and in a

	viruses) in all livers, indicating possible re-activation of virus due to severe fulminant hepatitis, or AAV2 as a causal agent alongside a permissive virus.<br>• AAV-2 PCR was positive in 9/9 formalin-fixed paraffin-embedded (FFPE) liver samples, HHV-6B PCR in 6/9 samples and HAdV in 4/9 samples.[28]<br>27 of 28 cases tested were AAV2 PCR positive (high levels), 23 of 31 were HAdV positive and 16 of 23 were HHV-6B positive (low levels). [28] AAV-2 was also detected in younger immunosuppressed controls but at lower frequency (11/32) as was HHV-6B (5/15).<br>• HHV-6 was also overrepresented in cases in a small study[93] which may point to re-activation of persistent viruses in the relative immuondeficient state of severe fulminant hepatitis, or presence of a virus permissive to AAV2 replication.[29]<br>• Co-infections by Epstein–Barr virus, human herpesvirus 6 and/or enterovirus A71 were also detected in 12 (85.7%) of 14 cases, with higher herpesvirus detection in cases versus controls ($P < 0.001$). The association of HAdv was not tested as cases were selected based on HAdv positivity<br>• While HAdv41F has been the dominant subtype of HAdv identified, other subtypes have also been identified in the UK, and in other countries.<br>• Cases with hepatitis of unknown aetiology contained AAV2 RNA in nuclei and/or cytoplasm of ballooned hepatocytes as well as in arterial	viruses in a critically ill host. AAV-2 was most frequent in samples and enriched in cases compared to controls, where examined. However, studies that examined this were limited, and sample sizes were small. AAV-2 was not specific to cases, and was present in immunosuppressed controls, but at significantly lower frequency and viral load.[28]	higher frequency of cases. The presence of other viruses may represent the need for permissive viruses for AAV-2 replication. However when comparisons are made with immunosuppressed controls (not age matched), AAV-2 was still present in a third of controls. Given the lack of appropriate controls in almost all studies, with controls being younger in the only study of immunosuppressed controls, and therefore less likely to have been exposed to AAV-2, these findings are difficult to interpret. AAV-2 is expected to activate and replicate in immunosuppressed states such as fulminant hepatitis, which requires adequate control groups that are not only immunosuppressed, but of similar age, as AAV-2 exposure varies markedly with age in children. Thus, the criterion for specificity is only partially met for AAV-2.

	endothelial cells. The levels of AAV2-positive cells ranged from 1.2% to 4.7% in these biopsies.[29]		
Temporality	• A study in Ireland showed an increase in wastewater detection of AAV-2 and hAdV-F concurrent with the period of detection of cases of hepatitis of unknown cause[86]<br>• Given most cases were tested at the point of admission for hepatitis, a positive test for HAdv or AAV-2 can be considered as evidence of being infected at the time of presentation.[96]<br>• It is important to delineate acute infection from re-activation of virus and the temporal association of these with hepatitis. As fulminant hepatitis in itself is considered a state of immunosuppression, and most cases were in this state at the point of testing, it is difficult to determine whether HAdv and/or AAV-2 infection/activation preceded hepatitis, or followed it.<br>• A case-control study in Scotland found that 100% of nine suspected cases were positive for AAV2, which first requires infection with a 'helper' virus such as adenovirus. Many of these cases had a history of gastrointestinal illness several weeks before the onset of hepatitis symptoms. This suggests that hepatitis may have been preceded by adenovirus infection or reactivation[68].<br>• The 12 transplanted cases reported gastrointestinal symptoms (nausea, vomiting and	The presence of AAV2 on admission may be indicative of a recent adenovirus infection or reactivation which may have preceded hepatitis, or followed it due to fulminant hepatitis being an immunosuppressed state. However, studies from the UK and Scotland have found higher median AAV-2 viral loads in cases compared to contemporaneous controls, and higher proportion of IgM positivity, which is likely to be compatible with recent infection[29]	We consider this criterion partially met for AAV-2

	diarrhoea) preceding transplant by a median of 20 days (range 8–42 days)[28]<br>• IgM positivity to AAV-2 among cases was higher than contemporaneous controls, suggesting recent infection was more likely in cases		
Biological gradient	• A metagenomic study has shown that 27 of 28 of cases, 96% were positive for AAV2 compared to 6 of 17 (35% controls), and cases had significantly lower PCR Ct levels, suggesting higher viral loads.[28]<br>• The median viral load of AAV-2 in five liver biopsies of children affected was significantly higher than in controls (liver biopsies of 19 children under 18 yrs).[29]	One study showed that immunocompromised controls had higher AAV-2 levels than healthy controls (indicating activation), but lower than in cases.[28] This provides some evidence of a biological gradient. However, due to potentially inadequate matching for age in immunosuppressed controls these findings must be interpreted with caution, and could represent viral reactivation.	This criterion is partially met for AAV-2.
Plausibility	There has been no reported association between AAV-2 and fulminant hepatitis in healthy children.<br>• There is no evidence for hepatitis post-infection complication of AAV-2 thus far in the literature.<br>• Even if it were a post-infection complication, it is unclear why this would happen now.<br>Some possible explanations include the following:<br>○ Novel mutations in AAV-2 may have resulted in a strain that causes hepatitis in immunocompetent children. In the cluster of the nine AAV2 positive cases in Scotland, there were seven distinct but previously	Given AAV-2 has never been known to cause this presentations before, it seems more plausible that activation of this is incidental in the immunodeficient state of fulminant hepatitis, or perhaps related to re-activation due to prior COVID-19 infection in hosts with particular genetic predispositions.	We conclude that this criterion is partially met, since recent evidence has found AAV2 RNA in hepatocytes nuclei and/or cytoplasms, supporting a possible hepatitis immunopathological response caused by exposure to AAV2 infection with potential genetic susceptibility. However, it is unclear why this would cause disease now, when this has not caused disease before (except in a context of gene therapy).

	identified lineages [68]. Thus, the outbreak is unlikely to be driven by a single, novel strain of adenovirus or AAV2 [97]<br>○ Another explanation proposed is altered immune function, either related to previous COVID-19 infection or lack of previous adenovirus exposure because of lockdowns [99], but there is no clear evidence to support either of these possibilities. Regions such as Sweden where lockdowns were not in place have also seen hepatitis cases. COVID-19 has been shown to re-activate latent viruses, which may provide an explanation.<br>○ The presence of AAV2 in this cluster may relate to them being persistent viruses that reactivate during severe illness and weakened immunity [100 101].<br>○ In a study conducted in the UK, liver biopsy tissue samples from cases exhibited viral AAV2 RNA within the nucleus and cytoplasm of ballooned hepatocytes, as well as a dense infiltration of $CD4^+$ and $CD8^+$ T cells in the liver displaying an activated phenotype. The findings suggest the likelihood of a immunopathological response caused by exposure to AAV2 infection.[29]<br>○ Multiple factors increasing susceptibility to AAV-2 including genetic factors. The MHC class II HLA-DRB1*04:01 allele was enriched		

	in affected children, indicating a potential association.[29]<br>○ A bioinformatics meta-analysis found similarities between peptide sequences of the human enzyme AHCY and a viral protein of HAdV-41[94], potentially leading to the production of autoantibodies following HAdV-41 infection. Such autoantibodies may be part of an autoimmune acute hepatitis pathway<br>○ Hepatitis following AAV gene therapy has been well described[116-118] with deaths occurring, however rarely.		
Coherence	• AAV2 is not known to cause disease in humans, except rarely in the context of AAV gene therapy. [119]	AAV2 as causative agents for severe hepatitis in healthy children is not coherent with the existing knowledge of either virus	This criterion is only minimally met
Experimental manipulation	• Hepatitis following AAV gene therapy has been well described[116-118] with deaths occurring, however rarely.		This criterion is minimally met
Analogy	• Hepatitis following AAV gene therapy has been described[116-118] with deaths occurring, however rarely.		This criterion is minimally met<br>AAV-2

Supplementary Table 5: Estimated AIC and mean squared errors of predictive models

DLNM/DLM model	Exposure-outcome	Lag-outcome	AIC-covid	MSE	AIC-Adv-resp	MSE	AIC-Adv-faecal	MSE
Model 1	linear	unconstrained	169.5	42.2	205.0	104.6	309.0	87.1
Model 2	Polynomial degree=2	unconstrained	176.4	35.7	192.4	50.2	315.3	74.8
Model 3	linear	Polynomial degree=2	194.4	117.6	202.6	208.4	315.9	108.7
Model 4	linear	Polynomial degree=3	184.3	90.8	204.6	158.2	310.06	100.7
Model 5	linear	Polynomial degree=4	181.8	82.5	212.3	157.8	311.3	100.1
Model 6	Polynomial degree=2	Polynomial degree=4	187.3	74.9	203.1	104.8	306.7	86.9
Model 7	Polynomial degree=3	Polynomial degree=4	187.2	60.4	182.3	54.5	300.3	74.0
Model 8	Polynomial degree=4	Polynomial degree=4	178.2	40.2	186.3	47.9	304.7	70.2
Model 9	linear	Natural cubic spline df=4	190.7	104.0	210.3	158.0	309.9	100.6
Model 10	Polynomial degree=2	Natural cubic spline df=4	191.3	88.9	200.0	106.6	311.2	94.2
Model 11	Polynomial degree=3	Natural cubic spline df=4	187.2	60.5	187.2	68.6	316.2	91.6
Model 12	Polynomial degree=4	Natural cubic spline df=4	178.1	40.3	185.1	55.4	318.3	86.4
Model 13	Natural cubic spline df=4	Natural cubic spline df=4	192.8	65.2	185.0	55.2	316.1	84.6

AIC: Akaike Information criterion; MSE: Mean squared error

Supplementary Figure 1: Adenovirus respiratory sample positivity and positive respiratory and faecal specimens in young children

a)

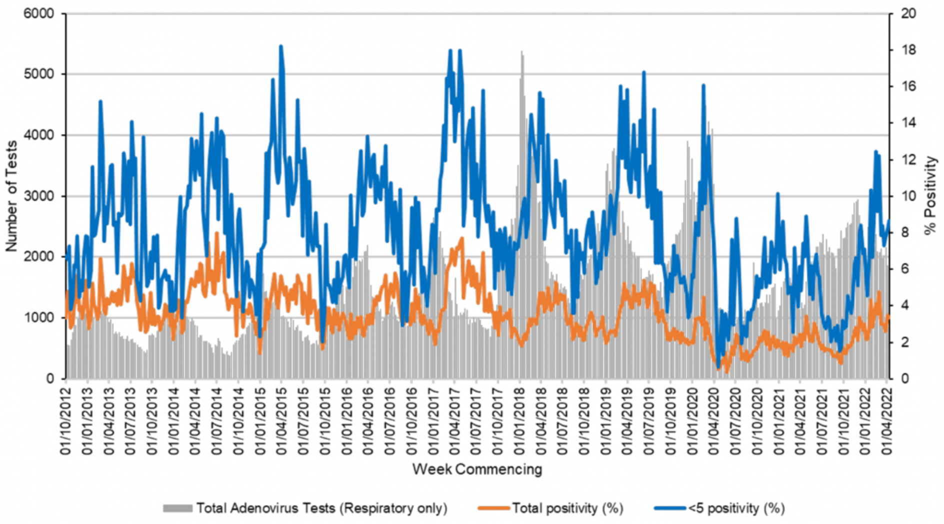

b)

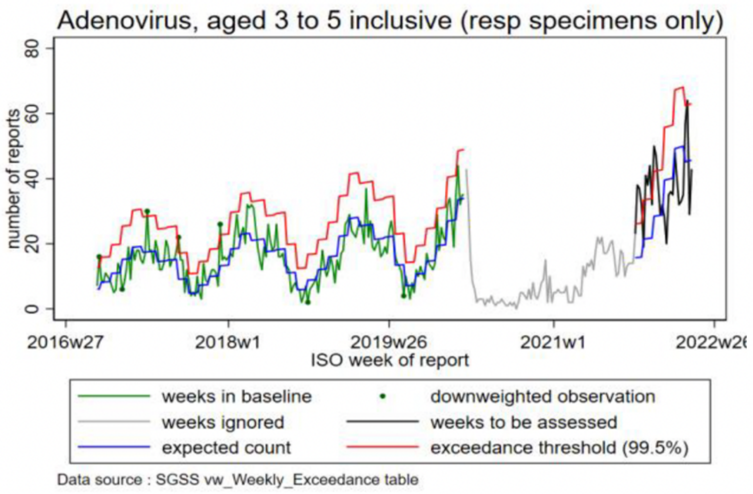

c)

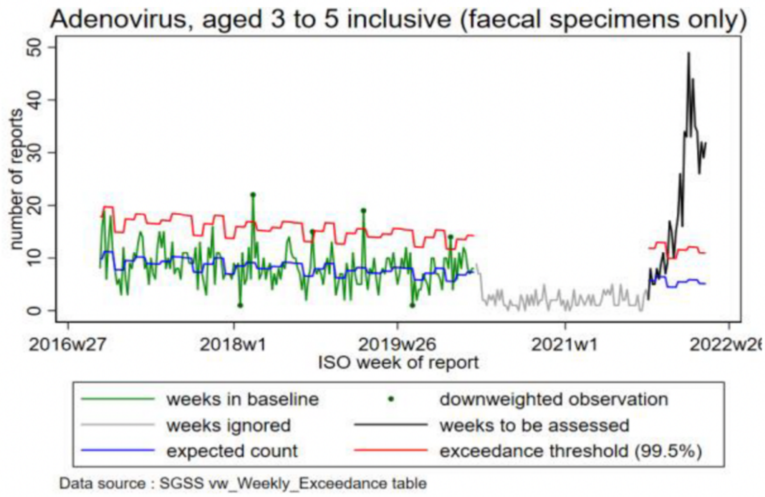

Supplementary Figure 1, reproduced from UKHSA technical report 4 on hepatitis[1] shows total adenovirus test positivity (1a) (respiratory samples only) over time (orange line), positivity for children under 5 years (blue line) and number of tests conducted (in grey). Positivity has not shown increase over previous years, but tests carried out in summer ’21 appear higher than previous years, suggesting better ascertainment. Consistently, the number of specimens positive for adenovirus shows an increase for respiratory (1b) and faecal (1c) specimens compared to previous years when not adjusted for the number of tests.

Supplementary Figure 2: Excess attendances in the age group 1-4 years with a liver condition from January 2020 to end of June 2022

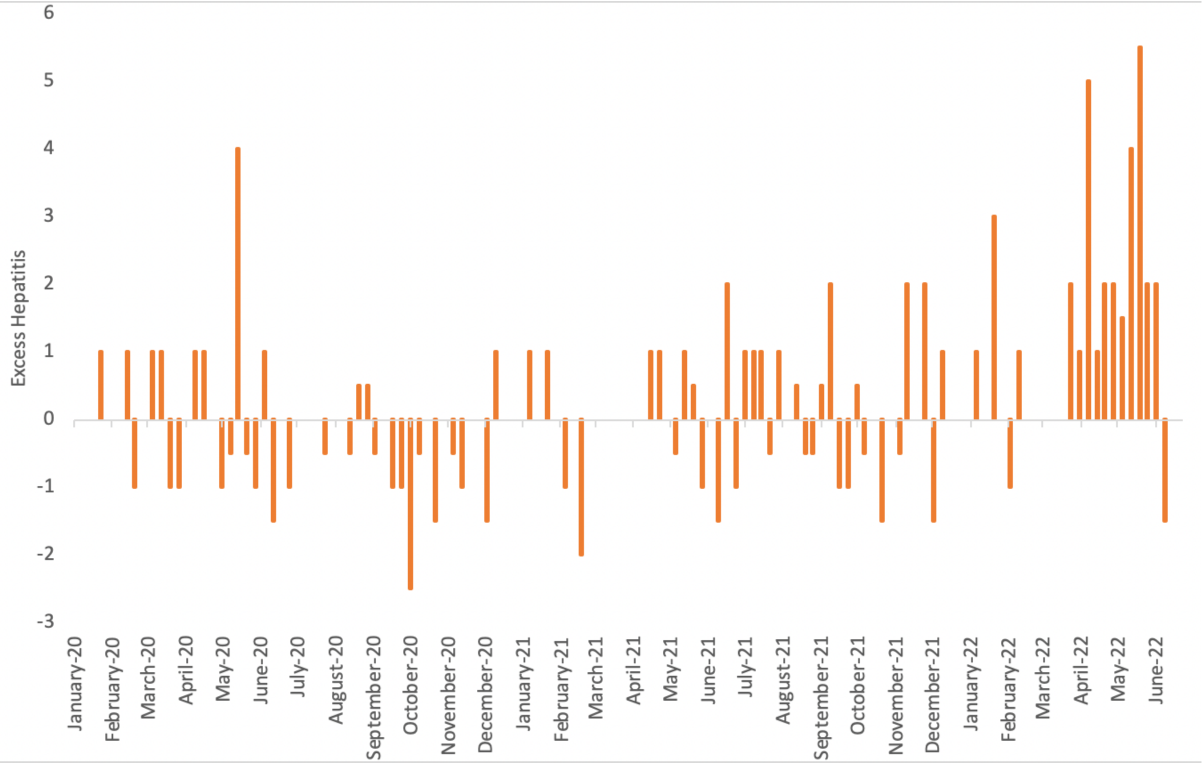

Supplementary Figure 2 shows excess attendances for liver conditions in the age group 1-4 years between 2020-June 2022 calculated against a baseline from 2018-2019.

Supplementary Figure 3: Adenovirus respiratory sample positivity superimposed with different lags to excess hepatitis in 1-4 year olds

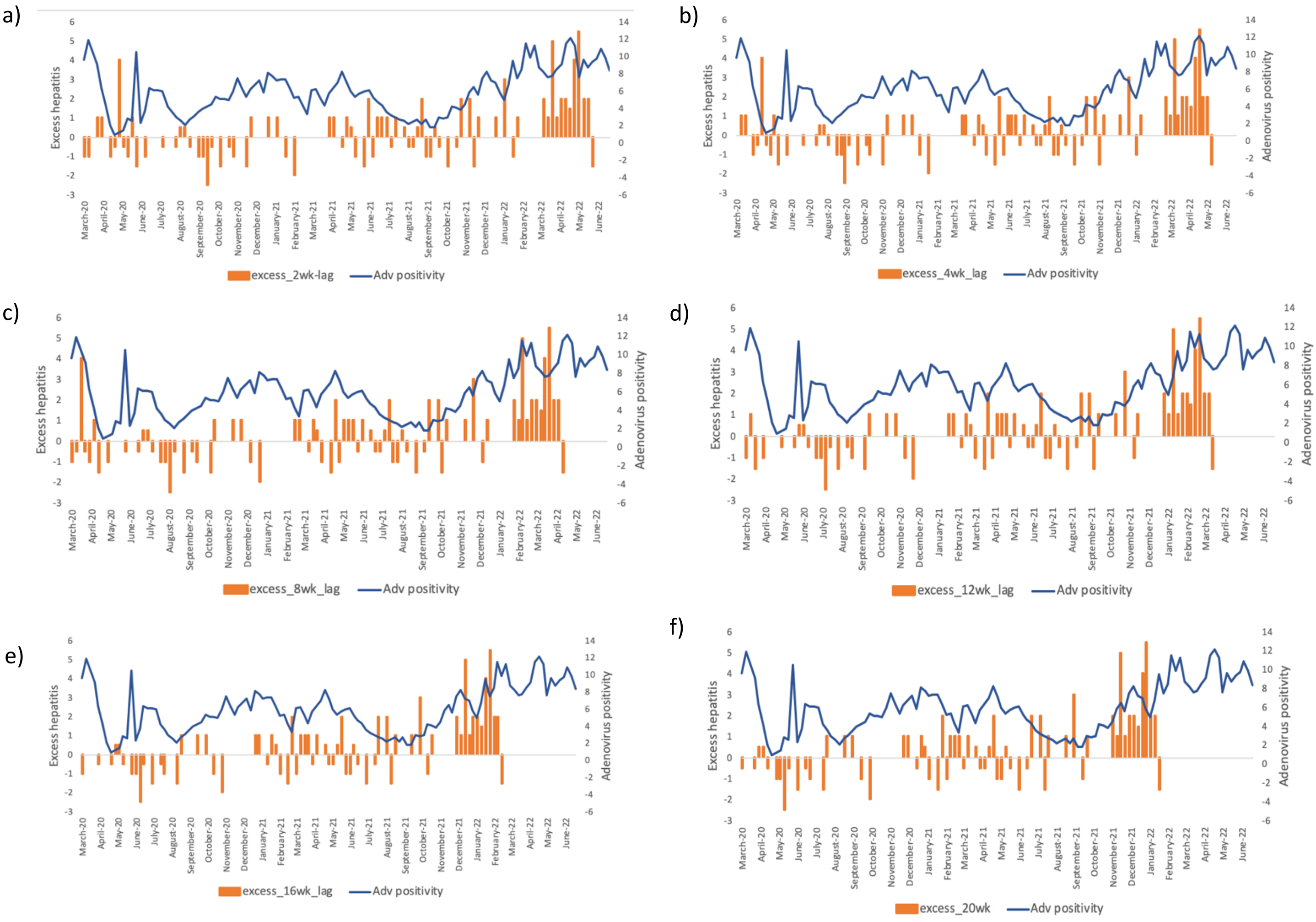

Supplementary Figure 3 shows superimposed plots of excess attendances to A&E for liver conditions among 1-4 year olds (orange bars) lagged by 2 wks, 4wks, 8wks, 12wks, 16 wks and 20wks (Figures 4a, 4b, 4c, 4d, 4e and 4f, respectively) onto Adv respiratory sample positivity (blue line).

Supplementary Figure 4: Association of excess hepatitis with positive faecal specimens at different time lags

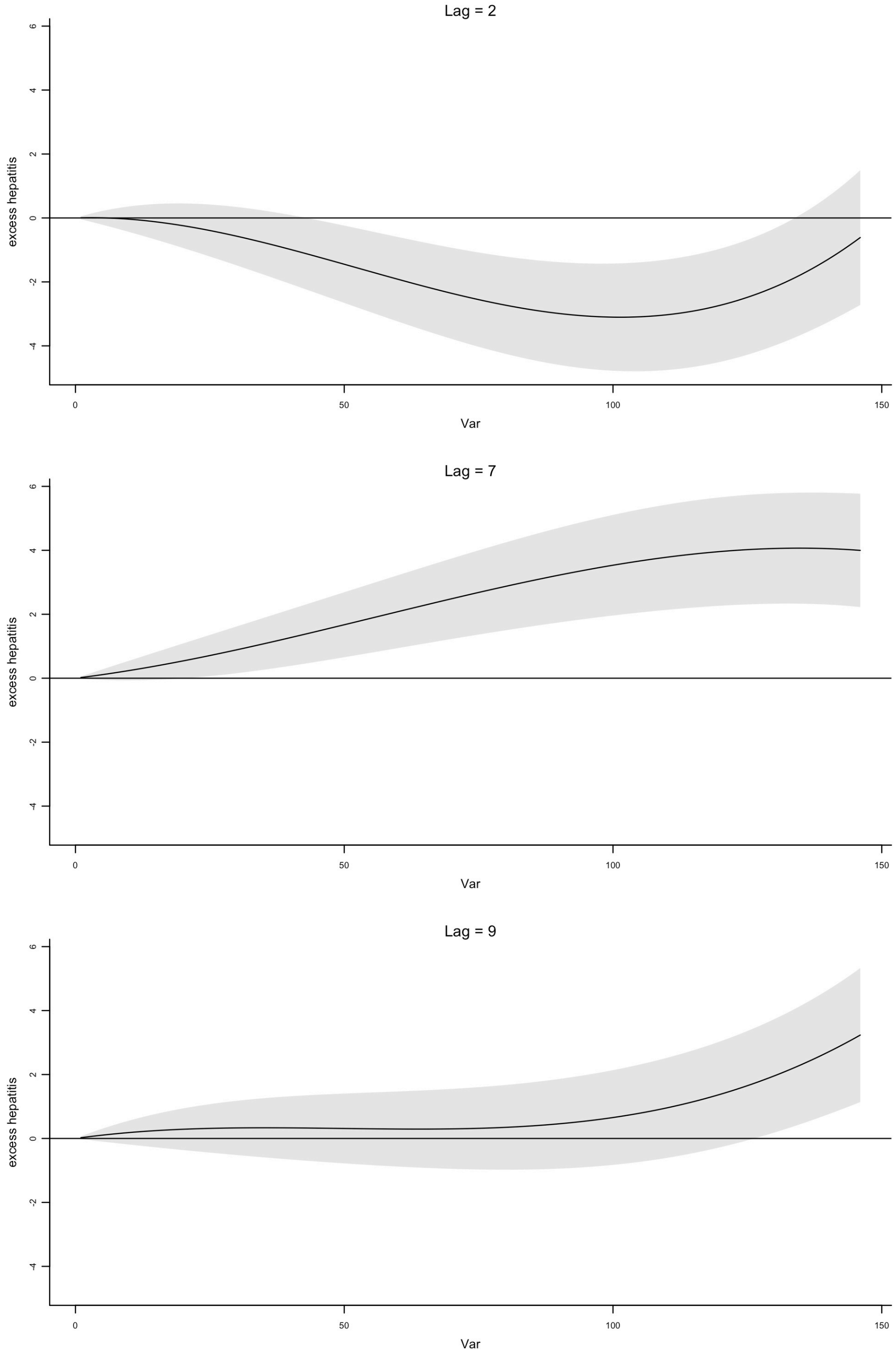

Supplementary Figure 4 show the varying relationship between exposure (number of faecal specimens positive for Adv), and excess A&E attendances for liver conditions in 1-4 year olds at different time lags. A non-linear positive association between number of positive faecal specimens positive for Adv (x axis) and excess hepatitis is seen at a lag of 7 fortnights, while the association at a lag of 2 fortnights is negative.

References

1. UKHSA. Investigation into acute hepatitis of unknown aetiology in children in England: Technical briefing 4, 2022.
2. Cates J, Lucero-Obusan C, Dahl RM, et al. Risk for In-Hospital Complications Associated with COVID-19 and Influenza - Veterans Health Administration, United States, October 1, 2018-May 31, 2020. *MMWR Morb Mortal Wkly Rep* 2020;69(42):1528-34. doi: 10.15585/mmwr.mm6942e3 [published Online First: 2020/10/23]
3. Zhang Q, Li J, Zhang Y, et al. Differences in clinical characteristics and liver injury between suspected and confirmed COVID-19 patients in Jingzhou, Hubei Province of China. *Medicine (Baltimore)* 2021;100(19):e25913. doi: 10.1097/md.0000000000025913 [published Online First: 2021/06/10]
4. Osman M, Klopfenstein T, Belfeki N, et al. A Comparative Systematic Review of COVID-19 and Influenza. 2021;13(3):452.
5. Pirisi M, Rigamonti C, D'Alfonso S, et al. Liver infection and COVID-19: the electron microscopy proof and revision of the literature. *Eur Rev Med Pharmacol Sci* 2021;25(4):2146-51. doi: 10.26355/eurrev_202102_25120 [published Online First: 2021/03/05]
6. Wang Y, Liu S, Liu H, et al. SARS-CoV-2 infection of the liver directly contributes to hepatic impairment in patients with COVID-19. *J Hepatol* 2020;73(4):807-16. doi: 10.1016/j.jhep.2020.05.002 [published Online First: 2020/05/22]
7. Wanner N, Andrieux G, Badia-i-Mompel P, et al. Molecular consequences of SARS-CoV-2 liver tropism. *Nature Metabolism* 2022;4(3):310-19. doi: 10.1038/s42255-022-00552-6
8. Antala S, Diamond T, Kociolek LK, et al. Severe Hepatitis in Pediatric Coronavirus Disease 2019. *J Pediatr Gastroenterol Nutr* 2022;74(5):631-35. doi: 10.1097/mpg.0000000000003404 [published Online First: 2022/02/13]
9. Brisca G, Mallamaci M, Tardini G, et al. SARS-CoV-2 Infection May Present as Acute Hepatitis in Children. *The Pediatric Infectious Disease Journal* 2021;40(5)
10. Zhou YH, Zheng KI, Targher G, et al. Abnormal liver enzymes in children and infants with COVID-19: A narrative review of case-series studies. *Pediatr Obes* 2020;15(12):e12723. doi: 10.1111/ijpo.12723 [published Online First: 2020/09/03]
11. Wang J, Hu W, Wang K, et al. Case report: Acute hepatitis in neonates with COVID-19 during the Omicron SARS-CoV-2 variant wave: a report of four cases. *Front Pediatr* 2023;11:1179402. doi: 10.3389/fped.2023.1179402 [published Online First: 2023/05/22]
12. Greenberg GM, Salman SS. COVID-19 Presenting With Acute Anicteric Hepatitis in Pediatric Patient: A Case Report. *JPGN Rep* 2022;3(3):e236. doi: 10.1097/PG9.0000000000000236 [published Online First: 2023/05/12]
13. Chen X, Hong J, Li Y, et al. Case report: Severe acute hepatitis in a 22-month-old Chinese boy with Omicron sub-variant BA.2.38. *Front Public Health* 2022;10:1012638. doi: 10.3389/fpubh.2022.1012638 [published Online First: 2022/12/13]
14. Osborn J, Szabo S, Peters AL. Pediatric Acute Liver Failure Due to Type 2 Autoimmune Hepatitis Associated With SARS-CoV-2 Infection: A Case Report. *JPGN Rep* 2022;3(2):e204. doi: 10.1097/PG9.0000000000000204 [published Online First: 2022/05/05]

15. Perez A, Cantor A, Rudolph B, et al. Liver involvement in children with SARS-COV-2 infection: Two distinct clinical phenotypes caused by the same virus. *Liver Int* 2021;41(9):2068-75. doi: 10.1111/liv.14887 [published Online First: 2021/04/08]
16. Cooper S, Tobar A, Konen O, et al. Long COVID-19 Liver Manifestation in Children. *Journal of Pediatric Gastroenterology and Nutrition* 2022
17. Kendall EK OV, Kaelber DC, Xu R, Davis PB. Elevated liver enzymes and bilirubin following SARS-CoV-2 infection in children under 10. *MedRxiv* 2022
18. Lexmond WS, de Meijer VE, Scheenstra R, et al. Indeterminate pediatric acute liver failure: Clinical characteristics of a temporal cluster of five children in the Netherlands in the spring of 2022. *United European Gastroenterol J* 2022;10(8):795-804. doi: 10.1002/ueg2.12269 [published Online First: 2022/07/01]
19. Ratho R.K. AAA, Mishra N., Jain A., Rawat S.K. COVID-19 Associated Hepatitis in Children (CAH-C) during the second wave of SARS-CoV-2 infections in Central India: is it a complication or transient phenomenon. *MedRxiv* 2021
20. ONS. Coronavirus (COVID-19) Infection Survey, antibody data, UK: 1 June 2022, 2022.
21. Kleinhenz J, Wagner E, Afzal SY, et al. Unique hepatic manifestations of COVID-19-induced immune dysregulation in children. *Clin Case Rep* 2022;10(11):e6510. doi: 10.1002/ccr3.6510 [published Online First: 2022/11/24]
22. Romani Vidal A, Vaughan A, Innocenti F, et al. Hepatitis of unknown aetiology in children - epidemiological overview of cases reported in Europe, 1 January to 16 June 2022. *Euro Surveill* 2022;27(31) doi: 10.2807/1560-7917.ES.2022.27.31.2200483 [published Online First: 2022/08/06]
23. The UK Health Security Agency. Investigation into acute hepatitis of unknown aetiology in children in England: Technical briefing 4 2022 [Available from: https://assets.publishing.service.gov.uk/government/uploads/system/uploads/attachment_data/file/1094573/acute-hepatitis-technical-briefing-4.pdf.
24. European Centre for Disease Prevention and Control/WHO Regional Office for Europe. Hepatitis of Unknown Aetiology in Children, Joint Epidemiological overview, 30 June World Health Organization2022 [Available from: https://cdn.ecdc.europa.eu/novhep-surveillance/.
25. Health Protection Surveillance Center (HPSC) of Ireland. Severe acute hepatitis cases of unknown aetiology in Ireland 2022 [Available from: https://www.hpsc.ie/a-z/hepatitis/acutehepatitisofunknownaetiology/dataandreports/20220728_Epi%20summary%20of%20cases%20hepatitis%20unknown%20aetiology.pdf.
26. Weisberg SP, Connors TJ, Zhu Y, et al. Distinct antibody responses to SARS-CoV-2 in children and adults across the COVID-19 clinical spectrum. *Nature Immunology* 2021;22(1):25-31. doi: 10.1038/s41590-020-00826-9
27. Ladhani SN, Ireland G, Baawuah F, et al. SARS-CoV-2 infection, antibody positivity and seroconversion rates in staff and students following full reopening of secondary schools in England: A prospective cohort study, September-December 2020. *EClinicalMedicine* 2021;37:100948. doi: 10.1016/j.eclinm.2021.100948 [published Online First: 2021/08/14]
28. Morfopoulou S, Buddle S, Torres Montaguth OE, et al. Genomic investigations of unexplained acute hepatitis in children. *Nature* 2023;617(7961):564-73. doi: 10.1038/s41586-023-06003-w [published Online First: 2023/03/31]

29. Ho A, Orton R, Tayler R, et al. Adeno-associated virus 2 infection in children with non-A-E hepatitis. *Nature* 2023;617(7961):555-63. doi: 10.1038/s41586-023-05948-2 [published Online First: 2023/03/31]
30. I E. Israel Examining 12 Cases of Kids' Hepatitis After WHO Warning. *Haaretz, Israel News* 2022.
31. United States Centers for Disease Control and Prevention. Technical Report: Acute Hepatitis of Unknown Cause: U.S. Department of Health & Human Services; 2022 [Available from: https://www.cdc.gov/ncird/investigation/hepatitis-unknown-cause/technical-report.html.
32. Romaní Vidal A, Vaughan A, Innocenti F, et al. Hepatitis of unknown aetiology in children – epidemiological overview of cases reported in Europe, 1 January to 16 June 2022. 2022;27(31):2200483. doi: doi:https://doi.org/10.2807/1560-7917.ES.2022.27.31.2200483
33. World Health Organization. Severe acute hepatitis of unknown aetiology in children - Multi-country: WHO; 2022 [Available from: https://www.who.int/emergencies/disease-outbreak-news/item/2022-DON400.
34. Elbeltagi R, Al-Beltagi M, Saeed NK, et al. May 2022 acute hepatitis outbreak, is there a role for COVID-19 and other viruses? *World J Hepatol* 2023;15(3):364-76. doi: 10.4254/wjh.v15.i3.364 [published Online First: 2023/04/11]
35. ECDC W. Joint ECDC-WHO Regional Office for Europe Hepatitis of Unknown Origin in Children Surveillance Bulletin, 2022.
36. Eales O, de Oliveira Martins L, Page AJ, et al. Dynamics of competing SARS-CoV-2 variants during the Omicron epidemic in England. *Nat Commun* 2022;13(1):4375. doi: 10.1038/s41467-022-32096-4 [published Online First: 2022/07/29]
37. ONS. Coronavirus (COVID-19) latest insights: Antibodies, 2022.
38. Feldstein LR, Rose EB, Horwitz SM, et al. Multisystem Inflammatory Syndrome in U.S. Children and Adolescents. *N Engl J Med* 2020;383(4):334-46. doi: 10.1056/NEJMoa2021680 [published Online First: 2020/07/01]
39. Gov.uk. Coronavirus (COVID-19) in the UK, 2022.
40. Fan H, Cai J, Tian A, et al. Comparison of Liver Biomarkers in 288 COVID-19 Patients: A Mono-Centric Study in the Early Phase of Pandemic. *Frontiers in Medicine* 2020;7 (no pagination)
41. Higuera-de la Tijera F, Servin-Caamano A, Reyes-Herrera D, et al. Impact of liver enzymes on SARS-CoV-2 infection and the severity of clinical course of COVID-19. *Liver Research* 2021;5(1):21-27.
42. Hundt MA, Deng Y, Ciarleglio MM, et al. Abnormal Liver Tests in COVID-19: A Retrospective Observational Cohort Study of 1,827 Patients in a Major U.S. Hospital Network. *Hepatology* 2020;72(4):1169-76. doi: 10.1002/hep.31487 [published Online First: 2020/07/30]
43. Kariyawasam JC, Jayarajah U, Abeysuriya V, et al. Involvement of the Liver in COVID-19: A Systematic Review. *Am J Trop Med Hyg* 2022;106(4):1026-41. doi: 10.4269/ajtmh.21-1240 [published Online First: 2022/02/25]
44. Leo M, Galante A, Pagnamenta A, et al. Hepatocellular liver injury in hospitalized patients affected by COVID-19: Presence of different risk factors at different time points. *Dig Liver Dis* 2022;54(5):565-71. doi: 10.1016/j.dld.2021.12.014 [published Online First: 2022/01/31]

45. Abdelrahman MM, Abdel-Baset AA, Younis MA, et al. Liver function test abnormalities in COVID-19 patients and factors affecting them - a retrospective study. *Clinical and Experimental Hepatology* 2021;7(3):297-304.
46. Qu J, Zhu HH, Huang XJ, et al. Abnormal indexes of liver and kidney injury markers predict severity in covid-19 patients. *Infection and Drug Resistance* 2021;14:3029-40.
47. Zhao W, Zhang X, Zhu F, et al. Dynamic Changes of Liver Function Indexes in Patients with Different Clinical Types of COVID-19. *International Journal of General Medicine* 2022;15:877-84.
48. Shafran N, Issachar A, Shochat T, et al. Abnormal liver tests in patients with SARS-CoV-2 or influenza - prognostic similarities and temporal disparities. *JHEP Reports* 2021;3(3) (no pagination)
49. Ryan KA, Bewley KR, Fotheringham SA, et al. Dose-dependent response to infection with SARS-CoV-2 in the ferret model and evidence of protective immunity. *Nat Commun* 2021;12(1):81. doi: 10.1038/s41467-020-20439-y [published Online First: 2021/01/06]
50. Chu H, Bai T, Chen L, et al. Multicenter Analysis of Liver Injury Patterns and Mortality in COVID-19. *Frontiers in Medicine* 2020;7 (no pagination)
51. Da BL, Kushner T, El Halabi M, et al. Liver Injury in Patients Hospitalized with Coronavirus Disease 2019 Correlates with Hyperinflammatory Response and Elevated Interleukin-6. *Hepatology Communications* 2021;5(2):177-88.
52. Jiang S, Wang R, Li L, et al. Liver Injury in Critically Ill and Non-critically Ill COVID-19 Patients: A Multicenter, Retrospective, Observational Study. *Frontiers in Medicine* 2020;7 (no pagination)
53. Mishra K, Naffouj S, Gorgis S, et al. Liver Injury as a Surrogate for Inflammation and Predictor of Outcomes in COVID-19. *Hepatology Communications* 2021;5(1):24-32.
54. Sadeghi A, Eslami P, Moghadam AD, et al. Risk factors related to liver injury in non-Intensive Care Unit admitted patients infected with COVID-19: A retrospective study of 102 patients. *Caspian Journal of Internal Medicine* 2020;11:S520-S26.
55. Voiosu A, Roman A, Pop R, et al. Characteristics and outcomes of patients with COVID-19 and liver injury: a retrospective analysis and a multicenter experience. *Rom J Intern Med* 2022;60(1):49-55. doi: 10.2478/rjim-2021-0027 [published Online First: 2021/07/13]
56. Huang H, Li H, Chen S, et al. Prevalence and Characteristics of Hypoxic Hepatitis in COVID-19 Patients in the Intensive Care Unit: A First Retrospective Study. *Frontiers in Medicine* 2020;7 (no pagination)
57. Kolesova O, Vanaga I, Laivacuma S, et al. Intriguing findings of liver fibrosis following COVID-19. *BMC Gastroenterol* 2021;21(1):370. doi: 10.1186/s12876-021-01939-7 [published Online First: 2021/10/13]
58. Brisca G, Mallamaci M, Tardini G, et al. SARS-CoV-2 Infection May Present as Acute Hepatitis in Children. *Pediatr Infect Dis J* 2021;40(5):e214-e15. doi: 10.1097/inf.0000000000003098 [published Online First: 2021/02/17]
59. Cui Y, Tian M, Huang D, et al. A 55-Day-Old Female Infant Infected With 2019 Novel Coronavirus Disease: Presenting With Pneumonia, Liver Injury, and Heart Damage. *J Infect Dis* 2020;221(11):1775-81. doi: 10.1093/infdis/jiaa113 [published Online First: 2020/03/18]

60. Shaiba LA, Hadid A, Altirkawi KA, et al. Case Report: Neonatal Multi-System Inflammatory Syndrome Associated With SARS-CoV-2 Exposure in Two Cases From Saudi Arabia. *Frontiers in Pediatrics* 2021;9 (no pagination)
61. Cantor A, Miller J, Zachariah P, et al. Acute Hepatitis Is a Prominent Presentation of the Multisystem Inflammatory Syndrome in Children: A Single-Center Report. *Hepatology* 2020;72(5):1522-27. doi: https://doi.org/10.1002/hep.31526
62. Sgouropoulou V, Vargiami E, Kyriazi M, et al. Transient Severe Liver Injury: A Unique Presentation of COVID-19 Disease in a Pediatric Patient. *Pediatr Infect Dis J* 2021;40(5):e204-e05. doi: 10.1097/inf.0000000000003104 [published Online First: 2021/02/17]
63. Li MY, Li L, Zhang Y, et al. Expression of the SARS-CoV-2 cell receptor gene ACE2 in a wide variety of human tissues. *Infect Dis Poverty* 2020;9(1):45. doi: 10.1186/s40249-020-00662-x [published Online First: 2020/04/30]
64. Zou H, Lu J, Liu J, et al. Characteristics of pediatric multi-system inflammatory syndrome (PMIS) associated with COVID-19: a meta-analysis and insights into pathogenesis. *International Journal of Infectious Diseases* 2021;102:319-26. doi: https://doi.org/10.1016/j.ijid.2020.11.145
65. Elilarasi S, Poovazhagi V, Kumaravel G, et al. Pediatric Inflammatory Multisystem Syndrome Temporally Associated with SARS-CoV-2. *Indian Journal of Pediatrics* 2021 doi: 10.1007/s12098-021-03954-8
66. Giannattasio A, Maglione M, D'Anna C, et al. Liver and Pancreatic Involvement in Children with Multisystem Inflammatory Syndrome Related to SARS-CoV-2: A Monocentric Study. *Children* 2022;9(4):575.
67. Lazova S, Alexandrova T, Gorelyova-Stefanova N, et al. Liver Involvement in Children with COVID-19 and Multisystem Inflammatory Syndrome: A Single-Center Bulgarian Observational Study. *Microorganisms* 2021;9(9) doi: 10.3390/microorganisms9091958
68. Ho A, Orton R, Tayler R, et al. Adeno-associated virus 2 infection in children with non-A-E hepatitis. 2022:2022.07.19.22277425. doi: 10.1101/2022.07.19.22277425 %J medRxiv
69. De Silvestri A, Capittini C, Poddighe D, et al. HLA-DRB1 alleles and juvenile idiopathic arthritis: Diagnostic clues emerging from a meta-analysis. *Autoimmunity reviews* 2017;16(12):1230-36. doi: 10.1016/j.autrev.2017.10.007 [published Online First: 2017/10/19]
70. Langton DJ, Bourke SC, Lie BA, et al. The influence of HLA genotype on the severity of COVID-19 infection. 2021;98(1):14-22. doi: https://doi.org/10.1111/tan.14284
71. Langton DJ, Bourke SC, Lie BA, et al. The influence of HLA genotype on the severity of COVID-19 infection. *HLA* 2021;98(1):14-22. doi: 10.1111/tan.14284 [published Online First: 2021/04/26]
72. Ebrahimi S, Ghasemi-Basir HR, Majzoobi MM, et al. HLA-DRB1*04 may predict the severity of disease in a group of Iranian COVID-19 patients. *Human immunology* 2021;82(10):719-25. doi: 10.1016/j.humimm.2021.07.004 [published Online First: 2021/07/24]
73. Augusto DG, Murdolo LD, Chatzileontiadou DSM, et al. A common allele of HLA is associated with asymptomatic SARS-CoV-2 infection. *Nature* 2023;620(7972):128-36. doi: 10.1038/s41586-023-06331-x [published Online First: 2023/07/20]

74. Gold JE, Okyay RA, Licht WE, et al. Investigation of Long COVID Prevalence and Its Relationship to Epstein-Barr Virus Reactivation. *Pathogens* 2021;10(6) doi: 10.3390/pathogens10060763 [published Online First: 2021/07/03]
75. Chen T, Song J, Liu H, et al. Positive Epstein-Barr virus detection in coronavirus disease 2019 (COVID-19) patients. *Sci Rep* 2021;11(1):10902. doi: 10.1038/s41598-021-90351-y [published Online First: 2021/05/27]
76. Mizrahi B, Sudry T, Flaks-Manov N, et al. Long covid outcomes at one year after mild SARS-CoV-2 infection: nationwide cohort study. *BMJ* 2023;380:e072529. doi: 10.1136/bmj-2022-072529 [published Online First: 2023/01/12]
77. Wang L DP, Berger NA, Kaelber DC, Volkow ND, Xu R. Disrupted seasonality and association of COVID-19 with medically attended respiratory syncytial virus infections among young children in the US: January 2010–January 2023. *MedRxiv* 2023
78. Liu Y WY, Peng Z, Li G, Wang J. T Cell Cross-reactivity in Autoimmune-like Hepatitis Triggered by COVID-19. *hLife* 2023 doi: https://doi.org/10.1016/j.hlife.2023.09.002
79. Boettler T, Csernalabics B, Salie H, et al. SARS-CoV-2 vaccination can elicit a CD8 T-cell dominant hepatitis. *J Hepatol* 2022;77(3):653-59. doi: 10.1016/j.jhep.2022.03.040 [published Online First: 2022/04/25]
80. Rela M, Jothimani D, Vij M, et al. Auto-immune hepatitis following COVID vaccination. *J Autoimmun* 2021;123:102688. doi: 10.1016/j.jaut.2021.102688 [published Online First: 2021/07/06]
81. Dowell AC, Lancaster T, Bruton R, et al. Primary Omicron infection elicits weak antibody response but robust cellular immunity in children. 2022:2022.07.26.501570. doi: 10.1101/2022.07.26.501570 %J bioRxiv
82. Xu L, Yu DD, Ma YH, et al. COVID-19-like symptoms observed in Chinese tree shrews infected with SARS-CoV-2. *Zool Res* 2020;41(5):517-26. doi: 10.24272/j.issn.2095-8137.2020.053 [published Online First: 2020/07/24]
83. Chau TN, Lee KC, Yao H, et al. SARS-associated viral hepatitis caused by a novel coronavirus: report of three cases. *Hepatology* 2004;39(2):302-10. doi: 10.1002/hep.20111 [published Online First: 2004/02/10]
84. Alsaad KO, Hajeer AH, Al Balwi M, et al. Histopathology of Middle East respiratory syndrome coronovirus (MERS-CoV) infection - clinicopathological and ultrastructural study. *Histopathology* 2018;72(3):516-24. doi: 10.1111/his.13379 [published Online First: 2017/09/01]
85. Chapin CA, Diamond T, Harris RM, et al. Adenovirus is Not Detected in Liver Tissue From a Historical Multicenter Cohort of Children With Acute Liver Failure. *J Pediatr Gastroenterol Nutr* 2023;77(3):393-95. doi: 10.1097/MPG.0000000000003851 [published Online First: 2023/05/31]
86. Martin NA, Gonzalez G, Reynolds LJ, et al. Adeno-Associated Virus 2 and Human Adenovirus F41 in Wastewater during Outbreak of Severe Acute Hepatitis in Children, Ireland. *Emerg Infect Dis* 2023;29(4):751-60. doi: 10.3201/eid2904.221878 [published Online First: 2023/03/24]
87. Mandal S, Simmons R, Ireland G, et al. Paediatric acute hepatitis of unknown aetiology: a national investigation and adenoviraemia case-control study in the UK. *Lancet Child Adolesc Health* 2023 doi: 10.1016/S2352-4642(23)00215-8 [published Online First: 2023/09/30]

88. The UK Health Security Agency (UKHSA). Increase in acute hepatitis cases of unknown aetiology in children 2022 [Available from: https://www.gov.uk/government/publications/hepatitis-increase-in-acute-cases-of-unknown-aetiology-in-children/increase-in-acute-hepatitis-cases-of-unknown-aetiology-in-children.
89. Servellita V, Sotomayor Gonzalez A, Lamson DM, et al. Adeno-associated virus type 2 in US children with acute severe hepatitis. *Nature* 2023;617(7961):574-80. doi: 10.1038/s41586-023-05949-1 [published Online First: 2023/03/31]
90. Di Dato F, Di Giorgio A, Mandato C, et al. Italian children seem to be spared from the mysterious severe acute hepatitis outbreak: A report by SIGENP Acute Hepatitis Group. *J Hepatol* 2022;77(4):1211-13. doi: 10.1016/j.jhep.2022.06.026 [published Online First: 2022/07/10]
91. Cheema HA, Shahid A, Aziz H. Severe acute pediatric hepatitis: The undue emphasis on adenovirus needs to be reassessed. *J Med Virol* 2022;94(11):5088-89. doi: 10.1002/jmv.28014 [published Online First: 2022/07/21]
92. Leruez-Ville M, Minard V, Lacaille F, et al. Real-time blood plasma polymerase chain reaction for management of disseminated adenovirus infection. *Clin Infect Dis* 2004;38(1):45-52. doi: 10.1086/380450 [published Online First: 2003/12/18]
93. Ho A OR, Tayler R, Asamaphan P, Tong L, Smollett K, Davis C. Adeno-associated virus 2 infection in children with non-A-E hepatitis. *MedRxiv* 2022
94. Jaggi P, Kajon AE, Mejias A, et al. Human adenovirus infection in Kawasaki disease: a confounding bystander? *Clin Infect Dis* 2013;56(1):58-64. doi: 10.1093/cid/cis807 [published Online First: 2012/09/27]
95. Kambhampati A, Burke R, Dietz S, et al. Trends in Acute Hepatitis of Unspecified Etiology and Adenovirus Stool Testing Results in Children — United States, 2017–2022. *MMWR Morbidity and Mortality Weekly Report* 2022;71 doi: 10.15585/mmwr.mm7124e1
96. Baker JM, Buchfellner M, Britt W, et al. Acute Hepatitis and Adenovirus Infection Among Children - Alabama, October 2021-February 2022. *MMWR Morb Mortal Wkly Rep* 2022;71(18):638-40. doi: 10.15585/mmwr.mm7118e1 [published Online First: 2022/05/06]
97. Gutierrez Sanchez LH, Shiau H, Baker JM, et al. A Case Series of Children with Acute Hepatitis and Human Adenovirus Infection. 2022 doi: 10.1056/NEJMoa2206294
98. Tacke F. Severe hepatitis outbreak in children linked to AAV2 virus. *Nature* 2023;617(7961):471-72. doi: 10.1038/d41586-023-00570-8 [published Online First: 2023/03/31]
99. Kelgeri C, Couper M, Gupte GL, et al. Clinical Spectrum of Children with Acute Hepatitis of Unknown Cause. 2022 doi: 10.1056/NEJMoa2206704
100. Hüser D, Khalid D, Lutter T, et al. High Prevalence of Infectious Adeno-associated Virus (AAV) in Human Peripheral Blood Mononuclear Cells Indicative of T Lymphocytes as Sites of AAV Persistence. *Journal of virology* 2017;91(4) doi: 10.1128/jvi.02137-16 [published Online First: 2016/12/09]
101. Radke JR, Cook JL. Human adenovirus infections: update and consideration of mechanisms of viral persistence. *Current opinion in infectious diseases* 2018;31(3):251-56. doi: 10.1097/qco.0000000000000451 [published Online First: 2018/03/31]

102. Matoq A, Salahuddin A. Acute Hepatitis and Pancytopenia in Healthy Infant with Adenovirus. *Case Rep Pediatr* 2016;2016:8648190. doi: 10.1155/2016/8648190 [published Online First: 2016/06/25]
103. Cames B, Rahier J, Burtomboy G, et al. Acute adenovirus hepatitis in liver transplant recipients. *J Pediatr* 1992;120(1):33-7. doi: 10.1016/s0022-3476(05)80593-1 [published Online First: 1992/01/01]
104. Alcamo AM, Pinchasik DE, Mo JQ, et al. Successful Treatment of Disseminated Adenovirus Infection in an Infant With Acute Lymphoblastic Leukemia. *J Pediatr Hematol Oncol* 2015;37(3):e178-81. doi: 10.1097/mph.0000000000000224 [published Online First: 2014/08/05]
105. Carmichael GP, Jr., Zahradnik JM, Moyer GH, et al. Adenovirus hepatitis in an immunosuppressed adult patient. *Am J Clin Pathol* 1979;71(3):352-5. doi: 10.1093/ajcp/71.3.352 [published Online First: 1979/03/01]
106. Cimsit B, Tichy EM, Patel SB, et al. Treatment of adenovirus hepatitis with cidofovir in a pediatric liver transplant recipient. *Pediatr Transplant* 2012;16(3):E90-3. doi: 10.1111/j.1399-3046.2010.01443.x [published Online First: 2011/01/14]
107. Hough R, Chetwood A, Sinfield R, et al. Fatal adenovirus hepatitis during standard chemotherapy for childhood acute lymphoblastic leukemia. *J Pediatr Hematol Oncol* 2005;27(2):67-72. doi: 10.1097/01.mph.0000153958.95486.6f [published Online First: 2005/02/11]
108. Kawashima N, Muramatsu H, Okuno Y, et al. Fulminant adenovirus hepatitis after hematopoietic stem cell transplant: Retrospective real-time PCR analysis for adenovirus DNA in two cases. *J Infect Chemother* 2015;21(12):857-63. doi: 10.1016/j.jiac.2015.08.018 [published Online First: 2015/10/02]
109. Mateos ME, López-Laso E, Pérez-Navero JL, et al. Successful response to cidofovir of adenovirus hepatitis during chemotherapy in a child with hepatoblastoma. *J Pediatr Hematol Oncol* 2012;34(7):e298-300. doi: 10.1097/MPH.0b013e318266ba72 [published Online First: 2012/09/01]
110. Ohbu M, Sasaki K, Okudaira M, et al. Adenovirus hepatitis in a patient with severe combined immunodeficiency. *Acta Pathol Jpn* 1987;37(4):655-64. doi: 10.1111/j.1440-1827.1987.tb00400.x [published Online First: 1987/04/01]
111. Schaberg KB, Kambham N, Sibley RK, et al. Adenovirus Hepatitis: Clinicopathologic Analysis of 12 Consecutive Cases From a Single Institution. *Am J Surg Pathol* 2017;41(6):810-19. doi: 10.1097/pas.0000000000000834 [published Online First: 2017/03/16]
112. Steiner I, Aebi C, Ridolfi Lüthy A, et al. Fatal adenovirus hepatitis during maintenance therapy for childhood acute lymphoblastic leukemia. *Pediatr Blood Cancer* 2008;50(3):647-9. doi: 10.1002/pbc.21120 [published Online First: 2007/02/06]
113. Varki NM, Bhuta S, Drake T, et al. Adenovirus hepatitis in two successive liver transplants in a child. *Arch Pathol Lab Med* 1990;114(1):106-9. [published Online First: 1990/01/01]
114. Bertzbach LD, Ip WH, Dobner T. Animal Models in Human Adenovirus Research. *Biology (Basel)* 2021;10(12) doi: 10.3390/biology10121253 [published Online First: 2021/12/25]
115. Calcedo R, Morizono H, Wang L, et al. Adeno-associated virus antibody profiles in newborns, children, and adolescents. *Clin Vaccine Immunol* 2011;18(9):1586-8. doi: 10.1128/CVI.05107-11 [published Online First: 2011/07/22]

116. Chowdary P, Nathwani A. Phase 1-2 Trial of AAVS3 Gene Therapy in Patients with Hemophilia B. Reply. *N Engl J Med* 2022;387(14):1336-37. doi: 10.1056/NEJMc2210918 [published Online First: 2022/10/06]
117. Chand D, Mohr F, McMillan H, et al. Hepatotoxicity following administration of onasemnogene abeparvovec (AVXS-101) for the treatment of spinal muscular atrophy. *J Hepatol* 2021;74(3):560-66. doi: 10.1016/j.jhep.2020.11.001 [published Online First: 2020/11/14]
118. Mullard A. Gene therapy community grapples with toxicity issues, as pipeline matures. *Nat Rev Drug Discov* 2021;20(11):804-05. doi: 10.1038/d41573-021-00164-x [published Online First: 2021/10/03]
119. Naso MF, Tomkowicz B, Perry WL, 3rd, et al. Adeno-Associated Virus (AAV) as a Vector for Gene Therapy. *BioDrugs : clinical immunotherapeutics, biopharmaceuticals and gene therapy* 2017;31(4):317-34. doi: 10.1007/s40259-017-0234-5 [published Online First: 2017/07/03]
