# Supplementary material for: Acute hepatitis of unknown aetiology in children: evidence for and against causal relationships with SARS-CoV-2, HAdv and AAV2
Source: BMJ Paediatr Open. 2024 Dec 9;8(1):e002410. doi: 10.1136/bmjpo-2023-002410 (PMC11628968; doi:10.1136/bmjpo-2023-002410)
Supplement: Supplementary file 3 [file bmjpo-2023-002410.reviewer_comments.pdf]

PEER REVIEW HISTORY

BMJ Paediatrics Open publishes all reviews undertaken for accepted manuscripts. Reviewers are asked to complete a checklist review form and are provided with free text boxes to elaborate on their assessment. These free text comments are reproduced below.

ARTICLE DETAILS

**TITLE (PROVISIONAL)**	Acute hepatitis of unknown aetiology in children – evidence for and against causal relationships with SARS-CoV-2, hAdv and AAV2
**AUTHORS**	Gurdasani, Deepti; Trent, Mallory; Ziauddeen, Hisham; Mnatzaganian, Emmanuel; Turville, Stuart; Chen, Xin; Kunasekaran, Mohana Priya; Chughtai, Abrar Ahmad; Moa, Aye; McEniery, Julie; Greenhalgh, Trisha; MacIntyre, Chandini Raina

VERSION 1 - REVIEW

**REVIEWER NAME**	***Dr. Suneel Mohammed***
**REVIEWER AFFILIATION**	UNC Health Blue Ridge
**REVIEWER CONFLICT OF INTEREST**	
**DATE REVIEW RETURNED**	

**GENERAL COMMENTS**	Comments to the Author<br>The manuscript reviews the available studies and data on the relationship between SARS-CoV-2 infection, Adenovirus infection and severe acute hepatitis in children. The Bradford Hill criteria for causality were used. It convincingly concludes that SARS-CoV-2 infection may be related to the severe acute hepatitis after a lag of approximately 16 weeks. But the relationship with Adenovirus infection is not significant but I feel that data shows that it may be significant. The studies reviewed and the methodology makes this argument convincing. I would recommend that this manuscript be accepted for publication but there are a few concerns.<br><br>The models for the severe acute hepatitis seen postulate a post infection effect. The effect of HAdV after a lag of 12-16 weeks is significant as shown in supplementary figure 4 a and 4 b. I would suggest that supplementary figure 4 a be shown in the main article and further discussion is needed to explain this.<br><br>The discussion mentions that the interaction between the two exposures, i.e. SARS-CoV-2 and HAdv could not be explored. The severe acute hepatitis seen is thought to be due to the combined effect of these two interactions. Would it be possible to combine the relationship between SARS-CoV-2 positivity, fecal Adv cases with excess hepatitis? Would we be able to combine Figure 3 a and figure 3c (or even supplementary figure 3d, e and f) to come to any meaningful conclusion?<br><br>In table 3 looking for Bradford criteria for HAdV as a causal agent:<br>For consistency I would grade it a partially met since HAdV infection was seen in most reports.<br>For temporality I would grade it partially or completely met since the association with a lag of 12- 16 weeks is significant.

	For plausibility I would grade a partially of fully met since the HLA DRB1*04:01 allele is associated with type I autoimmune hepatitis. An autoimmune hepatitis is response to HAdv41 triggered S-adenosylhomocysteine hydrolase autoantibody can trigger an autoimmune hepatitis.<br>I would suggest that the authors revise the Bradford criteria since the data is stronger.<br><br>The word “fortnight” is not used in the USA and may confuse readers. I suggest using “weeks” instead.

**REVIEWER NAME**	***Anthony Griffiths***
**REVIEWER AFFILIATION**	Boston University
**REVIEWER CONFLICT OF INTEREST**	
**DATE REVIEW RETURNED**	

**GENERAL COMMENTS**	Comments to the Author<br>Gurdasani et al, perform analyses on published literate and novel collected data to evaluate the relationship between pediatric acute severe hepatitis and SARS-CoV-2, adenovirus, and AAV-2.<br><br>Starting in late 2021, there have been reports globally of acute severe pediatric hepatitis. There was a cluster of cases in the UK of children under 5 years, that peaked in late April 2022 and has since declined. In combination with an analysis of the published data (89 papers analyzed by Bradford Hill criteria), routinely collected emergency department data were interrogated.<br><br>The authors found a strong link with SARS-CoV-2 positivity and the increase in acute severe hepatitis, which suggested that the hepatitis may be a delayed complication of SARS-CoV-2 infection. In contrast, they determined that adenovirus is unlikely to be responsible.<br><br>Overall, these are important questions that are addressed with appropriate tools and the conclusions seem valid. However, very recent data published March 29 (https://doi.org/10.1038/s41586-023-05949-1) in Nature reach the conclusion that pediatric acute severe hepatitis is related to co-infections involving AAV2 and one or more viruses. It is crucial that the authors address this recent report in their manuscript.<br><br>Dr. Daniela Silva-Ayala, a postdoctoral associate in my group with expertise in SARS-CoV-2 infection and disease contributed to this review.<br><br>Minor comments<br>• Introduce acronyms<br>○ P2 l 35 A&E<br>○ P5 l 23 ONS<br>• Figure 3c/d state in the legend that the lags are 14 weeks.<br><br><br>Major comments

	• Please address the Nature paper described above, describing if those data do or do not fit with the observations presented in this manuscript.

**REVIEWER NAME**	***Dr. Zoë Hyde***
**REVIEWER AFFILIATION**	The University of Western Australia
**REVIEWER CONFLICT OF INTEREST**	
**DATE REVIEW RETURNED**	

**GENERAL COMMENTS**	Comments to the Author<br>This study by Gurdasani and colleagues explores the evidence for a causal role for adenovirus, adeno-associated virus 2 (AAV-2), and SARS-CoV-2 in the global unexplained paediatric hepatitis outbreak. They model associations between detection of these viruses and excess emergency department presentations in the United Kingdom, and also assess the international scholarly literature using the Bradford Hill Criteria. This is a rigorous and sophisticated approach to addressing this question that surprisingly has not been undertaken to date. Their study therefore makes a novel and major contribution to our understanding of the outbreak. Accurately determining its aetiology is of crucial importance for both treatment and prevention of further cases. The manuscript is well-written and the approach sound, but there are nonetheless some issues that require clarification and/or revision by the authors.<br><br>Major comments<br><br>1. For the modelling component of this study, the authors do not appear to have had access to the raw data for emergency department presentations and adenovirus tests and have instead used a tool (WebPlotDigitizer) to extract these data from images that appear in UK Health Security Agency reports. How accurate are the data produced by this tool?<br><br>2. Three major new papers on the paediatric hepatitis outbreak have just been published in Nature:<br><br>Morfopoulou, S. et al. Genomic investigations of unexplained acute hepatitis in children (https://www.nature.com/articles/s41586-023-06003-w)<br>Ho, A. et al. Adeno-associated virus 2 infection in children with non-A-E hepatitis (https://www.nature.com/articles/s41586-023-05948-2)<br>Servellita, V. et al. Adeno-associated virus type 2 in US children with acute severe hepatitis (https://www.nature.com/articles/s41586-023-05949-1)<br><br>Two of these were cited in their pre-print form by the authors of this manuscript. However, new data appear in the final published version of at least one of the studies, and so the authors should re-read these studies and update their appraisal of the evidence where necessary.<br><br>3. Owing to new published data (point 2 above), I don't think the authors have appraised all relevant evidence for the biological gradient criterion for adenovirus and AAV-2 (Table 3). Morfopoulou et al. found high levels of AAV-2 DNA in liver, blood, plasma, or stool from 27 of 28 cases. Although it is true that this could

	represent viral reactivation (as noted by Gurdasani et al.), Morfopoulou et al. could only find AAV-2 in 6 of 17 controls (and at lower CT levels), even though the controls were immunocompromised (most had received stem cell or organ transplants). I therefore think the authors need to reappraise the evidence for this criterion and consider whether the criterion is now at least partially met.<br><br>4. For the plausibility criterion (Table 3), the authors write: “Another explanation proposed is altered immune function, either related to previous COVID-19 infection or lack of previous adenovirus exposure because of lockdowns, but there is no evidence to support either of these possibilities, and no evidence that adenovirus positivity increased above previous years in England during this period.”<br><br>There is now considerable evidence that SARS-CoV-2 infection may result in at least transitory perturbations of the immune system. I therefore don’t think the first part of the authors’ statement is justified, and I think the manuscript requires a brief discussion of how SARS-CoV-2 infection might subsequently alter the impact of other pathogens.<br><br>5. For the Analogy criterion (Table 3), the authors write: “However, diagnosis has depended on cases showing evidence of direct viral infection of the liver, which has not been observed in any explanted livers/biopsies in the current cluster to date 89”<br><br>a) The final published version of the study by Ho et al. now shows evidence of replicating AAV-2 within the nucleus and cytoplasm of hepatocytes. This new evidence should be acknowledged by the authors.<br><br>b) The authors refer the reader to reference 89 here, but this is a study published in 2017 which does not describe the current outbreak. The authors should check that all cited literature is correctly numbered.<br><br>6. In the Discussion section, the authors write: “It may be that the Omicron variants predispose more to a post-infectious hepatitis than previous variants, or simply that the scale of infection was so much greater in 2022 that the epidemic of hepatitis was more noticeable.” A problem with this hypothesis is that hepatitis cases were not seen in Australia (at least up until May 2022). Australia pursued an elimination policy for much of the pandemic and few children were infected until the omicron waves of 2022. But by August 2022, serology data suggested that 63-65% of children and adolescents had been infected. Yet no hepatitis cases had been seen by 1 May 2022: https://www.health.vic.gov.au/health-advisories/acute-hepatitis-of-unknown-cause-in-children . If SARS-CoV-2 is the causal agent, this suggests something different from a MIS-C-like phenomenon. Could multiple infections (i.e., SARS-CoV-2 reinfection) be required? I think the authors should explore this a bit more.<br><br>7. Ho et al. and Servellita et al. suggest that the lifting of non-pharmaceutical interventions deployed in 2020 may have created a situation where children were exposed to HAdV and AAV-2 at the same time, for the first time, and that these synchronised waves of infection may explain the hepatitis cases. I find this hypothesis

	unconvincing, because at least 12 hepatitis cases have been reported by Sweden, which did not close its primary schools. Nonetheless, many people consider the co-infection hypothesis plausible and so the authors of this manuscript should comment on it.<br><br>8. Further to the point above, I think it would be useful for the authors to include recent data for Australia and New Zealand (if available) in their appraisal of the evidence. Because these countries pursued an elimination policy for much of the pandemic, this essentially provides a natural experiment to test the authors' hypotheses. If there have still been no hepatitis cases detected, this would cast doubt on a role for SARS-CoV-2.<br><br>Minor points<br><br>9. When discussing seroconversion after SARS-CoV-2 infection, the authors may wish to cite new research from the Royal Children's Hospital in Melbourne that shows that compared to adults, children are half as likely to seroconvert: https://jamanetwork.com/journals/jamanetworkopen/fullarticle/2789845<br><br>10. The footnotes for Table 1 should clearly indicate the number of emergency departments that were excluded (and ultimately included) in the analyses.<br><br>11. At least one reference (Ho et al.) is duplicated in the reference list.<br><br>12. The manuscript should probably include a statement indicating that ethical approval was not required for the study.<br><br>13. There are some minor typographical errors in the manuscript.<br><br>a) Affiliations section: "Freemantle".<br><br>b) Second paragraph of the Results section: "Summary scores for based on".<br><br>c) The footnote for Figure 3 appears to be cut-off: "Figure 3d shows the linear relationship modelled between the number of faecal specimens positive for adenovirus and excess hepatitis consolidated across"<br><br>d) In the specificity criterion for SARS-CoV-2, the authors write: "Adenovirus specimen positives also increased during early 2020 …" Did they mean to write 2022 here?<br><br>e) Discussion section: "We find a significant association between excess hepatitis the number of Adv positive faecal samples".<br><br>f) Table 3: "Some possible explanations that have been HAdvanced".

**REVIEWER NAME**	***Dr. Neil Davies***
**REVIEWER AFFILIATION**	University of Bristol
**REVIEWER CONFLICT OF INTEREST**	

DATE REVIEW RETURNED	This study by Gurdasani and colleagues explores the evidence for a causal role for adenovirus, adeno-associated virus 2 (AAV-2), and SARS-CoV-2 in the global unexplained paediatric hepatitis outbreak. They model associations between detection of these viruses and excess emergency department presentations in the United Kingdom, and also assess the international scholarly literature using the Bradford Hill Criteria. This is a rigorous and sophisticated approach to addressing this question that surprisingly has not been undertaken to date. Their study therefore makes a novel and major contribution to our understanding of the outbreak. Accurately determining its aetiology is of crucial importance for both treatment and prevention of further cases. The manuscript is well-written and the approach sound, but there are nonetheless some issues that require clarification and/or revision by the authors.<br><br>Major comments<br><br>1. For the modelling component of this study, the authors do not appear to have had access to the raw data for emergency department presentations and adenovirus tests and have instead used a tool (WebPlotDigitizer) to extract these data from images that appear in UK Health Security Agency reports. How accurate are the data produced by this tool?<br><br>2. Three major new papers on the paediatric hepatitis outbreak have just been published in Nature:<br><br>Morfopoulou, S. et al. Genomic investigations of unexplained acute hepatitis in children (https://www.nature.com/articles/s41586-023-06003-w)<br>Ho, A. et al. Adeno-associated virus 2 infection in children with non-A-E hepatitis (https://www.nature.com/articles/s41586-023-05948-2)<br>Servellita, V. et al. Adeno-associated virus type 2 in US children with acute severe hepatitis (https://www.nature.com/articles/s41586-023-05949-1)<br><br>Two of these were cited in their pre-print form by the authors of this manuscript. However, new data appear in the final published version of at least one of the studies, and so the authors should re-read these studies and update their appraisal of the evidence where necessary.<br><br>3. Owing to new published data (point 2 above), I don't think the authors have appraised all relevant evidence for the biological gradient criterion for adenovirus and AAV-2 (Table 3). Morfopoulou et al. found high levels of AAV-2 DNA in liver, blood, plasma, or stool from 27 of 28 cases. Although it is true that this could represent viral reactivation (as noted by Gurdasani et al.), Morfopoulou et al. could only find AAV-2 in 6 of 17 controls (and at lower CT levels), even though the controls were immunocompromised (most had received stem cell or organ transplants). I therefore think the authors need to reappraise the evidence for this criterion and consider whether the criterion is now at least partially met.<br><br>4. For the plausibility criterion (Table 3), the authors write: "Another explanation proposed is altered immune function, either related to previous COVID-19 infection or lack of previous adenovirus exposure because of lockdowns, but there is no evidence to support either of these possibilities, and no evidence that adenovirus

	positivity increased above previous years in England during this period."<br><br>There is now considerable evidence that SARS-CoV-2 infection may result in at least transitory perturbations of the immune system. I therefore don't think the first part of the authors' statement is justified, and I think the manuscript requires a brief discussion of how SARS-CoV-2 infection might subsequently alter the impact of other pathogens.<br><br>5. For the Analogy criterion (Table 3), the authors write: "However, diagnosis has depended on cases showing evidence of direct viral infection of the liver, which has not been observed in any explanted livers/biopsies in the current cluster to date 89"<br><br>a) The final published version of the study by Ho et al. now shows evidence of replicating AAV-2 within the nucleus and cytoplasm of hepatocytes. This new evidence should be acknowledged by the authors.<br><br>b) The authors refer the reader to reference 89 here, but this is a study published in 2017 which does not describe the current outbreak. The authors should check that all cited literature is correctly numbered.<br><br>6. In the Discussion section, the authors write: "It may be that the Omicron variants predispose more to a post-infectious hepatitis than previous variants, or simply that the scale of infection was so much greater in 2022 that the epidemic of hepatitis was more noticeable." A problem with this hypothesis is that hepatitis cases were not seen in Australia (at least up until May 2022). Australia pursued an elimination policy for much of the pandemic and few children were infected until the omicron waves of 2022. But by August 2022, serology data suggested that 63-65% of children and adolescents had been infected. Yet no hepatitis cases had been seen by 1 May 2022: https://www.health.vic.gov.au/health-advisories/acute-hepatitis-of-unknown-cause-in-children . If SARS-CoV-2 is the causal agent, this suggests something different from a MIS-C-like phenomenon. Could multiple infections (i.e., SARS-CoV-2 reinfection) be required? I think the authors should explore this a bit more.<br><br>7. Ho et al. and Servellita et al. suggest that the lifting of non-pharmaceutical interventions deployed in 2020 may have created a situation where children were exposed to HAdV and AAV-2 at the same time, for the first time, and that these synchronised waves of infection may explain the hepatitis cases. I find this hypothesis unconvincing, because at least 12 hepatitis cases have been reported by Sweden, which did not close its primary schools. Nonetheless, many people consider the co-infection hypothesis plausible and so the authors of this manuscript should comment on it.<br><br>8. Further to the point above, I think it would be useful for the authors to include recent data for Australia and New Zealand (if available) in their appraisal of the evidence. Because these countries pursued an elimination policy for much of the pandemic, this essentially provides a natural experiment to test the authors' hypotheses. If there have still been no hepatitis cases detected, this would cast doubt on a role for SARS-CoV-2.

	Minor points<br><br>9. When discussing seroconversion after SARS-CoV-2 infection, the authors may wish to cite new research from the Royal Children's Hospital in Melbourne that shows that compared to adults, children are half as likely to seroconvert: https://jamanetwork.com/journals/jamanetworkopen/fullarticle/2789845<br><br>10. The footnotes for Table 1 should clearly indicate the number of emergency departments that were excluded (and ultimately included) in the analyses.<br><br>11. At least one reference (Ho et al.) is duplicated in the reference list.<br><br>12. The manuscript should probably include a statement indicating that ethical approval was not required for the study.<br><br>13. There are some minor typographical errors in the manuscript.<br><br>a) Affiliations section: "Freemantle".<br><br>b) Second paragraph of the Results section: "Summary scores for based on".<br><br>c) The footnote for Figure 3 appears to be cut-off: "Figure 3d shows the linear relationship modelled between the number of faecal specimens positive for adenovirus and excess hepatitis consolidated across"<br><br>d) In the specificity criterion for SARS-CoV-2, the authors write: "Adenovirus specimen positives also increased during early 2020 …" Did they mean to write 2022 here?<br><br>e) Discussion section: "We find a significant association between excess hepatitis the number of Adv positive faecal samples".<br><br>f) Table 3: "Some possible explanations that have been HAdvanced".<br><br>Reviewer: 4<br>Dr. Neil Davies, University of Bristol<br>Comments to the Author<br>This is an interesting paper, that has clearly taken a large amount of effort. It examines the link between COVID-19 and acute hepatitis in children. The hypothesis is interesting, however, I am sceptical that the data in this paper can provide a credible answer to this hypothesis.<br><br>1. Strength – the strength of the association between COVID-19 infection and acute hepatitis is very unclear. This paper is restricted to reporting summary-level time-series statistics, which makes it extremely difficult to attribute cause, or even interpret the magnitude of associations. Indeed as currently written, the authors do not even report the size of these associations and focus on the p-values instead. This makes it difficult for the reader to assess whether the strength criterion is met.

	2. Consistency – COVID-19 does not appear to result in hepatitis in children consistently. Internationally, there has not been a spike in cases, as one would expect, given the vast majority of children have been exposed to and infected with SARS-COV-2.<br>3. Specificity - the authors have presented no evidence or argument that COVID-19 is a specific cause of hepatitis. For example, Table 2 "The epidemic of hepatitis followed the first Omicron wave in all countries where it was observed", has no reference.<br>4. Temporality – the authors posit a 3-4 month lag between infection and hepatitis, which is possible, but to prove temporality, we would need more than just one wave of data; we would expect the 3-4 month lagged case rate to follow COVID-19 infections up and down. Currently, all the authors present is a single peak, making this claim impossible to assess. They provide little evidence of temporality.<br>5. Biological gradient, plausibility, coherence, experimental manipulation – the authors note that some but not all of these criteria are met.<br><br>In summary, the authors have time series data on COVID-19 and hepatitis rates in the UK and a literature review. Neither provides particularly strong evidence for the causal relationship between COVID-19 in children and acute hepatitis. It's an important question but not one that the authors' data can particularly well answer.<br><br>The Bradford-Hill criteria are useful and raise important questions. 1) Strength – how strong are the associations between COVID-19 infection and acute hepatitis? 2) Consistency, was the 3-4 month link between COVID-19 and acute hepatitis seen in other countries and in later COVID-19 waves? 3) Specificity, essentially, all children were exposed or infected with SARS-COV-2, yet only a very small number developed acute hepatitis; this means if SARS-COV-2 is a causal factor, it is extremely non-specific.<br><br>Many modern epidemiological methods have been developed to investigate exactly this sort of causal question, e.g. case only designs, instrumental variable analysis, difference in difference, and other forms of natural experiment. These may be able to provide more credible answer to these hypotheses.

VERSION 1 – AUTHOR RESPONSE

Reviewer: 1
Dr. Suneel Mohammed, UNC Health Blue Ridge
Comments to the Author
The manuscript reviews the available studies and data on the relationship between SARS-CoV-2 infection, Adenovirus infection and severe acute hepatitis in children. The Bradford Hill criteria for causality were used. It convincingly concludes that SARS-CoV-2 infection may be related to the severe acute hepatitis after a lag of approximately 16 weeks. But the relationship with Adenovirus infection is not significant but I feel that data shows that it may be significant. The studies reviewed and the methodology makes this argument convincing. I would recommend that this manuscript be accepted for publication but there are a few concerns.

The models for the severe acute hepatitis seen postulate a post infection effect. The effect of HAdV after a lag of 12-16 weeks is significant as shown in supplementary figure 4 a and 4 b. I would suggest that supplementary figure 4 a be shown in the main article and further discussion is needed to explain this.

We thank the reviewer for recommending acceptance of our manuscript with changes, and thank them for their comments. We agree there is an association of excess hepatitis with faecal adenovirus positive specimens in 1-4 year-olds, and have moved the figure (previously supplementary figure 4a and b) to the main article as Figure 4, as suggested. We note that data on HAdV faecal positivity (number of positive samples adjusted for number of tests done) was not available. As the UKHSA report suggested no clear increase in positivity of samples in England, it is necessary to exercise caution when interpreting and discussing these results due to possible testing and ascertainment bias of these faecal samples. Therefore, we have discussed these caveats and frame the interpretation of these results accordingly in the article's discussion. We also deem the Bradford Hill criteria for both strength and temporality partially met, as suggested by the reviewer in a later comment. We update this as follows in the manuscript:

"We find a significant association between excess hepatitis and the number of HAdv positive faecal samples in England, consistent with a recent study,[1] with a lag of 14 weeks; however, the fit of the model was poorer than with SARS-CoV-2 positivity. Furthermore, UKHSA data does not point to a clear increase in Adv faecal positivity across England, suggesting that case increases may potentially reflect an increase in testing."

The discussion mentions that the interaction between the two exposures, i.e. SARS-CoV-2 and HAdv could not be explored. The severe acute hepatitis seen is thought to be due to the combined effect of these two interactions. Would it be possible to combine the relationship between SARS-CoV-2 positivity, fecal Adv cases with excess hepatitis? Would we be able to combine Figure 3 a and figure 3c (or even supplementary figure 3d, e and f) to come to any meaningful conclusion?

In the discussion we mention that we were unable to examine the evidence for an interaction between SARS-CoV-2 and HAdv and/or AAV-2 infections, as this would require either studies that have explicitly examined an interaction between these factors, or routine surveillance across countries with differences in timing of peak exposures for SARS-CoV-2 and HAdv, to examine if the relative timings of exposure is associated with the number of unknown hepatitis cases. However, these data do not exist for most countries across the globe, and the omicron wave appears to have occurred at similar times across much of the world. The existing literature examining potential causal relations between the two directly is scarce. To detect an interaction, we would require studies that evaluate both exposures within the same group of patients (both cases and controls) and provide individual outcome data based on presence of both exposures vs single exposure vs none in appropriate sample sizes to detect a difference in association with outcome. To our knowledge there are no studies that have directly assessed this, although this has been hypothesised. We add to the discussion the need for exploration of this avenue in future work.

Unfortunately, superimposing figures 3a and c would not help assess causality between SARS-CoV-2 and Adv, as we do not have individual level or aggregate information on the number of children who had both Adv positivity and SARS-CoV-2 positive serology (all data are separate) during this period, so it is very difficult to compare the association with combined exposure to both agents. We also found data on hAdV surveillance globally was sparse, making it challenging to identify regions where waves of SARS-CoV-2 or hAdV may have been more displaced from each other in time, which would be another way to examine this (i.e. look for ecological associations being different in regions where these waves occurred less concurrently). Additionally, there has been very limited surveillance of AAV-2, with only one study that we are aware of examining exposure through wastewater in Ireland.[2]

We add the following text to our limitations section in the Discussion as follows:

"We were unable to examine the evidence for an interaction between the two exposures, i.e. SARS-CoV-2 leading to an increase in susceptibility to infection by HAdv and/or AAV-2, as studies assessing both of these are currently very limited, and assessment of this criteria would likely require functional studies that currently do not exist. Additionally, our statistical modelling was limited by small sample sizes, the noisiness of routine ED surveillance data, and the availability of aggregate rather than patient individually collected data."

In table 3 looking for Bradford criteria for HAdV as a causal agent:
For consistency I would grade it a partially met since HAdV infection was seen in most reports.

In light of recent evidence, we now assess the evidence for both hAdV and AAV-2 considering hAdV as a permissive virus for AAV-2. While there was lack of consistency in hAdV positivity across countries (not observed in Japan, and associations in Europe largely driven by the UK), and in case-control studies, we consider that there is greater consistency in the association with AAV-2, with positivity, and viral load consistently higher among cases compared to controls. The proportions of infected people with HAdv among cases of fulminant hepatitis are not consistent across different population worldwide. While in the UK, 64% of those tested for HAdv were positive, only 35% of tested cases in Europe were positive for HAdv, driven largely by data from the UK, with positivity being relatively low when these data are removed[1]. Furthermore, in Japan only 9% of unknown cause hepatitis cases tested were positive for HAdv[2]. A recent study from Scotland showed only a third of hepatitis cases were positive for HAdv, with very low viral loads, which were not different from control samples.[3] Another study from Italy showed only 6 of 26 patients with paediatric unknown hepatitis in a case-series from Italy were positive for hAdv.[4] In studies where HAdv was present and subtypes were measured, the strains were not the same, even though HAdv 41F predominated. However, HAdv appears to be more consistently seen in cases in studies within the UK and US, as outlined in Table 3. As suggested by the reviewer, we score the criterion for HAdv as "partially met".

We also separately assess consistency for AAV-2 which may be more consistently observed among cases, at least in the limited studies (UK, US) that have assessed this. As others have hypothesised, the link between HAdv and fulminant hepatitis may not be a direct one, but rather that AAV-2 requires a permissive virus for infection, and hAdv may be one of the permissive viruses for this (among others such as HHV-6, which have been detected at similar levels in cases). We therefore deem this criteria also "partially met" for AAV-2.

We update this in Table 3, adding recent evidence on hAdV, and AAV-2, and summarising the criterion as follows:

"This criterion is partially met for the association between AAV-2 and hepatitis."

For temporality I would grade it partially or completely met since the association with a lag of 12- 16 weeks is significant.

We thank the reviewer for this suggestion. We have now updated the scores accordingly to "partially met". In our study, we try to capture the association between infection rates and fulminant hepatitis over time. However, as true infection rates cannot be ascertained when testing is not community-wide, we use trends in positivity as a proxy for trends in true infection rates. This approach has been used extensively in the study of infectious disease associations. Using positivity, rather than case numbers adjusts for changes in case ascertainment (e.g. increased testing). While we agree there is a significant association of faecal positive specimens and excess A&E attendances for liver conditions, the increase in faecal specimens positive appears to be potentially driven by more testing in the UK, rather than an actual increase in infection rate, as there is no clear increase in positivity (proportion of tests positive), where the data were collected by the UKHSA. Therefore, increase in number of faecal specimens positive appears to be driven by increased ascertainment. We could not test this directly due to lack of weekly data on faecal positivity. By contrast, the respiratory adenovirus positivity rate, which was available did also does not appear to have increased in the UK in under 5 year-olds (other than an increase in children under five observed in Wales between January and May 2022 that subsequently decreased to pre-pandemic levels), and shows no association with fulminant hepatitis[3]. We note that the pattern of increase in faecal samples positive for HAdv was the same as that for respiratory samples, where when positivity was assessed, no increases was seen. Additionally, a study found that since October 2021, the positivity for adenovirus types 40/41 among children in the US have not increased when compared to pre-pandemic levels[6]. However, since we did find an association with

number of samples, with caveats around increased ascertainment, we now change the scoring after taking into account our analysis results, and the fact that weekly faecal positivity levels were not available, and update this critera to “partially met”.

As we have also updated our assessment to include AAV-2 as well, we also consider this within these criteria. While we do not have data on the population level of AAV-2 exposure, given this is difficult to measure and not ordinarily ascertained, we consider the evidence from limited studies of hepatitis cases. One study showed enrichment of IgM antibodies to AAV-2 among cases compared with controls, providing evidence of recent infection with AAV-2 (rather than re-activation). Taking collective evidence on hAdv and AAV-2 into account, we now consider this criterion “partially met” for both, and update this in the manuscript accordingly.

For plausibility I would grade a partially of fully met since the HLA DRB1*04:01 allele is associated with type I autoimmune hepatitis. An autoimmune hepatitis is response to HAdv41 triggered S-adenosylhomocysteine hydrolase autoantibody can trigger an autoimmune hepatitis.
I would suggest that the authors revise the Bradford criteria since the data is stronger.

Thank you for bringing this to our attention. After reviewing the meta-analysis by Shome et al.[7], we agree that similarities between peptide sequences of the human enzyme AHCY and a viral protein of HAdV-41 have been found, potentially leading to the production of autoantibodies following HAdV-41 infection. After considering more reports, we agree with the reviewer and have changed the score of the plausibility criterion to ‘partially met’. We also include this in the manuscript, under Table 4 in the Biological Plausibility section as follows:

“A bioinformatics meta-analysis found similarities between peptide sequences of the human enzyme AHCY and a viral protein of HAdV-41[94], potentially leading to the production of autoantibodies following HAdV-41 infection. Such autoantibodies may be part of an autoimmune acute hepatitis pathway”

The word “fortnight” is not used in the USA and may confuse readers. I suggest using “weeks” instead.

We have revised the manuscript to reflect this.

Reviewer: 2
Dr. Anthony Griffiths, Boston University
Comments to the Author
Gurdasani et al, perform analyses on published literate and novel collected data to evaluate the relationship between pediatric acute severe hepatitis and SARS-CoV-2, adenovirus, and AAV-2.

Starting in late 2021, there have been reports globally of acute severe pediatric hepatitis. There was a cluster of cases in the UK of children under 5 years, that peaked in late April 2022 and has since declined. In combination with an analysis of the published data (89 papers analyzed by Bradford Hill criteria), routinely collected emergency department data were interrogated.

The authors found a strong link with SARS-CoV-2 positivity and the increase in acute severe hepatitis, which suggested that the hepatitis may be a delayed complication of SARS-CoV-2 infection. In contrast, they determined that adenovirus is unlikely to be responsible.

Overall, these are important questions that are addressed with appropriate tools and the conclusions seem valid. However, very recent data published March 29 (https://doi.org/10.1038/s41586-023-05949-1) in Nature reach the conclusion that pediatric acute severe hepatitis is related to co-infections involving AAV2 and one or more

viruses. It is crucial that the authors address this recent report in their manuscript.

Dr. Daniela Silva-Ayala, a postdoctoral associate in my group with expertise in SARS-CoV-2 infection and disease contributed to this review.

We thank the reviewer for pointing to this new study published during review. We have updated the evidence since the submission, including the paper mentioned above, and revised our evidence for various criteria accordingly, including for evidence in favour of an AAV-2 causal association.

Minor comments

- **Introduce acronyms**
  - **P2 l 35 A&E**
  - **P5 l 23 ONS**
- **Figure 3c/d state in the legend that the lags are 14 weeks.**

We thank the reviewer for their comments. We accept these comments and have introduced the acronyms A&E (Accident & Emergency) and ONS (Office for National Statistics) on page 2 and 5 respectively. Furthermore, we have added into figure 3's legend that subfigures c and d related to a lag of 14 weeks.

Major comments

- **Please address the Nature paper described above, describing if those data do or do not fit with the observations presented in this manuscript.**

Thank you for pointing out this newly published paper – have incorporated the findings of this study, and other studies published since our original submission into our manuscript.

Reviewer: 3
Dr. Zoë Hyde, The University of Western Australia
Comments to the Author
This study by Gurdasani and colleagues explores the evidence for a causal role for adenovirus, adeno-associated virus 2 (AAV-2), and SARS-CoV-2 in the global unexplained paediatric hepatitis outbreak. They model associations between detection of these viruses and excess emergency department presentations in the United Kingdom, and also assess the international scholarly literature using the Bradford Hill Criteria. This is a rigorous and sophisticated approach to addressing this question that surprisingly has not been undertaken to date. Their study therefore makes a novel and major contribution to our understanding of the outbreak. Accurately determining its aetiology is of crucial importance for both treatment and prevention of further cases. The manuscript is well-written and the approach sound, but there are nonetheless some issues that require clarification and/or revision by the authors.

Major comments

1. For the modelling component of this study, the authors do not appear to have had access to the raw data for emergency department presentations and adenovirus tests and have instead used a tool (WebPlotDigitizer) to extract these data from images that appear in UK Health Security Agency reports. How accurate are the data produced by this tool?

We thank the reviewer for their comments. Indeed, we did not have direct access to the raw data for ED presentations for children aged 1-4 with 'liver condition', nor to the raw data of adenovirus tests. WebPlotDigitizer has been validated extensively, as discussed previously.[5] We include a sentence in the methods section to highlight this, as follows:

"Data for the period 1 April 2018 to 3 July 2022 (Figure 16A in technical report 4)[6] (Figure 1) were extracted using WebPlotDigitizer (https://automeris.io/WebPlotDigitizer/citation.html), which has been shown to be a reliable and valid tool for extraction of numerical data from plots.[5]"

2. Three major new papers on the paediatric hepatitis outbreak have just been published in Nature:

Morfopoulou, S. et al. Genomic investigations of unexplained acute hepatitis in children (https://www.nature.com/articles/s41586-023-06003-w)
Ho, A. et al. Adeno-associated virus 2 infection in children with non-A-E hepatitis (https://www.nature.com/articles/s41586-023-05948-2)
Servellita, V. et al. Adeno-associated virus type 2 in US children with acute severe hepatitis (https://www.nature.com/articles/s41586-023-05949-1)

Two of these were cited in their pre-print form by the authors of this manuscript. However, new data appear in the final published version of at least one of the studies, and so the authors should re-read these studies and update their appraisal of the evidence where necessary.

We thank the reviewer for pointing us to these newly published papers. We have updated our review with these published articles, and updated Bradford hill criteria with respect to evidence for hAdv and AAV-2 accordingly. We find that these strengthen evidence for several criteria, specifically with respect to evidence for the AAV-2 association with paediatric hepatitis. We have substantially revised Tables 2 and 3, added Table 4, and revised all the text in the manuscript to reflect this.

3. Owing to new published data (point 2 above), I don't think the authors have appraised all relevant evidence for the biological gradient criterion for adenovirus and AAV-2 (Table 3). Morfopoulou et al. found high levels of AAV-2 DNA in liver, blood, plasma, or stool from 27 of 28 cases. Although it is true that this could represent viral reactivation (as noted by Gurdasani et al.), Morfopoulou et al. could only find AAV-2 in 6 of 17 controls (and at lower CT levels), even though the controls were immunocompromised (most had received stem cell or organ transplants). I therefore think the authors need to reappraise the evidence for this criterion and consider whether the criterion is now at least partially met.

We agree with the reviewer that Morfopoulou et al shows evidence of a biological gradient, with not just a higher proportion of cases being positive for AAV-2 compared to immunocompromised controls, but also higher viral loads (lower Ct threshold) among cases compared with controls. We note that immunocompromised controls had higher viral loads than healthy controls, indicating evidence of possible activation. However, we agree that cases had even higher levels, providing evidence supporting a biological gradient. We update our assessment of this criteria to partially met, as suggested.

We, however, note a significant limitation of the design, where immunocompromised controls were not age-matched, with, with a median age of 1 yr compared to 3 yrs in cases. This is important because age has been shown to be strongly associated with AAV-2 seroprevalence, with seroprevalence being lowest in under 1 year olds[7] (as 12 out of 17 controls were in this study), which could potentially lead to a false positive association here.

We discuss these results in the context of this limitation in Table 4 and in our discussion.
"*27 of 28 cases tested were AAV2 PCR positive and 6 of 17 (35%) of immunocompromised controls were positive for AAV2, significantly fewer than in cases and at significantly lower Ct levels.*[8]"

"*Several studies had limitations with respect to controls:*
Controls were either not contemporaneous, or when contemporaneous, not immunocompromised in several studies, making the groups hard to compare, as AAV-2 can re-activate with immunosuppression, and exposure has varied considerably over time. Only one immunocompetent individual and 17 immunocompromised controls were sampled contemporaneously during the outbreak in one study.[8] *However, in this study controls were not*

age matched to cases, with a median age of 1 compared to 3 in cases. Age has been shown to be strongly associated with AAV-2 seroprevalence, with seroprevalence being lowest in under 1 year olds[7] (as 12 out of 17 controls were), which could potentially lead to a false positive association here."

Discussion:
"*AAV-2 has not been known to cause disease before, but it is also possible that a unique combination predisposing factors (e.g. co-infection, or genetic predisposition, or increased susceptibility post-COVID, or very high exposure) led to this rare manifestation in children. We also note that AAV-2 activation due to severe fulminant hepatitis cannot be ruled out, as there was a dirth of studies that included age-matched contemporaneous immunosuppressed controls, which would be needed to assess the role of immunosuppression in fulminant hepatitis in AAV-2 re-activation. In the only study that did include immunosuppressed controls, the age of controls was lower than cases, with the vast majority below 1 year,[8] a group known to have the lowest seroprevalence of AAV-2, and thus likely to bias associations away from the null*."

4. For the plausibility criterion (Table 3), the authors write: "Another explanation proposed is altered immune function, either related to previous COVID-19 infection or lack of previous adenovirus exposure because of lockdowns, but there is no evidence to support either of these possibilities, and no evidence that adenovirus positivity increased above previous years in England during this period."

There is now considerable evidence that SARS-CoV-2 infection may result in at least transitory perturbations of the immune system. I therefore don't think the first part of the authors' statement is justified, and I think the manuscript requires a brief discussion of how SARS-CoV-2 infection might subsequently alter the impact of other pathogens.

We agree that more evidence has emerged that SARS-CoV-2 leads to dysregulation of the immune system, and has been linked to virus re-activation, and potentially an increased risk of infections post-COVID. We explore these avenues in Table 3, as follows:

"*SARS-CoV-2 has been associated with immune dysregulation, including activation of latent viruses (e.g. EBV[9]). It is plausible that past infection may activate latent viruses (like HAdv and HHV-6, and AAV-2). It has also been associated with increased risk of infection several months post-COVID, including streptococcal tonsillitis[10] and RSV infection.[11] It is plausible that it may have increased the risk of infection with other permissive viruses leading to AAV-2 infection/activation."*

We also discuss this in the discussion, as follows:

"We note that SARS-CoV-2 has been associated with immune dysregulation, including activation of latent viruses (e.g. EBV[9]), and increased risk of infection several months post-COVID. [10,11] It is plausible that past infection may activate latent viruses (like HAdv and HHV-6, and AAV-2), or that SARS-CoV-2 superantigens may sensitise the host to other viral infections (e.g. AAV-2) and that hepatitis of unknown cause may reflect an interaction between the two agents.[12,13] "

5. For the Analogy criterion (Table 3), the authors write: "However, diagnosis has depended on cases showing evidence of direct viral infection of the liver, which has not been observed in any explanted livers/biopsies in the current cluster to date 89"

a) The final published version of the study by Ho et al. now shows evidence of replicating AAV-2 within the nucleus and cytoplasm of hepatocytes. This new evidence should be acknowledged by the authors.

b) The authors refer the reader to reference 89 here, but this is a study published in 2017 which does not describe the current outbreak. The authors should check that all cited literature is correctly numbered.

We agree with the reviewer – we have now updated our manuscript to articles published since, and included findings by Ho et al. in Table 3, and updated our assessment of criteria accordingly.

6. In the Discussion section, the authors write: "It may be that the Omicron variants predispose more to a post-infectious hepatitis than previous variants, or simply that the scale of infection was so much greater in 2022 that the epidemic of hepatitis was more noticeable." A problem with this hypothesis is that hepatitis cases were not seen in Australia (at least up until May 2022). Australia pursued an elimination policy for much of the pandemic and few children were infected until the omicron waves of 2022. But by August 2022, serology data suggested that 63-65% of children and adolescents had been infected. Yet no hepatitis cases had been seen by 1 May 2022: https://www.health.vic.gov.au/health-advisories/acute-hepatitis-of-unknown-cause-in-children . If SARS-CoV-2 is the causal agent, this suggests something different from a MIS-C-like phenomenon. Could multiple infections (i.e., SARS-CoV-2 reinfection) be required? I think the authors should explore this a bit more.

We note that if hepatitis of unknown cause is related to SARS-CoV-2, it is a very rare outcome. Our examination of the strength of association showed only a 0.38 (95% CI 0.19-0.57) predicted overall increase in excess A&E admissions for a 1% increase in SARS-CoV-2 positivity within England. This represents a very small increase. The extent to which an increase like this would be picked up depends on the the strength of surveillance, and the period over which children were exposed (if they were exposed more slowly this would be harder to pick up). There is also a baseline rate of hepatitis of unknown cause that is known to occur.[14] This represents up to 40% of hepatitis cases, as discussed previously.[14] In Australia, a previous study has shown 30% rate of undiagnosed acute liver failure in children at baseline.[15] Picking up a signal over and above this baseline is challenging, unless there is a substantial increase in cases seen. We note that even in the US where several cases of hepatitis of unknown cause in children were identified, there was no excess signal seen above the baseline of hepatitis of unknown aetiology when surveyed across the paediatric population. This signal was, however, seen in the UK, and in the Netherlands. Given the population of under 5s across Australia is 1.5 million, and exposure occurred over a few months, it is possible that an increase may not have been observed, due to the rarity of the phenotype. However, it is still odd that no cases were identified at all given there should be a baseline rate of acute liver failure of unknown cause in children, as has been described previously[15].[14,16] This lack of identification of any cases may point to lack of routine joined up surveillance, as a background rate of cases should ideally have been detected (as has been noted globally).[14]

Another plausible explanation is that SARS-CoV-2 is not causally related to paediatric hepatitis, or is part of a more complex causal pathway. If this is the case, then conditions within the causal framework may not have been met in Australia. For example, if causality is complex and involves exposure to both SARS-CoV-2 and then AAV-2 within a period of time in a genetically susceptible cohort, all these conditions may not have been met in different regions. While SARS-CoV-2 waves occurred consistently globally, exposure to helper viruses or AAV-2 may not have been consistent across the globe. If AAV-2 was a sole causal agent, difference in regional exposure may also explain this. It is difficult to examine this hypothesis as there is no routine surveillance for AAV-2 infection, and this has been primarily only studied in the US and UK among hepatitis cases. While hAdv infection may be used as a possible proxy, as one of the permissive viruses that allow AAV-2 replication, we were unable to find routine surveillance data for hAdv during this time period within Australia to be able to explore this further. We note that South Korea, while reporting a significant rise in paediatric enteric adenovirus infection[17] does not seem to have seen a considerable number of cases (only 1 case identified) despite surveillance for this. However, in South Korea, the omicron wave occurred later, peaking in May 2022 (rather than Jan-Feb 2022, as was seen across the UK, US and Europe) and followed the rise in enteric adenovirus (rather than a rise in adenovirus seen concurrent with or just after the omicron wave, as occurred in the UK).[17]

Another possibility, as the reviewer suggests, is whether re-infection may be necessarily to trigger this phenotype, which would explain the high levels of cases observed in the UK and US, where substantial proportion of the population was exposed even prior to the omicron wave (e.g. ~40% in the UK). We also note that very few cases were observed in some other Asia-Pacific countries as

well, such as Japan. However, it is difficult to separate out the impact of the strength of surveillance from real disparities geographically. We discuss these inconsistencies under the consistency Bradford Hill Criteria, and downgrade the consistency criteria to partially met:

"The clusters of cases of unknown hepatitis peaked in the US, UK, Spain, Italy, the Netherlands and Poland in April-May 2022, ~2-3 months following the first omicron wave in these areas. [13,18,19] *However, we also note that significant clusters of hepatitis cases were not observed in some regions despite high levels of omicron infection (e.g. Australia, New Zealand, and South Korea). This could be because cases did not consistently occur post-COVID (SARS-CoV-2 not being a sufficient condition to be causal), or poor background surveillance, or rarity of the outcomes."*

We also discuss this in the Discussion, as follows:
"We note that while the omicron wave was followed by identified paediatric hepatitis cases of unknown cause in many countries, this did not occur in all countries with large omicron waves (e.g. Australia, New Zealand); we therefore consider the consistency criteria only partially met. Whether this represents lack of ascertainment due to gaps in surveillance, or inability to pick up rare signals in smaller populations, or all causal conditions for hepatitis of unknown cause not being met in these regions is unclear. "

7. Ho et al. and Servellita et al. suggest that the lifting of non-pharmaceutical interventions deployed in 2020 may have created a situation where children were exposed to HAdV and AAV-2 at the same time, for the first time, and that these synchronised waves of infection may explain the hepatitis cases. I find this hypothesis unconvincing, because at least 12 hepatitis cases have been reported by Sweden, which did not close its primary schools. Nonetheless, many people consider the co-infection hypothesis plausible and so the authors of this manuscript should comment on it.

We thank the reviewer for this comment. We have added this into the discussion, as follows:

"*Another factor suggested that may have altered conditions are mitigation measures in 2020-2021 which may have led to concurrent surges of hAdv and AAV-2, leading to much higher levels of infection, with manifestation of a rare syndrome not identified before. However, we consider this unlikely given significant numbers of cases (12 for a population of 590,000 under 5s)*[20] *were seen even in countries like Sweden, where mitigations were limited, and there was very little suppression of seasonal influenza, unlike in many other countries where this was considerably suppressed.*[21]*"*

8. Further to the point above, I think it would be useful for the authors to include recent data for Australia and New Zealand (if available) in their appraisal of the evidence. Because these countries pursued an elimination policy for much of the pandemic, this essentially provides a natural experiment to test the authors' hypotheses. If there have still been no hepatitis cases detected, this would cast doubt on a role for SARS-CoV-2.

We thank the reviewer for this suggestion. We have since contacted the lead of the Australian study of hepatitis of unknown cause to request access to these data, but did not receive a response, so have been unable to include these. Unfortunately, these data do not appear to be publicly available. We, however, discuss the lack of identification of hepatitis cases in Australia and New Zealand within our consistency criteria, and have updated our criteria based on this, as described previously.

Minor points

9. When discussing seroconversion after SARS-CoV-2 infection, the authors may wish to cite new research from the Royal Children's Hospital in Melbourne that shows that compared to adults, children are half as likely to seroconvert:
https://jamanetwork.com/journals/jamanetworkopen/fullarticle/2789845

We thank the reviewer for pointing us to this paper. While discussing serosurveillance among children, we have added that children, despite having similar viral loads and experiencing mild COVID-19, may be less prone to seroconversion compared to adults, citing Toh et al, as follows.[9]

"*The lack of serological testing for SARS-CoV-2 in most available reports of hepatitis hindered the understanding of aetiology. Epidemiological surveillance, including serosurveillance for SARS-CoV-2 in children with hepatitis remains key to outbreak investigation and early response in the future. Nonetheless, it is essential to acknowledge that relying on SARS-CoV-2 serosurveillance among children might not be entirely reliable or highly informative. This is because children, despite having similar viral loads and experiencing mild COVID-19, may be less prone to seroconversion compared to adults*.[22]"

10. The footnotes for Table 1 should clearly indicate the number of emergency departments that were excluded (and ultimately included) in the analyses.

We have included the number of emergency departments in the footnotes of Figure 1, as reported by the UKHSA as follows:

*"** ED attendances as identified by syndromic surveillance, including 104 EDs:*
• type 01 ED attendances only
• limited to EDs which started reporting through this route during 2018
• ED syndromic surveillance reporting through the route reported here commenced April 2018
• limited to EDs which reported quickly and frequently in the most recent week (received data for 7 out of 7 of the days 27 June to 3 July 2022, and the data arrived with the UKHSA Realtime Syndromic Surveillance Team within 2 calendar days of the patient attendance)
• EDs are excluded where historical issues with diagnosis coding have been identified."

Unfortunately, we do not have the flowchart as to what EDs were included, and which were excluded, as these were not reported by the UKHSA. We have reported on emergency department attendances, exactly as reported in the UKHSA report on this, and we do not have further information regarding these.[6]

We include this among the limitations in our Discussion, as follows:

"*As we did not have the data on individual EDs included in the study, we were unable to assess the representativeness of UKHSA data across the England population."*

11. At least one reference (Ho et al.) is duplicated in the reference list.

Thank you for pointing it out – we have de-duplicated that reference.

12. The manuscript should probably include a statement indicating that ethical approval was not required for the study.

We have included a statement as follows:

"The study consisted entirely of desk research on material that was in the public domain, hence was outwith the remit of UK research ethics committees and the Health Research Authority. No ethics approval was required for the study."

13. There are some minor typographical errors in the manuscript
a) Affiliations section: "Freemantle".

Thank you for pointing us to this error – 'Freemantle' was corrected to 'Fremantle'.

b) Second paragraph of the Results section: "Summary scores for based on".

Thank you for pointing us to this error – we have corrected that sentence.

c) The footnote for Figure 3 appears to be cut-off: "Figure 3d shows the linear relationship modelled between the number of faecal specimens positive for adenovirus and excess hepatitis consolidated across"

Thank you for pointing us to this error – we have corrected that text box error.

d) In the specificity criterion for SARS-CoV-2, the authors write: "Adenovirus specimen positives also increased during early 2020 …" Did they mean to write 2022 here?

Thank you for pointing us to this error – correct, we meant '2022' and not '2020'.

e) Discussion section: "We find a significant association between excess hepatitis the number of Adv positive faecal samples".

Thank you for pointing us to this error – we have corrected that sentence.

f) Table 3: "Some possible explanations that have been HAdvanced".

Thank you for pointing us to this error – we have corrected this in the revised manuscript.

Reviewer: 4
Dr. Neil Davies, University of Bristol
Comments to the Author
This is an interesting paper, that has clearly taken a large amount of effort. It examines the link between COVID-19 and acute hepatitis in children. The hypothesis is interesting, however, I am sceptical that the data in this paper can provide a credible answer to this hypothesis.

1. Strength – the strength of the association between COVID-19 infection and acute hepatitis is very unclear. This paper is restricted to reporting summary-level time-series statistics, which makes it extremely difficult to attribute cause, or even interpret the magnitude of associations. Indeed as currently written, the authors do not even report the size of these associations and focus on the p-values instead. This makes it difficult for the reader to assess whether the strength criterion is met.

We have reported on the magnitude of associations for the exposure-outcome relationship. As indicated in our Supplementary methods, this was calculated by calculating the coefficients along the overall exposure-response curve reduced along the lag dimension. For SARS-CoV-2, we reported this on pg. of the manuscript as follows:

*"Overall, the model estimated a 0.38 (95% CI 0.19-0.57) predicted overall increase in excess A&E admissions for a 1% increase in SARS-CoV-2 positivity (**Figure 3c**)."*

The magnitude of both associations (which are themselves on different scales- positivity, and number of samples) are shown in Figure 3c and 3d, and can be compared visually. These represent the association consolidated across all lag weights. The reason we did not report the coefficients for the association with faecal samples and chose to show a figure instead was that the best model for association with number of faecal samples was non-linear polynomial model, where coefficients can be difficult to interpret. We chose to show this visually in Figure 3d, where the association can be shown to vary markedly with exposure, with the association being strongest at higher values of faecal specimen numbers, when collapsed across lags. The 3-dimensional association (non-linear by number of faecal specimens, and lags) is shown in Figure 4. As the figure shows, the association is difficult to represent with a single coefficient due to non-linearity.

2. Consistency – COVID-19 does not appear to result in hepatitis in children consistently. Internationally, there has not been a spike in cases, as one would expect, given the vast majority of children have been exposed to and infected with SARS-COV-2.

In light of re-review of more recent evidence, and comments by Reviewer 2 and 3, we agree that the criterion for consistency for SARS-CoV-2 exposure are only partially met. However, we also note that given hepatitis appears to be a rare outcome, and surveillance differs markedly across countries, it may have been difficult to pick up a signal for this in places where surveillance is more limited. Even prior to the hepatitis cluster, a systematic review showed that 40% of all cases of paediatric acute liver failure had undetermined cause across many developed countries, indicating there is a baseline rate of hepatitis of unknown cause in children.[14] For example, a study from the US did not show an increase in paediatric hepatitis-associated emergency department visits during 2022, hospitalisations due to hepatitis or liver transplants in children, although trends and epidemiology of cases of unknown hepatitis was very similar to those observed within the UK. This suggests that given there is an existing baseline of hepatitis of unknown cause in every country, the signal may not have been obvious without specific surveillance systems, or a large increase in numbers in a short period of time.[19] In the UK, we were able to pick this up in excess of existing A&E attendances for liver conditions, data that have not been made available from other countries. This indicates that fulminant hepatitis of unknown cause as a signal may be observed above a baseline only when the cluster is large, and occurs in a short period of time, and surveillance systems are good. Given this is likely to be a very rare occurrence potentially associated with SARS-CoV-2 exposure, the level of exposure in a short period of time would make a difference. In this context, the UK had ~40-50% children exposed over a period of just 3 months, which is a very high level of exposure in a very short period of time. Most countries across the world did not have this level of exposure during the same period because of greater mitigations in place. With lower level exposure over a longer period, an excess of cases would have been harder to find, and highly dependent on routine surveillance carried out among this age group. We were unable to find equivalent surveillance from other countries, which makes the criterion of consistency much harder to assess.

3. Specificity - the authors have presented no evidence or argument that COVID-19 is a specific cause of hepatitis. For example, Table 2 "The epidemic of hepatitis followed the first Omicron wave in all countries where it was observed", has no reference.

We have added the references for, and rephrased as follows:
"The clusters of cases of unknown hepatitis peaked in the US, UK, Spain, Italy, the Netherlands and Poland[13,18,19] in April-May 2022, ~2-3 months following the first omicron wave in these areas."

4. Temporality – the authors posit a 3-4 month lag between infection and hepatitis, which is possible, but to prove temporality, we would need more than just one wave of data; we would expect the 3-4 month lagged case rate to follow COVID-19 infections up and down. Currently, all the authors present is a single peak, making this claim impossible to assess. They provide little evidence of temporality.

We agree that ideally to see temporality, we need multiple waves. However, given the nature of infectious disease, and the development of immunity, these are often not seen once the majority of the population is exposed. We use the example of MIS-C to illustrate this. While MIS-C was prominently observed as a rare complication of COVID-19 in 2020-2021, the incidence of MIS-C has reduced substantially since, as surveillance from several countries across the globe has shown.[22-24] This is very likely because the majority of children have now been exposure in most countries, and potentially re-infection is less likely to result in this complication due to pre-existing immunity. Given there have been no additional waves of hepatitis of unknown cause, we are unable to examine this beyond a single wave. We note that the same pattern has been observed for MIS-C.

5. Biological gradient, plausibility, coherence, experimental manipulation – the authors note that some but not all of these criteria are met.

In summary, the authors have time series data on COVID-19 and hepatitis rates in the UK and a literature review. Neither provides particularly strong evidence for the causal relationship between COVID-19 in children and acute hepatitis. It's an important question but not one that the authors' data can particularly well answer.

We agree that we use aggregate data to look at temporal and strength relationships and review the literature to try to understand causality within the Bradford hill criteria framework. These data are limited, as are the inferences we can make from the studies done so far. We note that similar methods have been applied in previous studies examining the temporal association between adenovirus and hepatitis cases.[1] However, we believe it is important to highlight the information we have so far, even if the inferences are not complete, as this provides a comprehensive summary of the evidence so far, and highlights avenues where further research is needed. We also note the recent genetic association of cases with the HLA DRB 0401 allele, an allele also shown to be associated with COVID asymptomaticity. This allele lies on a haplotype that has been associated with asymptomaticity in COVID-19, attributed in functional studies to possible T-cell cross-reactivity against SARS-CoV-2. It is plausible that a stronger T-cell response against SARS-CoV-2 may be associated with a post-inflammatory condition. This provides another avenue for future work. To our knowledge the association of the same allele with both phenotypes, and its implications has not been reported on before.

We note in our limitations and conclusions that we cannot prove causality, as follows:
"*There were limitations to this study. We reviewed current observational evidence and scored criteria for causality and the evidence supporting each to assess this within a causal framework. Given our reliance on limited observational data, we are unable to prove causality. Limitations of studies include biases in hepatitis case ascertainment, small number of studies, small sample sizes and with inadequate matching where case-control analysis was done. In addition, lack of routine COVID testing and absence of complete serological data across countries, were a limitation. Furthermore, as we did not have the data on individual EDs included in the study, we were unable to assess the representativeness of UKHSA data across the England population. We were also unable to examine the evidence for an interaction between the two exposures, i.e. SARS-CoV-2 leading to an increase in susceptibility to infection by HAdv and/or AAV-2, as studies assessing both of these are currently very limited, and assessment of this criteria would likely require functional studies that currently do not exist. Additionally, our statistical modelling was limited by small sample sizes, the noisiness of routine ED surveillance data, and the availability of aggregate rather than patient individually collected data. Given that we used excess deaths and positivity rates, the findings are relatively robust to ascertainment of both outcomes and exposures. We have however used data from the UK, which has reported the highest numbers of these cases for the statistical analysis. It is essential that cases of paediatric hepatitis of unknown aetiology have comprehensive investigation, including SARS-CoV-2 serology, which has not been reported or done systematically*."

We also include information about the genetic association with cases which also associates with COVID-19 asymptomaticity, as follows:
*"One study found hepatitis cases had 12-fold greater odds of carrying the HLA-DRB1*04:01 allele [25]. This allele is associated with autoimmune conditions[26] and consistently associated with milder or asymptomatic infection with SARS-CoV-2 compared to controls [27-29] This allele has recently been shown to enhance the effect of HLA-B*15:01, which is associated with asymptomaticity following COVID infection, and a cross-reactive T cell response to SARS-CoV-2, providing a potential mechanism for this association.[30] It is plausible that this allele or the haplotype it is present on is involved in a potentiated immune response to SARS-CoV-2, which may also explain its enrichment in case series of hepatitis, if SARS-CoV-2 infection is on the causal pathway."*

The Bradford-Hill criteria are useful and raise important questions. 1) Strength – how strong are the associations between COVID-19 infection and acute hepatitis? 2) Consistency, was the 3-4 month link between COVID-19 and acute hepatitis seen in other countries and in later COVID-19 waves? 3) Specificity, essentially, all children were exposed or infected with SARS-COV-2, yet only a very small number developed acute hepatitis; this means if SARS-COV-2 is a causal factor, it is extremely non-specific.

As highlighted in our re-review of the recent literature, we discuss a hypothesis that SARS-CoV-2 or AAV-2 may be necessary but not sufficient for this phenotype (hence the lack of specificity observed with both in studies), and that it may occur with a genetic specific predisposition to immune dysregulation, or with increased susceptibility or re-activation of AAV-2 (potentially more likely post-COVID), or may require re-infection. We are not able to assess the exact conditions that lead to this phenotype; however current evidence suggests that AAV-2 is enriched in tissues of cases, and is found within hepatocytes and that cases are enriched for the HLA DRB 0401 allele (which is also shown to be associated with COVID asymptomaticity). This implies a potentially complex causal framework which would require further investigation, including biological studies of T-cell reactivity to SARS-CoV-2 among patients and controls. Recent studies have suggested that HLA-DR and HLA-B alleles lie on the same haplotype and are associated with asymptomaticity of COVID-19 potentially mediated by greater T cell cross-reactivity to SARS-CoV-2. It is likely that multiple factors play a role, and a single factor may not be sufficient to cause disease.

We outline this in the Discussion, as follows:
"*For SARS-CoV-2, there is evidence in the literature for temporality, strength of association, biological plausibility and analogy. Importantly the plausible biological mechanisms include a post-infectious inflammatory pathology, possibly related to MIS-C, or auto-immune hepatitis, as have been reported previously. Central to assessing SARS-CoV-2 as a possible cause of hepatitis is whether or not hepatitis (if related) is an acute or delayed complication. The temporality analysis is consistent with a delayed, post-infection hepatitis, consistent with higher seroprevalence in cases compared to controls reported in some studies (statistically significantly higher in one,[8] and not statistically significant in another).[31] If this is a delayed presentation, it is likely an immunological mechanism rather than a direct viral mechanism, and so parameters that rely on detecting the virus or quantifying the patient's viral load are not relevant. The temporal association is seen strongly on a population level, when observing the pattern of the Omicron wave and the subsequent hepatitis wave, noting that the rate of Omicron infections was highest in children compared to other age groups,[32,33] and substantially higher than previous waves. Therefore a rare complication like hepatitis may have only become apparent during the first Omicron wave due to the much higher rate of infections in children.[34] The peak in 5-17 year-olds also occurred later than in adults during the Omicron wave, potentially reflecting school closure during December followed by accelerating spread after school opening in January.[33] We note that there have not been continuing cases of hepatitis of unknown cause identified subsequent to the 2022 spring cluster. This may potentially be similar to the documented decline of MIS-C observed following the first omicron wave,[22] given most children in several countries (the UK, US) were exposed by this point, and re-infections may be less likely to give rise to these post-infection inflammatory syndromes. We note that while the omicron wave was followed by identified paediatric hepatitis cases of unknown cause in many countries, this did not occur in all countries with large omicron waves (e.g. Australia, New Zealand); we therefore consider the consistency criteria only partially met. Whether this represents lack of ascertainment due to gaps in surveillance, or inability to pick up rare signals in smaller populations, or all causal conditions for hepatitis of unknown cause not being met in these regions is unclear*."

"*We note that SARS-CoV-2 has been associated with immune dysregulation, including activation of latent viruses (e.g. EBV[9]), and increased risk of infection several months post-COVID.[10,11] It is plausible that past infection may activate latent viruses (like HAdv and HHV-6, and AAV-2), or that SARS-CoV-2 superantigens may sensitise the host to other viral infections (e.g. AAV-2) and that hepatitis of unknown cause may reflect an interaction between the two agents.[12,13] Another factor suggested that may have altered conditions are mitigation measures in 2020-2021 which may have led to concurrent surges of hAdv and AAV-2, leading to much higher levels of infection, with manifestation of a rare syndrome not identified before. However, we consider this unlikely given significant numbers of cases (12 for a population of 590,000 under 5s)[20] were seen even in countries like Sweden, where mitigations were limited, and there was very little suppression of seasonal influenza, unlike in many other countries where this was considerably suppressed*.[21]"

Many modern epidemiological methods have been developed to investigate exactly this sort of causal question, e.g. case only designs, instrumental variable analysis, difference in

difference, and other forms of natural experiment. These may be able to provide more credible answer to these hypotheses.

We acknowledge the role of other causal frameworks to answer this question. However, we are limited in the literature and data that are available to us. We are not designing a new study to examine this, but rather reviewing the existing data to help provide insights into the potential causality of these associations. With lack of adequate data (e.g. genetic, instrument variable data) to apply to the aforementioned study designs, and the rareness of this phenotype make it difficult to use these methods for assessing causality. These would require study designs that to our knowledge have not been used in the assessment of this association. We agree that a case-only design would allow a further examination of causality through an examination of aetiological heterogeneity. However, to our sample sizes of all studies to date have been very small, and studies have not usually reported on multiple individual exposures (e.g. to assess an interaction between SARS-CoV-2 and AAV-2). Furthermore, assessment of exposure to SARS-CoV-2 in young children is further complicated by serology often being negative and a poor proxy for past infection compared with adults. Little work has been done assessing SARS-CoV-2 exposure among samples beyond doing serology. A difference in difference approach would also require a characterisation of outcomes among those exposed to SARS-CoV-2 and hAdv +/- AAV-2. Given the lack of information on exposures and the outcomes of individuals exposed to these different viruses, it has not been possible to use these approaches, which we agree would provide a better causal framework.

We discuss this in our limitations as follows:

"*There were limitations to this study. We reviewed current observational evidence and scored criteria for causality and the evidence supporting each to assess this within a causal framework.* ***Given our reliance on limited observational data, we are unable to prove causality.*** *Limitations of studies include biases in hepatitis case ascertainment, small number of studies, small sample sizes and with inadequate matching where case-control analysis was done. In addition, lack of routine COVID testing and absence of complete serological data across countries, were a limitation. Furthermore, as we did not have the data on individual EDs included in the study, we were unable to assess the representativeness of UKHSA data across the England population. We were also unable to examine the evidence for an interaction between the two exposures, i.e. SARS-CoV-2 leading to an increase in susceptibility to infection by HAdv and/or AAV-2, as studies assessing both of these are currently very limited, and assessment of this criteria would likely require functional studies that currently do not exist. Additionally, our statistical modelling was limited by small sample sizes, the noisiness of routine A&E surveillance data, and the availability of aggregate rather than patient individually collected data. Given that we used excess hepatitis A&E attendances and positivity rates, the findings are relatively robust to ascertainment of both outcomes and exposures. We have used data from the England, which has reported the highest numbers of these cases for the statistical analysis. It is essential that cases of paediatric hepatitis of unknown aetiology have comprehensive investigation, including SARS-CoV-2 serology, which has not been reported or done systematically."*

VERSION 2 – REVIEW

**REVIEWER NAME**	***Harish PEMDE***
**REVIEWER AFFILIATION**	Kalawati Saran Children's Hospital, Pediatrics

**REVIEWER CONFLICT OF INTEREST**	
**DATE REVIEW RETURNED**	18-Mar-2024

**GENERAL COMMENTS**	This is a very good study using a rigorous scientific method.
